# Impacts of Whole-Body Vibration on Muscle Strength, Power, and Endurance in Older Adults: A Systematic Review and Meta-Analysis

**DOI:** 10.3390/jcm12134467

**Published:** 2023-07-03

**Authors:** Raphael Gonçalves de Oliveira, Heloísa Maria Elaine Licha Coutinho, Maiara Naldi Mansano Martins, Mario Bernardo-Filho, Danúbia da Cunha de Sá-Caputo, Laís Campos de Oliveira, Redha Taiar

**Affiliations:** 1 Postgraduate Program in Human Movement Sciences, Universidade Estadual do Norte do Paraná, Jacarezinho 86400-000, PR, Brazil; rgoliveira@uenp.edu.br (R.G.d.O.); maiaranaldi@outlook.com (M.N.M.M.); oliveiralc@uenp.edu.br (L.C.d.O.); 2 Postgraduate Program in Physical Exercise in Health Promotion, Universidade Norte do Paraná, Londrina 86041-120, PR, Brazil; heloisalicha54@gmail.com; 3 Laboratory of Mechanical Vibrations and Integrative Practices, Departamento de Biofísica e Biometria, Instituto de Biologia Roberto Alcantara Gomes, Policlínica Universitária Piquet Carneiro, Universidade do Estado do Rio de Janeiro, Rio de Janeiro 20950-003, RJ, Brazil; bernardofilhom@gmail.com (M.B.-F.); dradanubia@gmail.com (D.d.C.d.S.-C.); 4 MATériaux et Ingénierie Mécanique (MATIM), Université de Reims Champagne-Ardenne, 51100 Reims, France

**Keywords:** mechanical vibration, exercise, musculoskeletal fitness

## Abstract

Background: Randomized clinical trials (RCTs) were conducted to identify the effectiveness of whole-body vibration (WBV) on strength, power, and muscular endurance in older adults. However, the results of different studies are contradictory. Objective: To verify the impacts of the WBV on strength, power, and muscular endurance in older adults. Methods: The search was carried out in PubMed, Embase, CENTRAL, CINAHL, SPORTDiscus, Web of Science, LILACS and PEDro databases. Methodological quality was assessed using the PEdro scale. Meta-analysis calculations were performed using the standardized mean difference, comparing WBV with control groups and WBV with other types of exercise. Results: Thirty-four studies were included in the current systematic review. Most studies (56%) had low methodological quality (PEDro score < 6). WBV, compared with control groups, has significant effects on muscle strength of knee extensors and flexors, lower limb extensors, and ankle plantar flexors. There were no differences between WBV and other types of exercise. Subgroup analyzes demonstrated that, in general, the significant results observed in the primary analyzes were not dependent on body position during vibration, kind of vibration, cumulative dose or magnitude of WBV. Conclusion: WBV was effective in increasing lower limb muscle strength. However, no significant results were observed for upper limb strength, lower limb power, and lower and upper limb muscle endurance in older adults. However, more studies are needed to better understand the physiological impacts of WBV in older.

## 1. Introduction

Randomized clinical trials (RCTs) aiming to verify the effects of whole-body vibration (WBV) on muscle strength, power and endurance in older adults have been published over the past 23 years [1,2,3,4,5,6,7,8,9,10,11,12,13,14,15,16,17,18,19,20,21,22,23,24,25,26,27,28,29,30,31,32,33,34,35]. At the same time, systematic review studies with meta-analysis on the same subject have been conducted. However, with inconclusive results to date, although, for some variables, such as the muscle strength of the knee extensors, most studies corroborate that WBV enables significant effects [36,37,38,39,40,41].

The interest in the WBV intervention is due to the fact that it requires little effort and motivation from the practitioner. In addition, a low exposure time (approximately 5–15 min per session) is required, which is an interesting option when the practice of conventional physical exercises cannot be carried out or in addition to it. Some studies have demonstrated similar improvements in knee extensor muscle strength [30] and countermovement jumping [32] after older adults have undergone 12 months of WBV or multicomponent training. WBV performed in addition to conventional muscular resistance exercises allowed, after eight weeks, a significant gain in the isokinetic peak torque of the ankle plantar flexors compared to the same type of exercise performed without WBV [33]. However, other RCTs found no effect of WBV on muscle strength, power, and endurance in older adults [8,22,23].

The mechanisms by which WBV can promote improvement in neuromuscular fitness include neural adaptations due to increased muscle activation, provided by greater excitatory input from muscle spindles exposed to vibration. This phenomenon was called the tonic vibration stretch reflex [42]. As a result, the neuromuscular adaptation induced by WBV can induce physiological adaptations similar to those observed in conventional endurance and explosive strength training [43].

Although interventions involving WBV are relatively simple to administer, considering that the individual should only remain on the oscillating plate of the vibrating platform, many parameters can be used in intervention protocols, such as vibration frequency in Hz, peak-to-peak displacement in mm, exposure time per session, weekly frequency, body positioning, among others. The simple adjustment of these parameters may be enough to represent the observation or not of significant effects, which must be considered when interpreting RCTs results [44]. Particularly, the interest in this type of intervention in older adults is increasing, considering that this age group suffers most from the loss of strength, power, and muscular resistance, resulting in less functional autonomy. Older adults with compromised functional autonomy perform fewer tasks of daily living, leading to a vicious cycle of decreasing autonomy due to reduced neuromusculoskeletal fitness. This cycle substantially increases the risk of sarcopenia, falls, fractures, hospitalization, and death [45]. In this scenario, interventions such as WBV can be configured as an efficient intervention strategy by delaying or reversing the loss of neuromusculoskeletal fitness in older adults [46]. Given new RCTs published in recent years and the inconclusive results of previous systematic reviews, an updated systematic review is necessary. Thus, with this issue in mind, this study aimed to verify the impacts of the WBV on strength, power, and muscular endurance in older adults.

## 2. Methods

This study was prospectively registered in PROSPERO (CRD42022321582). The report followed the recommendations of the PRISMA statement [47], while for the planning, conduction and interpretation of the results, the recommendations of the Cochrane Handbook for Systematic Reviews of Interventions [48] were followed.

### 2.1. Eligibility Criteria

Inclusion criteria: (a) RCTs; (b) intervention with WBV using a vibrating platform; (c) studies that evaluated (outcome) at least one measure directed to muscle strength, power, or endurance; (d) studies with older adults (≥60 years).

Exclusion criteria: (a) WBV associated with another form of intervention (e.g. resistance training), without having a comparison group with this same form of intervention; (b) studies that evaluated only the effectiveness of one session (acute effect); (c) studies with duplicate information in another RCT already included; (d) studies with a sample composed of people diagnosed with neurodegenerative diseases (e.g., Alzheimer’s, Parkinson’s, multiple sclerosis).

### 2.2. Databases and Search Strategy

The search was carried out in the following databases: PubMed, EMBASE, CENTRAL, CINAHL, SPORTDiscus, Web of Science, LILACS and PEDro, without using a filter for publication date or language. Two clinical trial registry databases were consulted (clinicaltrials.gov and https://www.who.int (accessed on 1 May 2022)) to find potential unpublished studies. The search took place on 1 May 2022.

The following keywords were used in the search strategy: (“aged” OR “aging” OR “ageing” OR “elderly” OR “older people” OR “older adults” OR “older adult” OR “older women” OR “older men” OR “geriatric” OR “geriatrics” OR “frail elderly” OR “elder” OR “elders” OR “aged people”) AND (“whole body vibration” OR “WBV” OR “vibration” OR “vibration therapy” OR “vibration training” OR “oscillating platforms” OR “vibrating platform” OR “vibration device” OR “mechanical vibration”) AND (“muscle strength” OR “muscle strength dynamometer” OR “strengthening” OR “strength” OR “torque” OR “maximal voluntary contraction” OR “1RM” OR “one repetition maximum” OR “1 repetition maximum” OR “muscular endurance” OR “muscle endurance” OR “isometric” OR “isometry” OR “muscle power” OR “power” OR “muscular power” OR “power output” OR “functional tests” OR “functional autonomy” OR “functional mobility”). The complete strategy used in each database can be accessed in Supplementary S1.

The research question was, “Does the whole-body vibration impact muscle strength, power, and endurance in older adults? The PICO method [48] was used to structure the bibliographic search: P (population) = older adults; I (intervention) = WBV; C (comparison) = no intervention or other types of intervention; O (outcome) = muscle strength, power, or endurance.

### 2.3. Selection of Studies

A reviewer (RGO) carried out the initial search strategy in the databases, extracting the titles and abstracts. This same reviewer extracted duplicates using online software (https://www.rayyan.ai/ (accessed on 15 May 2022)). Subsequently, two reviewers (HMELC and MNMM), with the aid of this same software, blindly read the titles and abstracts. Subsequently, the potentially eligible reports were read in full by these same reviewers blindly. Whenever there was disagreement between the reviewers, a third party (LCO) was asked to resolve the impasse. A manual search of the reference lists was performed for eligible articles to find additional studies. The references of published systematic reviews were also checked to locate studies that could not be identified in the searched databases.

### 2.4. Data Extraction

Two reviewers (HMELC and MNMM) used the same standardized form to blindly extract information from each study that met the eligibility criteria. When there was divergence in the extracted information, a third reviewer (RGO) was asked to resolve the impasse. The data extracted from each study were: (a) name of the first author, year of publication and geographic location; (b) the total number of participants and in each group, sex, housing (resident in the community or an institution for the elderly) and health condition (e.g., healthy, sarcopenia, low functionality, etc.); (c) mean age in each group; (d) time of exposure to WBV; (e) parameters used in the WBV (frequency [Hz], peak-to-peak displacement [mm] and magnitude [g]) and type of vibration (synchronous or side-alternating); (f) body positioning during WBV; (g) activities carried out by other intervention groups; (h) activities of the control group; (i) assessments performed for muscle strength, power and/or endurance; (j) possible differences between groups after the intervention period; (k) adverse events; (l) loss of participants during the study and frequency of participation during interventions.

### 2.5. Assessment of the Methodological Quality of Studies

To assess the methodological quality, the PEDro scale (Physiotherapy Evidence Database) [49] was used. Whenever available, scores were extracted from the PEDro platform database (https://pedro.org.au/ (accessed on 20 July 2022)). When studies were not included in the database, two reviewers (HMELC and MNMM) blindly evaluated the work, with disagreements being resolved by a third reviewer (LCO). The PEDro scale considers the internal validity and the sufficiency of statistical information of the studies, presenting 11 questions. The first question is not scored (related to the external validity of the study). Thus, each study can establish a score of 0–10 points. Studies with scores <6 are considered of low methodological quality. Maher et al. [49] demonstrated good inter-rater reliability, with an intra-class correlation coefficient of 0.68 when using consensus ratings generated by two or three independent raters on the PEDro scale.

### 2.6. Definition

WBV was an intervention in which mechanical vibrations provided by a vibrating platform is transmitted to the human body that is in contact with the base of the vibrating platform. In general, vibrating platforms allow the configuration of two parameters: frequency expressed in hertz (Hz) and peak-to-peak displacement in millimeters (mm). The magnitude of the intervention is expressed in gravitational acceleration in g. Acceleration can be obtained by an accelerometer or estimated using the formula: m/s^2^ = 2·π^2^·f^2^·m, where “f” is the frequency in Hz and “m” is the expressed peak-to-peak displacement in meters (gravitational acceleration: 1 g = 9.8 m/s^2^) [50].

There are two main types of vibrating platforms: (1) synchronous and triplanar, also known as vertical; (2) side-alternating displacement of the base. In the vertical, the mechanical vibration occurs in a predominantly vertical direction, synchronously throughout the base of the oscillating base or a resultant of the movement of the base in three plans. In the second, the mechanical vibration occurs through a central rotation axis, causing the right and left sides to alternate horizontally like a seesaw [51].

External factors also impact the intensity of vibration, such as body positioning (knees extended, flexed, or performing muscle strengthening exercises) and exposure time (minutes per session, rest time, weekly frequency and total intervention time). If multiplied, the three factors determining the exposure time make it possible to estimate the cumulative dose of WBV to which the participant was exposed during the entire intervention period.

### 2.7. Data Analysis

For the meta-analysis, the measure of effect was the post-intervention standardized mean difference (SMD) between the WBV vs. control or WBV vs. conventional exercises. The Cochrane Q test for heterogeneity was performed and considered statistically significant if *p* ≤ 0.10. Heterogeneity was also quantified with the I^2^ statistic, where 0–40% may not be important, 30–60% may represent moderate heterogeneity, 50–90% may represent great heterogeneity, and 75–100% is defined as considerable heterogeneity [48]. Fixed effects models were used when there was no statistically significant heterogeneity. Otherwise, random effects models were used. Values referring to the effect of WBV on the outcomes of interest were only considered statistically significant when *p* < 0.05. To assess the risk of publication bias, a funnel plot was used when there were ≥10 RCTs in a meta-analysis. A sensitivity analysis was performed to verify whether studies of low methodological quality would influence the results of the primary analysis. All analyzes were performed using the Review Manager (RevMan) [Computer program], version 5.4, Copenhagen: The Nordic Cochrane Centre, The Cochrane Collaboration.

## 3. Results

### 3.1. Qualitative Synthesis of Studies

**Figure 1:**
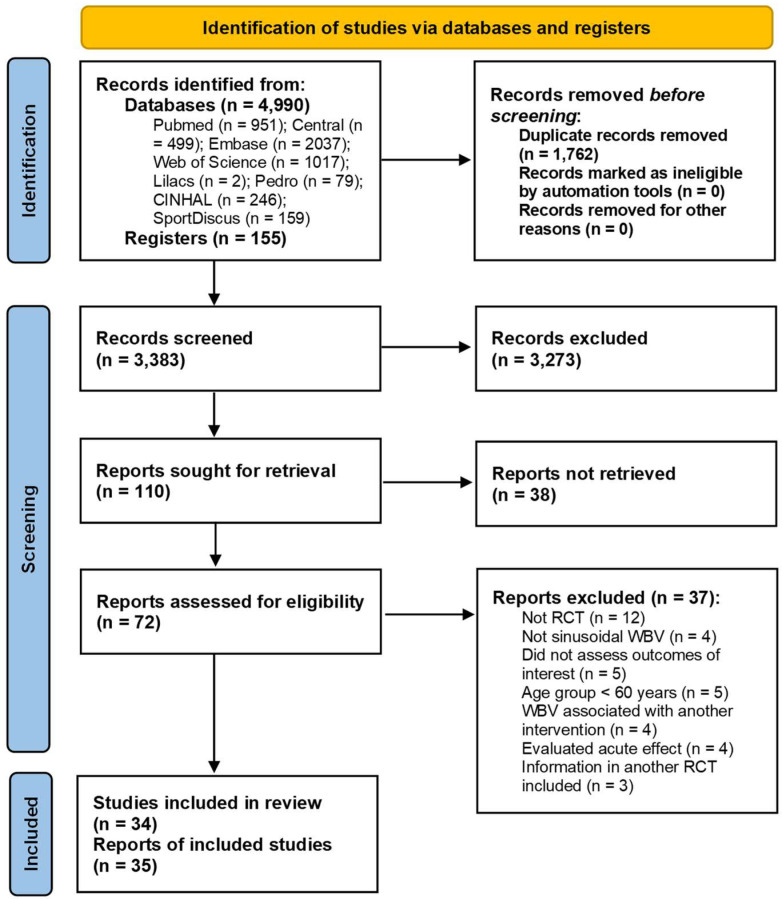
PRISMA 2020 flow diagram.

It was possible to identify 4990 potentially relevant records in the databases and 155 clinical trial record protocols. After removing 1762 duplicates, 3383 titles and abstracts were read, of which 3273 were excluded for not meeting the eligibility criteria. Of the remaining 110 reports, 38 were not retrieved, mainly because they were abstracts published in conferences (18 reports) or protocols from clinical trial registries (16 registries) without a full text with available results. The complete list of studies not retrieved is available in Supplementary Table S1. Thus, 72 reports were accessed in full, of which 37 did not meet the eligibility criteria (a complete list of studies excluded at this stage is available in Supplementary Table S2). The reasons for exclusion were: (a) not being an RCT (12 reports); (b) non-use of a sinusoidal vibrating platform (4 reports); (c) did not evaluate the outcomes of interest (5 reports); (d) participants aged < 60 years (5 reports). (e) WBV associated with another intervention (4 reports); (f) assessment of the acute effect only (4 reports); (g) information already taken from another ECR included (3 reports). Thus, 35 reports were included in the systematic review, comprising 34 studies (the reports by Wei et al. [9] and Wei et al. [10] make up the same study). Figure 1 illustrates the phase of identification, screening and inclusion of studies.

The 35 reports included in this systematic review (Table 1 [1,2,3,4,5,6,7,8,9,10,11,12,13,14,15,16,17,18,19,20,21,22,23,24,25,26,27,28,29,30,31,32,33,34,35]) were published between the years 2000 and 2021, with the majority being developed in Europe (51.4%), followed by Asia (31.4%), Oceania (8.6%), South America (5.7%) and North America (2.9%). The number of participants in each study ranged from 15 [20] to 596 [18]. In most studies, participants lived in the community (61.8%) and were healthy (73.5%). The mean age ranged from 64.4 ± 2.8 [5] to 89.5 ± 4.4 years [3]. The total duration of the studies ranged from 10 consecutive days [7] to 18 months [18,25], while the most used weekly frequency was 3× (58.8%), followed by 2× (26.5%). The duration of each session ranged from 2 min [2] to 30 min [13], while the average cumulative dose of WBV ranged from 23 min and 30 s [8] to 7800 min [18]. Regarding the WBV parameters, many studies alternated the values of frequency and peak-to-peak displacement throughout the intervention, with values ranging from 5 Hz [1] to 60 Hz [9,10] and < 0.1 mm [18] up to 14 mm [35], respectively. These parameters resulted in magnitudes between 0.1 g [21] and 20.5 g [35].

Regarding body positioning, most studies administered muscle-strengthening exercises for the lower limbs during WBV (61.8%). Other studies adopted a static posture, such as semi-flexed knees (31.4%), single leg support with semi-flexed knees (2.9%) and extended knees (2.9%). Almost half of the studies (47%) compared WBV with another form of intervention (mainly conventional exercises), while control groups were adopted by most studies (79%). The muscle strength outcome was assessed by 70.6% of the studies, muscle power by 47.1% and muscle endurance by 23.5%. Regarding the results reported in the original publication, among those that evaluated muscle strength, 11 studies identified that the WBV allowed a significant increase when compared to control groups for muscle strength of knee extensors [3,7,10,12,18,21,30,32] and flexors [3,12], ankle dorciflexors [3] and ankle planti-flexors [33], lower limb isometry [6,28] and back-leg-chest dynamometry [7]. For muscle power, six studies found that WBV was superior to control groups on the five-times-sit-to-stand [3,6,9,31], knee extensors [14] and countermovement jump [32]. In the muscular endurance outcome, a study demonstrated that WBV was superior to the control group for the 30-s sit-to-stand [17].

Adverse events due to WBV were reported by six studies (17.6%) and involved: injuries due to a fall, complication of pre-existing arthritis, edema and back pain [14], pain in the knees and lumbar spine, itching, erythema and edema [16], lower limb pain, dizziness and hypertension [18], groin pain and airway infection [34], forefoot inflammation [35] and other disorders [3]. Fifty percentage of the studies declared that no adverse events were observed during the intervention period with WBV, while 11 studies (32.4%) did not report this condition. Thirty studies reported adherence to interventions, which averaged 86.6% in the groups that used WBV. Finally, the frequency of participation in interventions was reported by 19 studies, with an average of 84.8% in the WBV groups.

### 3.2. Methodological Quality of Studies

Table 2 also presents the methodological quality of the studies scored using the PEDro scale. Of the 34 studies included in the systematic review, 44% had high methodological quality (PEDro score ≥ 6). Considering all studies, the mean score was 5.5 ± 1.4 points (range 2 to 8 points).

### 3.3. Quantitative Synthesis of Studies (Meta-Analysis)

#### 3.3.1. Primary Analysis

##### Muscle Strength

**Figure 2:**
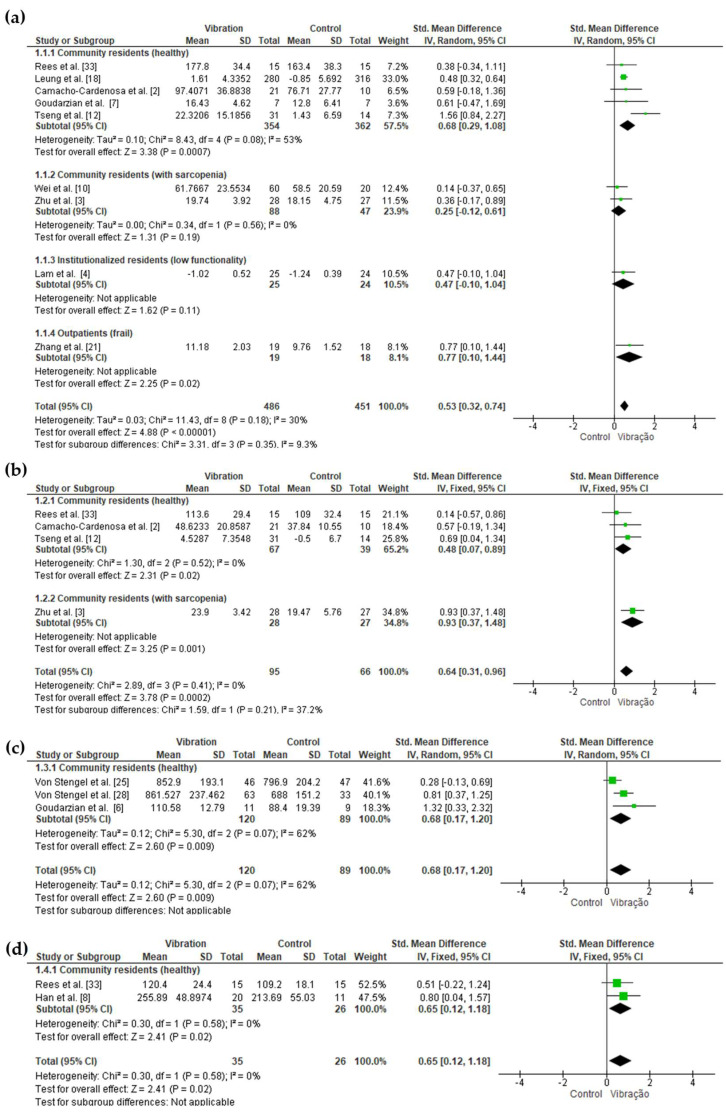
Primary analysis comparing the effectiveness of WBV vs. control groups for muscle strength: (**a**) knee extensors; (**b**) knee flexors; (**c**) leg extensors; (**d**) ankle plantar-flexors.

In the primary analysis, we observed a significant difference with moderate effect size in favor of WBV for muscle strength of knee extensors and flexors, leg extensors and ankle plantar flexors (Table 3 and Figure 2). For the other strength variables, no significant differences were observed between the groups (Supplementary Figure S1). In the forest plot figures of the primary analyses, subgroup analyzes were carried out considering the place of residence (community or institution) and the health condition (sarcopenia, low functionality, and frailty). Overall, all subgroup analyzes for these categories followed the main analysis, except for knee extensor muscle strength among community residents with sarcopenia, in which there was no significant difference between WBV and control, in addition to the muscle strength analysis of the ankle dorsiflexors, which in this case showed a significant difference in favor of WBV, with a moderate effect size in community residents with sarcopenia.

##### Muscle Power

For muscle power, assessed using the five-times-sit-to-stand or countermovement jump test, no significant difference was observed between WBV and control groups in the primary analysis (Table 3). This result was independent of the older adult’s place of residence or health condition (Supplementary Figure S2).

##### Muscle Endurance

For muscular endurance, assessed using the 30-s sit-to-stand or 30-s arm curl test, no significant difference was observed between WBV and control groups in the primary analysis (Table 3). This result was also independent of the older adult’s place of residence or health condition (Supplementary Figure S3).

#### 3.3.2. Sensitivity Analysis

To verify a possible influence of low methodological quality on the results of the primary analysis, sensitivity analyzes were performed. In those studies, scores < 6 on the PEDro scale were excluded. In this case, in five analyzes (knee flexors, ankle dorsiflexors, ankle plantar-flexors, hip flexors and 30-s arm curl), there were only studies of low methodological quality, which did not allow for a sensitivity analysis. For the other analyzes (knee extensors, leg extensors, handgrip, five-times-sit-to-stand, countermovement jump, 30-s sit-to-stand), the results did not change after removing the low-quality studies methodological (Supplementary Figure S4).

#### 3.3.3. WBV vs. Other Exercise Modalities

Analyzes comparing WBV with other exercise modalities did not identify any significant difference in muscle strength outcomes (knee extensors and flexors, ankle dorsiflexors, hip flexors and handgrip) (Supplementary Figure S5). No muscle power and endurance outcome study compared WBV with other exercise modalities.

#### 3.3.4. Static Positioning vs. Dynamic Exercises during WBV

When only studies in which participants maintained a static position during WBV were grouped, significant results were observed for muscle strength of knee extensors and flexors, in addition to ankle dorciflexors, but not for strength of hip flexors, handgrip, five-times-sit-to-stand and 30-s sit-to-stand (Supplementary Figure S6).

When considering only the studies in which the participant performed muscle strengthening exercises during WBV, significant results were observed for muscle strength of knee extensors, leg extensors and ankle plantar-flexors, but not for muscle strength of knee flexors, ankle dorciflexors, hip flexors and handgrip. There were also no differences for the tests of five-times-sit-to-stand, countermovement jump, 30-s sit-to-stand and 30-s arm curl (Supplementary Figure S7).

#### 3.3.5. Vibration Type (Synchronous vs. Side-Alternating)

When it was grouped only the studies that used synchronous (and/or triplanar) vibration, we identified that the WBV, compared to control groups, allowed a significant increase in the muscle strength of the knee extensors and ankle plantar flexors, but not for the muscle strength of the leg extensors and hip flexors, handgrip, five-times-sit-to-stand, countermovement jump, 30-s sit-to-stand and 30-s arm curl (Supplementary Figure S8).

For side-alternating, WBV compared with control groups allowed a significant increase in muscle strength of knee extensors and flexors and leg extensors, but not for muscle strength of ankle plantar flexors and ankle dorsiflexors, hip flexors, handgrip, five-times-sit-to-stand, countermovement jump, and 30-s sit-to-stand (Supplementary Figure S9).

#### 3.3.6. Cumulative Dose of WBV

In the subgroup analyzes in which the objective was linked to verifying the effectiveness of WBV according to the cumulative dose administered (total time of WBV administration throughout the entire intervention), we grouped studies with low cumulative dose (≤44 min) vs. high (>144 min), considering, as a cutoff point, the median cumulative dose calculated through the studies of the present systematic review. WBV administered at a low cumulative dose allowed significant effects on the muscle strength of the knee extensors and flexors, leg extensors and ankle plantar flexors, but not for handgrip, five-time sit-to-stand, 30-s sit-to-stand and 30-s arm curl (Supplementary Figure S10).

When grouping studies with high cumulative doses, WBV allowed significant effects on muscle strength of knee extensors and leg extensors but not for muscle strength of knee flexors, ankle plantar flexors, ankle dorsiflexors, hip flexors, handgrip, five-times-sit-to-stand, countermovement jump, 30-s sit-to-stand and 30-s arm curl (Supplementary Figure S11).

#### 3.3.7. WBV Magnitude

In the subgroup analyzes in which we aimed to observe the impact of the magnitude of WBV, we grouped the studies into low (≤4.4 g) and high (>4.4 g) magnitude, considering, as a cutoff point, the median calculated through of the studies in this systematic review. Compared with control groups, the low magnitude of WBV allowed significant effects on the muscle strength of the knee extensors and flexors but not for the leg extensors, handgrip, five-times-sit-to-stand, countermovement jump, 30-s sit-to-stand and 30-s arm curl (Supplementary Figure S12).

When only high-magnitude studies were grouped, WBV compared to the control groups allowed a significant increase in muscle strength of leg extensors and ankle plantar flexors, but not for muscle strength of knee extensors and flexors, ankle dorsiflexors, hip flexors, handgrip, five-times-sit-to-stand, countermovement jump, 30-s sit-to-stand and 30-s arm curl (Supplementary Figure S13).

## 4. Discussion

### 4.1. Summary of Main Results

The aim was to verify the effectiveness of WBV on muscle strength, power, and endurance in older adults. Although our qualitative synthesis demonstrated that WBV could improve the three variables, our meta-analysis calculations only confirmed an increase in lower limb muscle strength (knee extensors and flexors, lower limb extensors and ankle plantar flexors) compared to control groups. In general, the observed results, whether significant or not for each outcome/parameter analyzed, were independent of the participant’s place of residence and health condition. Regarding possible differences between WBV and other physical exercise modalities, the individual studies in our systematic review did not point to significant differences, as confirmed by the meta-analysis calculations. Regarding the best WBV parameters for increasing lower limb muscle strength in older adults, we observed that, in general, the significant effects are maintained, regardless of body positioning (static or performing muscle strengthening exercises), type of vibration (synchronous or side-alternating), cumulative dose (low or high), and magnitude (low or high).

### 4.2. Agreements and Disagreements with Other Studies

Some systematic review studies with meta-analysis have been published with this same theme in older people [36,37,38,39,40,41]. Lau et al. [36] found moderately significant effects of WBV compared to control groups on knee extensor muscle strength (including 2 RCTs), lower limb extensors (including 2 RCTs), countermovement jump (including 2 RCTs) and five-times-sit-to-stand (including 3 RCTs), while no difference was observed between WBV and conventional exercises. Osawa et al. [37], when verifying the effects of WBV vs. control groups, observed moderately significant effects on knee extensor muscle strength (including 4 RCTs) and countermovement jump (including 2 RCTs), with moderate effect sizes. Rogan et al. [38], when observing the effects of WBV compared to control groups, identified a moderately significant effect for isometric muscle strength (including 14 RCTs). However, data from different body segments were combined in a single analysis. The authors found no significant differences between WBV and control groups for dynamic muscle strength and power (including 6 RCTs each). For these same analyses, no differences were identified between WBV and conventional exercises.

In the meta-analysis by Pessoa et al. [39], significant effects of WBV compared to control groups on muscle strength were not observed (including six studies). However, the evaluated body segment was not specified. Wu et al. [40] observed moderately significant effects on lower limb muscle strength (including 2 RCTs) and muscle power by the five-times-sit-to-stand (including 2 RCTs) in favor of WBV compared with control groups. Šarabon et al. [41] observed a significant effect of great magnitude on muscle strength of the knee extensors when comparing WBV with control groups (including 8 RCTs).

It is observed that most of the systematic reviews with meta-analysis carried out up to the present moment identified significant effects in favor of the WBV compared with control groups on the muscle strength of the lower limbs, mainly the knee extensor musculature, which is the most tested, in line with the findings of the current study. In addition, in the current study, we observed significant effects on the knee flexor muscles, lower limb extensors and ankle plantiflexors, which are little or no measures explored by previous meta-analyses. A point of disagreement in relation to previous studies refers to motor tests related to muscle power, such as countermovement jumping [36,37] and five-times-sit-to-stand [36,40], for which the present study did not find any effect. Possibly, the difference is because the present meta-analysis included more RCTs in the different analyses.

An important differential of the current study concerning the systematic reviews and meta-analyses already carried out on the subject is the different subgroup analyses, which aimed to understand whether there are parameters that may eventually be more or less favorable for gaining strength, power and muscular endurance. This is because, eventually, in the primary analysis, significant results may not be observed since studies containing parameters that do not favor increased performance are “distorting” the real effect. Surprisingly, in the present study, in all subgroup analyses, the significant impact of WBV on lower limb muscle strength was maintained in at least two different lower limb muscle strength tests.

Another fact that draws attention is that no power variable and muscular resistance started to have statistical significance in the subgroup analyses. This demonstrates that the WBV did not allow the performance gain of these physical capacities regardless of the parameters used. In the current study, we tested four WBV parameters: body positioning (static or performing muscle-strengthening exercises); type of vibration (synchronous vs. side-alternating); cumulative dose (high vs. low); and magnitude (high vs. low). The literature has already shown that for other outcomes, such as bone mineral density, WBV enables greater effects when administered on platforms of the side-alternating type and positioning the participant with semi-flexed knees [52].

The number of RCTs included in each meta-analysis possibly influenced the observed results. For example, with regard to lower limb muscle power, WBV has been shown to provide acute effects [53,54]. In theory, if a single session enables significant effects, they should remain in the long term. However, this was not observed in our analyses, and it is important to carry out further studies on this topic.

Another important point to be debated is the impact of WBV on the neuromusculoskeletal fitness of the upper limbs. The present study observed that no significant impact occurred for handgrip strength and elbow flexor resistance, as assessed by the 30-s arm curl. For these two tests, it must be considered that the participants were standing on the vibrating platform. In this sense, little vibration is dissipated to the upper limbs. It has already been shown that WBV can impact the acute muscle power of upper limbs in young adults who perform elbow flexion exercises with their hands directly on the oscillatory plate [55]. However, the chronic effects of this placement remain unclear and need to be tested in long-term RCTs.

### 4.3. Quality of Evidence

More than half of the studies in our systematic review were from Europe (51.4%), and almost a third from Asia (31.4%). That is, there was no reasonable geographic distribution. Therefore, further studies on this topic are needed in other continents. Overall, the methodological quality of the RCTs included in the systematic review was low, with a mean of 5.5 ± 1.4 points, with 56% of the studies presenting scores <6 on the PEDro scale, which should be considered when interpreting the findings. No studies blinded therapists, and only two blinded participants [14,34]. In this case, it must be considered that in therapies whose stimuli are perceptible, it becomes very difficult to blind participants or therapists. However, it is unlikely that this bias could have influenced the outcome measures. Still, approximately half of the studies (53%) did not blind raters, 85% did not blindly allocate participants to their respective intervention groups, and 65% of the studies did not perform an intention-to-treat analysis. In addition, most studies used small sample sizes (≈10–20 participants per group), and the intervention time was ≤8 weeks in 41% of the studies.

In general, the low methodological quality of the studies included in this systematic review does not have a plausible explanation. Although sensitivity analysis showed that studies of low methodological quality did not influence the results for five meta-analyses (knee flexors, ankle dorsiflexors, ankle plantar flexors, hip flexors and 30-s arm curl), it was not possible to perform an analysis of sensitivity since all studies had low methodological quality. This must be considered when interpreting the results of these meta-analyses, which must be interpreted with caution.

### 4.4. Limitations of the Review Process

The current systematic review only included RCTs, which decreases the risk of bias. However, some studies did not disclose post-intervention results as mean and standard deviation, making their inclusion in meta-analysis calculations unfeasible. The search did not extend to all existing databases. However, we searched eight databases (PubMed, EMBASE, CENTRAL, CINAHL, SPORTDiscus, Web of Science, LILACS, and PEDro) and two clinical trial databases (clinicaltrials.gov (accessed on 1 May 2022) and apps.who.int/trialsearch/ (accessed on 1 May 2022)), aiming to find unpublished studies. In addition, we performed a thorough search of all bibliographic references of the studies included in the review in an attempt to find other RCTs. Finally, our meta-analyses were performed with few studies, and it was impossible to perform a visual inspection on funnel charts to identify possible publication bias.

## 5. Conclusions

### 5.1. Implications for Practice

WBV has been shown to enable significant effects on lower limb muscle strength in older adults, regardless of the parameters used, being an intervention option for this purpose. However, at this time, it is not possible to recommend using WBV to increase upper limb muscle strength, lower limb muscle power, and lower and upper limb muscle endurance.

### 5.2. Implications for Research

Future RCTs aiming to verify the effects of WBV on variables related to muscle strength, power and endurance in older adults should take greater care with methodological aspects, especially regarding rater blinding, hidden allocation, and handling of data from participants who discontinued the intervention. RCTs with more participants and longer intervention time are also needed. Finally, muscle power and endurance outcomes need to be further investigated.

## Figures and Tables

**Table 1:** Summary of the articles included in the systematic review.

Author, Year and Location	Total Number of Participants, Dwelling/Health Condition, Distribution by Sex and by Groups	Mean and SD (or Range) of the Age	Time of Exposure to Vibration and Mean Cumulative (MC)	Frequency (Hz), Peak to Peak Displacement (mm) and/or Magnitude (g) and Vibration Type	Position/Activity on the Vibrating Platform	Activities of Other Intervention Groups	Activities of the Control Group (CON)	Assessment of Muscle Strength, Power and/or Endurance	Differences between Groups Reported in the Original Publication (*p* < 0.05)	Adverse Events	Adherence and Compliance ‡
Genest et al., 2021 [1] Germany	47 community/osteoporosis: 47(m); 0(f) WBV = 13 RT = 11 QG = 10 SO = 13	All: 77.0 ± 6.1 WBV: 77.9 ± 6.2 RT: 75.9 ± 5.6 QG: 77.0 ± 7.9 SO: 77.2 ± 5.5	Six months 2× week 2 min 30 s–8 min MC: ≈ 429 min	5–25.5 Hz; 3 mm; 0.15–3.9 g * Side-alternating	Strengthening exercises for lower limbs	RT: 8 exercises focused on core strength (30 min, 2× week); QG: exercises of coordinated body posture, low-impact movements, breathing and meditation (45 min, 2× week); SO: use of back brace (180 min, 7× week)	-	Muscle strength: trunk flexion, trunk extension; handgrip Muscle power: 5TSTS	RT: improved (vs. QG) for trunk extension	No adverse event	Adherence: 100% Compliance: WBV: 83.2% RT: 71.3% QG: 65.1% SO: 85.2%
Camacho-Cardenosa et al., 2019 [2] Spain	31 community/healthy: 11(m); 20(f) NWBV = 11 HWBV = 10 CON = 10	NWBV: 70.2 ± 6.4 HWBV: 73.5 ± 4.7 CON: 73.4 ± 5.0	18 weeks 2× week 4 × 30 min (2 min total) MC: ≈ 72 min	12.6 Hz; 4 mm; 2.5 g Side-alternating	Semi-flexed knees	-	Usual routine	Muscle strength: peak torque of knee extensors and flexors (60°/s) Muscular endurance: total work of knee extensors and flexors (180°/s)	No difference	No adverse event	Adherence: 75% Compliance: NWBV: 100% HWBV: 91.4%
Zhu et al., 2019 [3] China	79 community/sarcopenic: 79(m); 0(f) WBV = 28 TC = 24 CON = 27	All: 88.6 (85–101) WBV: 89.5 ± 4.4 TC: 88.8 ± 3.7 CON: 87.5 ± 3.0	8 weeks 5× week 5 × 3 min (15 min total) MC: ≈ 600 min	12–16 Hz; 3–5 mm; 0.9–14.2 g * Side-alternating	Semi-flexed knees	TC: 10 min warm-up, 20 min practice, and 10 min relaxation (40 min, 5× week)	Usual routine	Muscle strength: handgrip and lower limbs (ankle dorciflexors, hip flexors, knee extensors and flexors) Muscle power: 5TSTS	WBV: improved (vs. CON) for 5TSTS, ankle dorciflexors, knee extensors and flexors; TC: improved (vs. CON) for 5TSTS, ankle dorciflexors, hip flexors and knee extensors	WBV: “other disorders” (7.1%) TC: low back pain (8.3%)	Unreported
Lam et al., 2018 [4] China	73 institutionalized/low functionality: 33(m); 40(f) WBV + CT = 25 CT = 24 CON = 24	All: 82.3 ± 7.3 WBV + CT: 84.0 ± 6.7 CT: 82.4 ± 7.6 CON: 80.3 ± 7.3	8 weeks 3× week 4 × 1 min (4 min total) MC: ≈ 96 min	30–40 Hz; 0.9 mm; 3.4–4.7 g Synchronous	Strengthening exercises for lower limbs	CT: warm-up, mobility, strengthening, balance, and cool-down exercises (60 min, 3× week)	CON: social and recreational activities that only involved the upper limbs	Muscle strength: knee extensors Muscle power: 5TSTS	CT: improved (vs. CON) for 5TSTS	No adverse event	Adherence: 85% Compliance: WBV + CT: 77.1% CT: 67.5% CON: 74.5%
Pessoa et al., 2018 [5] Brazil	31 community/healthy: 17(m); 14(f) WBV = 10 RT = 10 WBV + RT = 11	WBV: 66.4 ± 2.6 RT: 68.2 ± 2.4 WBV + RT: 64.9 ± 2.8	12 weeks 3× week 10 × 1 min (10 min total)–20 × 1 min (20 min total) MC: ≈ 540 min	35 Hz; 2–4 mm; 4.9–9.8 g * Synchronous	Semi-flexed knees	RT: strengthening exercises for upper and lower limbs (40 min, 3× week) plus WBV Sham WBV + RT: true WBV plus RT	-	Muscle strength: handgrip	No difference	No adverse event	Adherence: 91% Compliance: unreported
Goudarzian et al., 2017 [6] Iran	42 community/healthy: 42(m); 0 (f) WBV = 11 MT = 12 WBV + MT = 10 CON = 9	All: 68.0 ± 5.8 WBV: 66.6 ± 5.8 MT: 69.2 ± 3.9 WBV + MT: 67.8 ± 5.9 CON: 68.9 ± 7.5	8 weeks 3× week 6 × 45 s (4 min 30 s total)–6 × 80 s (8 min total) MC: ≈ 144 min	30–35 Hz; 5–8 mm; 9.0–19.7 g * Side-alternating	Strengthening exercises for lower limbs	MT: Relaxation techniques, with breathing and mental training (3× week)	Usual routine	Muscle strength: lower limb isometry (leg press dynamometer) Muscle power: 5TSTS	WBV, MT and WBV + MT: improved (vs. CON) for lower limb isometry and 5TSTS	Unreported	Adherence: 87.5% Compliance: unreported
Goudarzian et al., 2017 [7] Iran	22 institutionalized/healthy: 0(m); 22(f) WBV + P = 7 WBV + C = 8 CON = 7	All: 66.0 ± 5.0 WBV + P: 66.0 ± 4.6 WBV + C: 64.9 ± 3.4 CON: 68.0 ± 9.2	10 consecutive days 6 × 45 s (4 min 30 s total)–6 × 65 s (6 min 30 s total) MC: ≈ 33 min	30–35 Hz; 5 mm; 9–12.3 g * Side-alternating	Strengthening exercises for lower limbs	WBV + C: vibration associated with creatine supplementation (20 g/day [5 days] and 5 g/day [5 days])	Usual routine	Muscle strength: handgrip, knee extensors (1RM) and back-leg-chest	WBV + P and WBV + C: improved (vs. CON) for knee extensors (1RM) WBV + P: improved (vs. CON) for back-leg-chest	No adverse event	Adherence: unreported Compliance: 97.8%
Han et al., 2017 [8] Korea	40 community/healthy: 0(m); 40(f) WBV(I) = 13 WBV(E) = 12 CON = 15	All: 69.0 ± 4.0	8 weeks 1× week WBV(I): 3 × 30 s (90 s total)–8 × 30 s (4 min total), MC: ≈ 23 min 30 s WBV(E): 3 × 30 s (90 s total)–8 × 60 s (8 min total), MC: ≈ 39 min	WBV(I): 25–40 Hz, 1.1–2.5 mm, 1.4–8.0 g * WBV(E): 25–35 Hz, 1.1 mm, 1.4–2.7 g * Synchronous	Strengthening exercises for lower limbs	-	Usual routine	Muscle strength: isometric ankle plantar flexion	No difference	Unreported	Unreported
Wei et al., 2017 [9,10] Hong Kong	80 community/sarcopenic: 24(m); 56 (f) WBV(L) = 20 WBV(M) = 20 WBV(H) = 20 CON = 20	WBV(L): 78 (4) WBV(M): 75 (6) WBV(H): 74 (5) CON: 76 (6)	12 weeks 3× week WBV(L): 12 min, MC: ≈ 432 min WBV(M): 6 min, MC: ≈ 216 min WBV(H): 4 min, MC: ≈ 144 min	WBV(L): 20 Hz, 4 mm, 3.2 g WBV(M): 40 Hz, 4 mm, 12.9 g WBV(H): 60 Hz, 4 mm, 29.0 g Synchronous	Semi-flexed knees	-	Unreported	Muscle strength: isometric strength (90°) and peak torque of knee extensors (60°/s and 180°/s) Muscle power: 5TSTS	WBV(M): improved (vs. CON) for peak torque of knee extensors (180°/s) and 5TSTS	No adverse event	Adherence: 87.5% Compliance: unreported
Smith et al., 2016 [11] USA	60 institutionalized/healthy: 24(m); 36(f) WBV = 13 BD = 16 WBV + BD = 17 CON = 14	WBV: 82.2 ± 5.0 BD: 80.5 ± 6.2 WBV + BD: 83.4 ± 5.0 CON: 81.7 ± 5.7	12 weeks 2× week 3 min MC: ≈ 72 min	30 Hz; 2 mm; 3.6 g * Synchronous	Unipodal support with semi-flexed knees	BD: Muscle strengthening in bioDensity equipment (5 min, 1× week)	Usual routine	Muscle strength: chest press, leg press, core pull and vertical lift	WBV + BD: improved for chest press, leg press (vs. CON and WBV), and vertical lift (vs. CON) BD: improved for chest press, leg press (vs. WBV), and vertical lift (vs. CON)	Unreported	Adherence: 82.2% Compliance: unreported
Tseng et al., 2016 [12] Taiwan	45 community/healthy: 22(m); 23(f) WBV = 14 WBV(F) = 17 CON = 14	All: 69.2 ± 3.9 WBV: 67.2 ± 2.3 WBV(F): 71.4 ± 5.0 CON: 68.6 ± 2.5	Three months 3× week 3 × 2 min (6 min total) MC: ≈ 144 min	20 Hz; 4 mm; 3.2 g * Side-alternating	Semi-flexed knees	-	Usual routine	Muscle strength: peak torque of knee extensors and flexors (60°/s)	WBV and WBV(F): improved (vs. CON) for peak torque of knee extensors WBV(F): improved (vs. CON) for peak torque of knee flexors	Unreported	Adherence: 100% Compliance: unreported
Casimiro et al., 2015 [13] Brazil	21 community/healthy: 0(m); 21 (f) WBV + SBT = 10 SBT = 11	WBV + SBT: 77.5 ± 4.2 SBT: 74.7 ± 3.2	12 weeks 3× week 30 min MC: ≈ 1080 min	35–40 Hz; 2–4 mm; 4.9–12.9 g * Side-alternating	Postural balance exercises and lower limb strengthening	SBT: Postural balance exercises and lower limb strengthening (30 min, 3× week)	-	Muscle strength: handgrip	No difference	Unreported	Adherence: WBV + SBT (83.3%); SBT (91.7%) Compliance: unreported
Corrie et al., 2015 [14] UK	61 community/risk of falls: 24(m); 37(f) WBV(V) = 21 WBV(S) = 20 SHAM = 20	WBV(V): 81.9 ± 5.7 WBV(S): 79.5 ± 5.7 SHAM: 79.1 ± 7.8	12 weeks 3× week 2 × 30 s (1 min total)–6 × 1 min (6 min total) MC: ≈ 180 min	WBV(V): 28.4 Hz; 1.3 mm; 1.5 g; Synchronous WBV(S): 29.8 Hz; 2.9 mm; 3.6 g; Side-alternating	Semi-flexed knees	-	WBV Sham	Muscle power: 5TSTS, CMJ and power of knee extensors	WBV(V): improved (vs. SHAM) for the power of knee extensors	WVB(V) (14.3%) and WBV(S) (5%): injuries from a fall, deterioration of pre-existing arthritis, oedema and backache	Adherence: 83.6% Compliance: WBV(V): 63.4% WBV(S): 75% SHAM: 80.5%
Santin-Medeiros et al., 2015 [15] Spain	37 institutionalized/healthy: 0(m); 37(f) WBV = 19 CON = 18	All: 82.4 ± 5.7	8 months 2× week 2 × 3 min (6 min total)s MC: ≈ 420 min	20 Hz; 2 mm; 1.6 g * Synchronous	Strengthening exercises for lower limbs	-	Usual routine	Muscle strength: handgrip Muscular endurance: 30-s Sit to Stand and 30-s Arm Curl	CON: improved (vs. WBV) for 30-s Sit to Stand	Unreported	Adherence: 76% Compliance: unreported
Sitjà-Rabert et al. 2015 [16] Spain	159 institutionalized/low functionality: 52(m); 107(f) WBV + CT = 81 CT = 78	WBV + CT: 82.3 ± 7.8 CT: 82.6 ± 7.1	Six weeks 3× week 3–6 min MC: ≈ 81 min	30–35 Hz; 2–4 mm; 3.6–9.8 g * Synchronous	Strengthening exercises for lower limbs	CT: balance and strength training (30 min; 3× week)	-	Muscle power: 5TSTS	No difference	WBV: pain, itching, erythema and edema (16.3%) CT: pain, itching, erythema and edema (10%)	Adherence: WBV + CT: 82.7% CT: 82.1% Compliance: WBV + CT and CT: >75%
Álvarez-Barbosa et al., 2014 [17] Spain	29 institutionalized/healthy: 5(m); 24(f) WBV = 14 CON = 15	WBV: 84.0 ± 3.0 CON: 86.0 ± 7.5	8 weeks 3× week 12.3–17.1 min MC: ≈ 348 min	30–35 Hz; 4 mm; 7.2–9.8 g * Synchronous	Strengthening exercises for lower limbs	-	Usual nursing home care	Muscular endurance: 30-s Sit to Stand	WBV: improved (vs. CON) for 30-s Sit to Stand	No adverse event	Adherence: 73.3% (WBV); 78.6 (CON) Compliance: unreported
Leung et al., 2014 [18] China	596 community/healthy: 0(m); 596(f) WBV = 280 CON = 316	WBV: 74.5 ± 7.1 CON: 71.3 ± 7.2	18 months 5× week 20 min MC: ≈ 7800 min	35 Hz; <0.1 mm; 0.3 g Synchronous	Extended knees	-	Usual routine	Muscle strength: knee extensors	WBV: improved (vs. CON) for knee extensors	WBV: pain (2.7%), dizziness (1.4%) and hypertension (2.2%) CON: pain (3.8%), dizziness (0.3%), hypertension (4.3%) and depression (1.2%)	Adherence: 76.9% (WBV); 91.3% (CON) Compliance: 66%
Osugi et al., 2014 [19] Japan	28 ambulatory/osteoarthritis and/or spondylosis: WBV = 14 WBV + ST = 14	WBV: 72.5 ± 4.6 WBV + ST: 72.3 ± 6.5	Six months 2× week 4 min MC: ≈ 208 min	20 Hz; displacement or magnitude not reported Side-alternating	Semi-flexed knees	WBV + ST: WBV plus squat training (4 min; 2× week)	-	Muscle power: 5TSTS	WBV + ST: improved (vs. WBV) for 5TSTS	No adverse event	Adherence: 80% Compliance: 100%
Sievänen et al., 2014 [20] Finland	15 institutionalized /low functionality: 3(m); 12(f) WBV = 8 SHAM = 7	All: 84.0 ± 7.4 WBV: 84.4 ± 6.3 SHAM: 83.6 ± 8.9	10 weeks 2× week 1–5 min MC: ≈ 80 min	12–18 Hz; 2–8 mm; 0.6–5.2 g *; Side-alternating	Strengthening exercises for lower limbs	-	WBV Sham plus strengthening exercises for lower limbs	Muscle strength: handgrip	No difference	Unreported	Adherence: WBV: 87.5% SHAM: 85.7% Compliance: WBV: 74% SHAM: 73%
Zhang et al., 2014 [21] China	37 ambulatory/frail: 32(m); 5(f) WBV = 19 CON = 18	All: 85.3 ± 3.6 WBV: 85.8 ± 3.6 CON: 84.7 ± 3.7	8 weeks 3–5× week 4–5 min MC: ≈ 144 min	6–26 Hz; 1–3 mm; 0.1–4.1 g *; Side-alternating	Semi-flexed knees	-	Usual care, physical therapy and routine exercises (8 weeks)	Muscle strength: knee extensors Muscular endurance: 30-s Sit to Stand	WBV: improved (vs. CON) for knee extensors	No adverse event	Adherence: WBV: 86.4% CON: 81.8% Compliance: unreported
Calder et al., 2013 [22] New Zealand	41 institutionalized/healthy: 11(m); 30(f) WBV + PT = Unreported PT = Unreported	All: 80.1	Six weeks 3× week 4 × 75 s (5 min total) MC: ≈ 90 min	20 Hz; 2 mm; 1.6 g * Side-alternating	Semi-flexed knees	PT: physical therapy program	-	Muscle power: 5TSTS	No difference	Unreported	Adherence: 92.7% Compliance: unreported
Dudoniene et al., 2013 [23] Lithuania	40 community/healthy: 0(m); 40(f) WBV + CT = 20 CT = 20	All: 67.7 ± 4.1	8 weeks 3× week 5 × 15–30 min (2 min total) MC: ≈ 48 min	27 Hz; 3 mm; 4.4 g * Synchronous	Strengthening exercises for lower limbs	CT: strengthening, flexibility, postural control, balance and endurance exercises (50 min; 2× week)	-	Muscular endurance: 30-s Sit to Stand	No difference	Unreported	Adherence: 100% (WBV and CON) Compliance: unreported
Gómez-Cabello et al., 2013 [24] Spain	49 community/healthy: 20(m); 29(f) WBV = 24 CON = 25	All: 75.0 ± 4.7	11 weeks 3× week 10 × 45 min (7 min 30 s total) MC: ≈ 248 min	40 Hz; 2 mm; 6.4 g Synchronous	Strengthening exercises for lower limbs	-	Usual routine	Muscular endurance: 30-s Sit to Stand and 30-s Arm Curl	No difference	No adverse event	Adherence: 100% (WBV and CON) Compliance: 90.2%
Von Stengel et al., 2012 [25] Germany	141 community/healthy: 0(m); 141 (f) WBV + CT = 46 CT = 47 CON = 48	WBV + CT: 68.8 ± 3.6 CT: 68.6 ± 3.0 CON: 68.1 ± 2.7	18 months 2× week 6 min MC: ≈ 936 min	25–35 Hz; 1.7–2.0 mm; 2.1–4.9 g * Synchronous	Strengthening exercises for lower limbs	CT: aerobic dance, functional strength training coordination and balance (60 min; 2× week)	Light physical exercise and relaxation	Muscle strength: lower limb (leg press), trunk flexion and extensionMuscle power: CMJ	WBV + CT: improved (vs. CON) for lower limb, trunk flexion and extension CT: improved (vs. CON) for trunk extension	No adverse event	Adherence: WBV + CT (86%); CT (90%); CON (92%) Compliance: >75%
Marin et al., 2011 [26] Spain	34 community/healthy: (16)m; 18(f) WBV(2) = 11 WBV(4) = 12 CON = 11	All: 84.3 ± 7.4	8 weeks WBV(2): 2× week WBV(4): 4× week 4 × 30 s (2 min total)–8 × 30 s (4 min total) MC WBV(2): ≈ 52 min MC WBV(4): ≈ 104 min	35–40 Hz; 1.1–2.1 mm; 2.1–6.5 g Synchronous	Strengthening exercises for lower limbs	-	Usual routine	Muscular endurance: 30-s Sit to Stand	No difference	No adverse event	Adherence: WBV(2) (91%); WBV(4) (91%); CON (83%) Compliance: unreported
Verschueren et al., 2011 [27] Belgium	111 institutionalized/healthy: 0(m); 111(f) WBV (NS) = 28 WBV (AS) = 26 CON (NS) = 28 CON (AS) = 29	WBV (NS): 79.8 ± 5.3 WBV (AS): 80.3 ± 5.3 CON (NS): 79.6 ± 5.2 CON (AS): 78.7 ± 5.6	Six months 3× week 1 s–12 min MC: ≈ 507 min	30–40 Hz; 1.6–2.2 g Synchronous	Strengthening exercises for lower limbs	-	Usual routine	Muscle strength: knee extensors (isometric and dynamic)	No difference	No adverse event	Adherence: WBV (NS) (86%); WBV (AS) (93%); CON (NS) (93%); CON (AS) (93%) Compliance: >90%
Von Stengel et al., 2011 [28] Germany	96 community/healthy: 0(m); 96(f) WBV(V) = 34 WBV(S) = 36 CON = 36	WBV(V): 68.1 ± 4.0 WBV(S): 67.9 ± 3.8 CON: 67.6 ± 4.1	12 months 3× week 15 min MC: ≈ 540 min	WBV(V): 35 Hz; 1.7 mm; 8 g Synchronous WBV(S): 12.5 Hz; 12 mm; 8 g Side-alternating	Strengthening exercises for lower limbs	-	Light physical exercise and relaxation	Muscle strength: lower limb isometry (leg press dynamometer) Muscle power: CMJ	WBV(V) and WBV(S): improved (vs. CON) for lower limb isometry	No adverse event	Adherence: WBV(V) (94%); WBV(S) (81%); CON (92%) Compliance: WBV(V) (73%); WBV(S) (68%); CON (71%)
Machado et al., 2010 [29] Spain	26 community/healthy: 0(m); 26(f) WBV = 13 CON = 13	WBV: 79.3 ± 7.3 CON: 76.2 ± 8.4	10 weeks 3–5× week 3 × 30 min (1 min 30 s total)–8 × 1 min (8 min total) MC: ≈ 174 min	20–40 Hz; 2–4 mm; 1.6–9.8 g * Synchronous	Strengthening exercises for lower limbs	-	Usual routine	Muscle strength: lower limb (leg press) Muscle power: lower limb (leg press)	No difference	No adverse event	Adherence: WBV (87%); CON (93%) Compliance: 95%
Bogaerts et al., 2009 [30] Belgium	214 community/healthy: 114(m); 106(f) WBV = 94 CT = 60 CON = 66	All: 67.1 WBV = 66.8 CT = 66.8 CON = 67.8 (SD not reported)	12 months 3× week 4 × 30 s (2 min total)–15 × 60 s (15 min total) MC: ≈ 1248 min	35–40 Hz; 2.5–5 mm; 6.2–16.1 g * Synchronous	Strengthening exercises for upper and lower limbs	CT: cardiovascular, resistance, balance and flexibility exercises (60 min–90 min; 3× week)	Usual routine	Muscle strength: isometric strength (120°) of knee extensors	WBV and CT: improved (vs. CON) for isometric strength (120°) of knee extensors	No adverse event	Adherence: WBV (74%); CT (82%); CON (92%) Compliance: WBV (88%); CT (86%)
Furness et al., 2009 [31] Australia	73 community/healthy: 35(m); 38(f) WBV(1) = 18 WBV(2) = 18 WBV(3) = 19 CON = 18	All: 72 ± 8	Six weeks5 × 1 min (5 min total) WBV(1): 1× week; MC: ≈ 30 min WBV(2): 2× week; MC: ≈ 60 s WBV(3): 3× week; MC: ≈ 90 min	15–25 Hz; 0.5 mm; 0.45–1.26 g Side-alternating	Semi-flexed knees	-	Usual routine	Muscle power: 5TSTS	WBV(3): improved (vs. CON) for 5TSTS	Unreported	Adherence: unreported Compliance: 100%
Bogaerts et al., 2007 [32] Belgium	82 community/healthy: 82(m); 0(f) WBV = 25 CT = 25 CON = 32	WBV: 66.9 ± 0.7 CT: 67.6 ± 0.9 CON: 68.6 ± 1.0	12 months 3× week 4 × 30 s (2 min total)–15 × 60 s (15 min total) MC: ≈ 1248 min	35–40 Hz; 2.5–5 mm; 6.2–16.1 g * Synchronous	Strengthening exercises for lower limbs	CT: cardiovascular, resistance, balance, and flexibility exercises (90 min, 3× week)	Usual routine	Muscle strength: isometric strength (120°) of knee extensors Muscle power: CMJ	WBV and CT: improved (vs. CON) for isometric strength (120°) of knee extensors and CMJ	No adverse event	Adherence: WBV (81%); CT (83%); CON (89%) Compliance: WBV (88%); CT (87%)
Rees et al., 2007 [33] Australia	43 community/healthy: 23(m); 20(f) WBV + EX = 15 EX = 13 CON = 15	WBV + EX: 74.3 ± 5.0 EX: 73.1 ± 4.1 CON: 73.1 ± 4.6	8 weeks 3× week 6 × 45 s (4 min 30 s total)–6 × 80 s (8 min total) MC: ≈ 150 min	26 Hz; 5–8 mm; 6.8–10.9 g Side-alternating	Strengthening exercises for lower limbs	EX: Strengthening exercises for lower limbs–6 × 45 s (4 min 30 s total)–6 × 80 s (8 min total), 3× week	Unreported	Muscle strength: peak torque of knee, hip (60°/s) and ankle (30°/s) extensors and flexors Muscle power: 5TSTS	WBV + EX and EX: improved (vs. CON) for peak torque of knee extension and 5TSTS WBV + EX: improved (vs. EX and CON) for peak torque of ankle plantar-flexor	Unreported	Adherence: WBV + EX and EX (100%); EX (87%); CON (100%) Compliance: WBV + EX and EX (99%)
Bautmans et al., 2005 [34] Belgium	24 institutionalized/low functionality: 9(m); 15(f) WBV = 13 SHAM = 11	All: 77.5 ± 11.0 WBV: 76.6 ± 11.8 SHAM: 78.6 ± 10.4	Six weeks 3× week 2 × 30 s (1 min total)–4 × 1 min (4 min total) MC: ≈ 36 min	35–40 Hz; 2–5 mm; 4.9–16.1 g Synchronous	Strengthening exercises for lower limbs	-	WBV Sham: Strengthening exercises for lower limbs	Muscle strength: handgrip, leg extension Muscular endurance: 30-s Sit to Stand	No difference	WBV: groin pain (8%) and airway infection (8%)	Adherence: WBV (77%); SHAM (100%) Compliance: WBV (96%); SHAM (86%)
Runge et al., 2000 [35] Germany	34 community/healthy: 23(m); 11(f) WBV = 17 CON = 17	All: 67 (61–85)	Two months 3× week 5 min MC: ≈ 144 min	27 Hz; 7–14 mm; 10.3–20.5 g * Side-alternating	Semi-flexed knees	-	Unreported	Muscle power: 5TSTS	No difference	WBV: inflammation in the forefoot (6%)	Adherence: 87.2% Compliance: unreported

Abbreviations: m (male); f (female); min (minutes); s (seconds); WBV (whole-body vibration); NWBV: normoxic whole-body vibration; HWBV: hypoxic whole-body vibration; WBV(I): intensity whole-body vibration; WBV(E): exposure time whole-body vibration; WBV(L): low-frequency whole-body vibration; WBV(M): Medium-frequency whole-body vibration; WBV(H): High-frequency whole-body vibration; WBV(F): whole-body vibration without visual feedback; WBV(AS): whole-body vibration with additional supplementation; WBV(NS): whole-body vibration with normal supplementation; CON(AS): control group and additional supplementation; CON(NS): control group and normal supplementation; WBV(2): whole-body vibration 2 days per week; WBV(4): whole-body vibration 4 days per week; RT (resistance training); MT: mental training; WBV + P: whole-body vibration and creatine placebo; WBV + C: whole-body vibration and creatine; RT: resistance training; CT: combined training; ST: squat training; QG (qi gong); SO (spinal orthosis); WBV(V): vertical (synchronous) whole-body vibration; WBV(S): side-alternating whole-body vibration; TC: Tai Chi; BD: bioDensity Training; SBT: Strength and balance training; PT: physical therapy; EX: strengthening exercises for lower limbs; CON: control; SHAM: simulated whole-body vibration; 5TSTS (five-times-sit-to-stand test); CMJ: countermovement jump; * Calculated based on the magnitude of frequency and peak-to-peak displacement [45]. ‡ Adherence: percentage of participants who remained until the end of the intervention period; Compliance: percentage of participation/attendance during the intervention period.

**Table 2:** The methodological quality of the studies included in the systematic review, as evaluated by the PEDro scale.

Author	Eligibility Criteria	Random Allocation	Concealed Allocation	Baseline Comparability	Blind Subjects	Blind Therapists	Blind Assessor	Follow-Up Dropout <15%	Intention-to-Treat Analysis	Between-Group Comparisons	Point Estimates and Variability	Score
Genest et al., 2021 [1]	No	Yes	No	Yes	No	No	No	Yes	No	Yes	Yes	5
Camacho-Cardenosa et al., 2019 [2]	Yes	Yes	No	No	No	No	Yes	No	No	Yes	Yes	4
Zhu et al., 2019 [3]	Yes	Yes	No	Yes	No	No	No	Yes	No	Yes	Yes	5
Lam et al., 2018 [4]	Yes	Yes	No	Yes	No	No	Yes	Yes	Yes	Yes	Yes	7
Pessoa et al., 2018 [5]	No	Yes	Yes	Yes	No	No	Yes	Yes	No	Yes	Yes	7
Goudarzian et al., 2017 [6]	Yes	Yes	No	Yes	No	No	No	Yes	No	Yes	Yes	5
Goudarzian et al., 2017 [7]	Yes	Yes	No	Yes	No	No	Yes	No	No	Yes	Yes	5
Han et al., 2017 [8]	No	Yes	No	No	No	No	No	No	No	Yes	Yes	3
Wei et al., 2017 [9,10]	Yes	Yes	No	Yes	No	No	No	Yes	Yes	Yes	Yes	6
Smith et al., 2016 [11]	Yes	Yes	No	Yes	No	No	No	Yes	No	Yes	Yes	5
Tseng et al., 2016 [12]	Yes	Yes	No	Yes	No	No	No	Yes	No	Yes	Yes	5
Casimiro et al., 2015 [13]	Yes	Yes	No	Yes	No	No	Yes	Yes	No	Yes	Yes	6
Corrie et al., 2015 [14]	Yes	Yes	No	Yes	Yes	No	Yes	Yes	Yes	Yes	Yes	8
Santin-Medeiros et al., 2015 [15]	Yes	Yes	No	No	No	No	No	Yes	No	Yes	Yes	4
Sitja-Rabert et al., 2015 [16]	Yes	Yes	Yes	Yes	No	No	Yes	No	Yes	Yes	Yes	7
Álvarez-Barbosa et al., 2014 [17]	Yes	Yes	No	Yes	No	No	No	No	Yes	Yes	Yes	5
Leung et al., 2014 [18]	Yes	Yes	Yes	Yes	No	No	Yes	Yes	Yes	Yes	Yes	8
Osugi et al., 2014 [19]	Yes	Yes	No	Yes	No	No	No	No	No	Yes	Yes	4
Sievänen et al., 2014 [20]	Yes	No	Yes	Yes	No	No	Yes	Yes	Yes	Yes	Yes	7
Zhang et al., 2014 [21]	Yes	Yes	Yes	Yes	No	No	Yes	No	Yes	Yes	Yes	7
Calder et al., 2013 [22]	No	Yes	No	No	No	No	Yes	Yes	No	Yes	Yes	5
Dudoniene et al., 2013 [23]	Yes	Yes	No	Yes	No	No	No	Yes	Yes	Yes	Yes	6
Gómez-Cabello et el., 2013 [24]	Yes	Yes	No	Yes	No	No	No	Yes	No	Yes	Yes	5
Von Stengel et al., 2012 [25]	Yes	Yes	No	Yes	No	No	Yes	Yes	Yes	Yes	Yes	7
Marin et al., 2011 [26]	No	Yes	No	Yes	No	No	No	Yes	No	Yes	Yes	5
Verschueren et al. 2011 [27]	Yes	Yes	No	Yes	No	No	Yes	Yes	Yes	Yes	Yes	7
Von Stengel et al., 2011 [28]	Yes	Yes	No	Yes	No	No	Yes	Yes	No	Yes	Yes	6
Machado et al., 2010 [29]	Yes	Yes	No	No	No	No	Yes	Yes	No	Yes	Yes	5
Bogaerts et al., 2009 [30]	Yes	Yes	No	Yes	No	No	No	Yes	Yes	Yes	Yes	6
Furness et al., 2009 [31]	Yes	Yes	No	Yes	No	No	No	Yes	No	Yes	Yes	5
Bogaerts et al., 2007 [32]	No	Yes	No	Yes	No	No	No	No	No	Yes	Yes	4
Rees et al., 2007 [33]	No	Yes	No	Yes	No	No	No	Yes	No	Yes	Yes	5
Bautmans et al., 2005 [34]	Yes	Yes	No	Yes	Yes	No	Yes	Yes	No	Yes	Yes	7
Runge et al., 2000 [35]	Yes	Yes	No	No	No	No	No	Yes	No	No	No	2

**Table 3:** The primary analysis of the effectiveness of WBV vs. control groups on muscle strength, power and endurance.

Measurements	Std. Mean Difference	95% CI		*n*	Studies	I^2^	*p*
		Lower	Upper				
Muscle Strength							
Knee extensors	0.53	0.32	0.74	937	9	30%	<0.00001
Knee flexors	0.64	0.31	0.96	161	4	0%	0.0002
Leg extensors	0.68	0.17	1.20	209	3	62%	0.009
Ankle plantar-flexors	0.65	0.12	1.18	61	2	0%	0.02
Ankle dorciflexors	0.31	−0.43	1.05	85	2	63%	0.41
Hip flexors	0.17	−0.26	0.59	85	2	0%	0.44
Handgrip	0.13	−0.31	0.57	184	7	50%	0.55
Muscle Power							
Five-times-sit-to-stand	−0.31	−0.63	0.02	386	6	50%	0.07
Countermovement jump	0.17	−0.12	0.47	189	2	0%	0.24
Muscle endurance							
30-s sit-to-stand	0.10	−0.19	0.39	184	5	42%	0.51
30-s arm curl	0.01	−0.87	0.90	86	2	76%	0.97

95% CI: Confidence interval at 95%.

## Data Availability

The data presented in this study are available in this article and supplemental materials.

## Supplementary Materials

The following supporting information can be downloaded at: https://www.mdpi.com/article/10.3390/jcm12134467/s1, Table S1: Records that a full text was not found; Table S2: Reports excluded in the full text reading phase; Figure S1: Primary analysis comparing the effectiveness of WBV vs. control groups for muscle strength: (a) ankle dorsiflexors; (b) hip flexors; (c) hand grip; Figure S2: Primary analysis comparing the effectiveness of WBV vs. control groups for muscle power: (a) five-times-sit-to-stand test; (b) countermovement jump; Figure S3: Primary analysis comparing the effectiveness of WBV vs. control groups for muscular endurance: (a) 30-second sit to stand; (b) 30-second arm curl; Figure S4: Sensitivity analysis comparing the effectiveness of WBV vs. control groups for: (a) knee extensor muscle strength; (b) muscular strength of the extensors of the lower limbs; (c) handgrip strength; (d) five-times-sit-to-stand; (e) countermovement jump; (f) 30-second sit-to-stand test; Figure S5: Analysis comparing the effectiveness of WBV vs. other forms of exercise for muscle strength: (a) knee extensors; (b) knee flexors; (c) ankle dorsiflexors; (d) hip flexors; (e) hand grip; Figure S6: Analysis comparing the effectiveness of WBV with participants maintaining a static position vs. control groups: (a) muscle strength of the knee extensors; (b) muscle strength of the knee flexors; (c) muscular strength of the ankle dorsiflexors; (d) muscle strength of the hip flexors; (e) handgrip muscle strength; (f) five-times-sit-to-stand; (g) 30-s sit-to-stand; Figure S7: Analysis comparing the effectiveness of WBV with participants performing muscle-strengthening exercises vs. control groups: (a) muscle strength of the knee extensors; (b) muscle strength of the knee flexors; (c) muscular strength of the extensors of the lower limbs; (d) muscular strength of the plantar flexors; (e) muscular strength of the ankle dorsiflexors; (f) muscle strength of the hip flexors; (g) handgrip muscle strength; (h) five-times-sit-to-stand; (i) countermovement jump; (j) 30-s sit-to-stand; (k) 30-s arm curl; Figure S8: Analysis comparing the effectiveness of WBV vs. control groups on synchronous type platforms: (a) knee extensor muscle strength; (b) muscular strength of the extensors of the lower limbs; (c) muscular strength of the plantar flexors; (d) muscle strength of the hip flexors; (e) handgrip muscle strength; (f) five-times-sit-to-stand; (g) countermovement jump; (h) 30-s sit-to-stand; (i) 30-s arm curl; Figure S9: Analysis comparing the effectiveness of WBV vs. control groups on side-alternating platforms: (a) muscle strength of the knee extensors; (b) muscle strength of the knee flexors; (c) muscular strength of the extensors of the lower limbs; (d) muscular strength of ankle plantar flexors; (e) muscular strength of the ankle dorsiflexors; (f) muscle strength of the hip flexors; (g) handgrip muscle strength; (h) five-times-sit-to-stand; (i) countermovement jump; (j) 30-s sit-to-stand; Figure S10: Analysis comparing the effectiveness of WBV administered at a low cumulative dose (≤ 144 minutes) vs. control groups: (a) muscle strength of the knee extensors; (b) muscle strength of the knee flexors; (c) muscular strength of the extensors of the lower limbs; (d) muscular strength of the plantar flexors; (e) handgrip muscle strength; (f) five-times-sit-to-stand; (g) 30-s sit-to-stand; (h) 30-s arm curl; Figure S11: Analysis comparing the effectiveness of WBV administered at a high cumulative dose (> 144 minutes) vs. control groups: (a) muscle strength of the knee extensors; (b) muscle strength of the knee flexors; (c) muscular strength of the extensors of the lower limbs; (d) muscular strength of the plantar flexors; (e) muscular strength of the ankle dorsiflexors; (f) muscle strength of the hip flexors; g) handgrip muscle strength; (h) five-times-sit-to-stand; (i) countermovement jump; (j) 30-s sit-to-stand; (k) 30-s arm curl; Figure S12: Analysis comparing the effectiveness of WBV administered at low magnitude (≤4.4 g) vs. control groups: (a) muscle strength of the knee extensors; (b) muscle strength of the knee flexors; (c) muscular strength of the extensors of the lower limbs; (d) handgrip muscle strength; (e) five-times-sit-to-stand; (f) countermovement jump; (g) 30-s sit-to-stand; (h) 30-s arm curl; Figure S13: Analysis comparing the effectiveness of WBV administered at high magnitude (> 4.4 g) vs. control groups: (a) muscle strength of the knee extensors; (b) muscle strength of the knee flexors; (c) muscular strength of the extensors of the lower limbs; (d) muscular strength of the plantar flexors; (e) muscular strength of the ankle dorsiflexors; (f) muscle strength of the hip flexors; g) handgrip muscle strength; (h) Five-times-sit-to-stand; (i) countermovement jump; (j) 30-s sit-to-stand; (k) 30-s arm curl. S1: Search strategy in each database.
