# Supplementary material for: Impacts of Whole-Body Vibration on Muscle Strength, Power, and Endurance in Older Adults: A Systematic Review and Meta-Analysis
Source: J Clin Med. 2023 Jul 3;12(13):4467. doi: 10.3390/jcm12134467 (PMC10342949; doi:10.3390/jcm12134467)
Supplement: Supplementary file 1 [file jcm-12-04467-s001.zip › jcm-2428041-supplementary.pdf]

Supplementary S1

Search strategy in each database
Note: To increase search sensitivity we left "all fields" in each search term.

PubMed search strategy (pubmed.gov, 1996 to 1, May 2022).
#1: whole body vibration
#2: WBV
#3: vibration
#4: vibration therapy
#5: vibration training
#6: oscillating platforms
#7: vibrating platform
#8: vibration device
#9: mechanical vibration
#10: #1 OR #2 OR #3 OR #4 OR #5 OR #6 OR #7 OR #8 OR #9
#11: aged [MeSH Terms]
#12: aging [MeSH Terms]
#13: ageing
#14: elderly
#15: older people
#16: older adults
#17: older adult
#18: older women
#19: older men
#20: geriatric
#21: geriatrics
#22: frail elderly [MeSH Terms]
#23: elder
#24: elders
#25: aged people
#26: #11 OR #12 OR #13 OR #14 OR #15 OR #16 OR #17 OR #18 OR #19 OR #20 OR 21 OR #22 OR #23 OR #24 OR #25
#27: muscle strength [MeSH Terms]
#28: muscle strength dynamometer [MeSH Terms]
#29: strengthening
#30: strength
#31: torque [MeSH Terms]
#32: maximal voluntary contraction
#33: 1RM
#34: one repetition maximum
#35: 1 repetition maximum
#36: muscular endurance
#37: muscle endurance
#38: isometric
#39: isometry
#40: muscle power
#41: power
#42: muscular power
#43: power output

#44: functional tests
#45: functional autonomy
#46: functional mobility
#47: #27 OR #28 OR #29 OR #30 OR #31 OR #32 OR #33 OR #34 OR #35 OR #36 OR #37 OR #38 OR #39 OR #40 OR #41 OR #42 OR #43 OR #44 OR #45 OR #46
#48: #10 AND #26 AND #47

Embase (embase.com, 1947 to 1, May 2022)
#1: whole body vibration/exp
#2: WBV
#3: vibration/exp
#4: vibration therapy/exp
#5: vibration training
#6: oscillating platforms
#7: vibrating platform
#8: vibration device
#9: mechanical vibration/exp
#10: #1 OR #2 OR #3 OR #4 OR #5 OR #6 OR #7 OR #8 OR #9
#11: aged/exp
#12: aging/exp
#13: ageing/exp
#14: elderly/exp
#15: older people/exp
#16: older adults/exp
#17: older adult/exp
#18: older women
#19: older men
#20: geriatric/exp
#21: geriatrics/exp
#22: frail elderly/exp
#23: elder
#24: elders
#25: aged people/exp
#26: #11 OR #12 OR #13 OR #14 OR #15 OR #16 OR #17 OR #18 OR #19 OR #20 OR 21 OR #22 OR #23 OR #24 OR #25
#27: muscle strength/exp
#28: muscle strength dynamometer/exp
#29: strengthening
#30: strength/exp
#31: torque/exp
#32: maximal voluntary contraction/exp
#33: 1RM
#34: one repetition maximum/exp
#35: 1 repetition maximum
#36: muscular endurance/exp
#37: muscle endurance/exp
#38: isometric
#39: isometry
#40: muscle power/exp
#41: power/exp
#42: muscular power/exp

#43: power output/exp
#44: functional tests
#45: functional autonomy
#46: functional mobility/exp
#47: #27 OR #28 OR #29 OR #30 OR #31 OR #32 OR #33 OR #34 OR #35 OR #36 OR #37 OR #38 OR #39 OR #40 OR #41 OR #42 OR #43 OR #44 OR #45 OR #46
#48: #10 AND #26 AND #47

CENTRAL (cochranelibrary.com, 1998 to 1, May 2022)
#1: whole body vibration
#2: WBV
#3: vibration
#4: vibration therapy
#5: vibration training
#6: oscillating platforms
#7: vibrating platform
#8: vibration device
#9: mechanical vibration
#10: #1 OR #2 OR #3 OR #4 OR #5 OR #6 OR #7 OR #8 OR #9
#11: aged
#12: aging
#13: ageing
#14: elderly
#15: older people
#16: older adults
#17: older adult
#18: older women
#19: older men
#20: geriatric
#21: geriatrics
#22: frail elderly
#23: elder
#24: elders
#25: aged people
#26: #11 OR #12 OR #13 OR #14 OR #15 OR #16 OR #17 OR #18 OR #19 OR #20 OR 21 OR #22 OR #23 OR #24 OR #25
#27: muscle strength
#28: muscle strength dynamometer
#29: strengthening
#30: strength
#31: torque
#32: maximal voluntary contraction
#33: 1RM
#34: one repetition maximum
#35: 1 repetition maximum
#36: muscular endurance
#37: muscle endurance
#38: isometric
#39: isometry
#40: muscle power
#41: power

#42: muscular power
#43: power output
#44: functional tests
#45: functional autonomy
#46: functional mobility
#47: #27 OR #28 OR #29 OR #30 OR #31 OR #32 OR #33 OR #34 OR #35 OR #36 OR #37 OR #38 OR #39 OR #40 OR #41 OR #42 OR #43 OR #44 OR #45 OR #46
#48: #10 AND #26 AND #47 in Trials

CINAHL (via EBSCOhost, 1937 to 1, May 2022)
S1: whole body vibration
S2: WBV
S3: vibration
S4: vibration therapy
S5: vibration training
S6: oscillating platforms
S7: vibrating platform
S8: vibration device
S9: mechanical vibration
S10: S1 OR S2 OR S3 OR S4 OR S5 OR S6 OR S7 OR S8 OR S9
S11: aged
S12: aging
S13: ageing
S14: elderly
S15: older people
S16: older adults
S17: older adult
S18: older women
S19: older men
S20: geriatric
S21: geriatrics
S22: frail elderly
S23: elder
S24: elders
S25: aged people
S26: S11 OR S12 OR S13 OR S14 OR S15 OR S16 OR S17 OR S18 OR S19 OR S20 OR 21 OR S22 OR S23 OR S24 OR S25
S27: muscle strength
S28: muscle strength dynamometer
S29: strengthening
S30: strength
S31: torque
S32: maximal voluntary contraction
S33: 1RM
S34: one repetition maximum
S35: 1 repetition maximum
S36: muscular endurance
S37: muscle endurance
S38: isometric
S39: isometry
S40: muscle power

S41: power
S42: muscular power
S43: power output
S44: functional tests
S45: functional autonomy
S46: functional mobility
S47: S27 OR S28 OR S29 OR S30 OR S31 OR S32 OR S33 OR S34 OR S35 OR S36 OR S37 OR S38 OR S39 OR S40 OR S41 OR S42 OR S43 OR S44 OR S45 OR S46
S48: S10 AND S26 AND S47

Web of Science (webofscience.com, 1900 to 1, May 2022)
1: whole body vibration
2: WBV
3: vibration
4: vibration therapy
5: vibration training
6: oscillating platforms
7: vibrating platform
8: vibration device
9: mechanical vibration
10: #1 OR #2 OR #3 OR #4 OR #5 OR #6 OR #7 OR #8 OR #9
11: aged
12: aging
13: ageing
14: elderly
15: older people
16: older adults
17: older adult
18: older women
19: older men
20: geriatric
21: geriatrics
22: frail elderly
23: elder
24: elders
25: aged people
26: #11 OR #12 OR #13 OR #14 OR #15 OR #16 OR #17 OR #18 OR #19 OR #20 OR 21 OR #22 OR #23 OR #24 OR #25
27: muscle strength
28: muscle strength dynamometer
29: strengthening
30: strength
31: torque
32: maximal voluntary contraction
33: 1RM
34: one repetition maximum
35: 1 repetition maximum
36: muscular endurance
37: muscle endurance
38: isometric
39: isometry

40: muscle power
41: power
42: muscular power
43: power output
44: functional tests
45: functional autonomy
46: functional mobility
47: #27 OR #28 OR #29 OR #30 OR #31 OR #32 OR #33 OR #34 OR #35 OR #36 OR #37 OR #38 OR #39 OR #40 OR #41 OR #42 OR #43 OR #44 OR #45 OR #46
48: #10 AND #26 AND #47

SPORTDiscus (via EBSCOhost, 1985 to 1, May 2022)
S1: whole body vibration
S2: WBV
S3: vibration
S4: vibration therapy
S5: vibration training
S6: oscillating platforms
S7: vibrating platform
S8: vibration device
S9: mechanical vibration
S10: S1 OR S2 OR S3 OR S4 OR S5 OR S6 OR S7 OR S8 OR S9
S11: aged
S12: aging
S13: ageing
S14: elderly
S15: older people
S16: older adults
S17: older adult
S18: older women
S19: older men
S20: geriatric
S21: geriatrics
S22: frail elderly
S23: elder
S24: elders
S25: aged people
S26: S11 OR S12 OR S13 OR S14 OR S15 OR S16 OR S17 OR S18 OR S19 OR S20 OR 21 OR S22 OR S23 OR S24 OR S25
S27: muscle strength
S28: muscle strength dynamometer
S29: strengthening
S30: strength
S31: torque
S32: maximal voluntary contraction
S33: 1RM
S34: one repetition maximum
S35: 1 repetition maximum
S36: muscular endurance
S37: muscle endurance
S38: isometric

S39: isometry
S40: muscle power
S41: power
S42: muscular power
S43: power output
S44: functional tests
S45: functional autonomy
S46: functional mobility
S47: S27 OR S28 OR S29 OR S30 OR S31 OR S32 OR S33 OR S34 OR S35 OR S36 OR S37 OR S38 OR S39 OR S40 OR S41 OR S42 OR S43 OR S44 OR S45 OR S46
S48: S10 AND S26 AND S47

LILACS (lilacs.bvsalud.org, 1986 to 1, May 2022)
Title, Abstract and Subject: (“aged” OR “aging” OR “ageing” OR "elderly" OR "older people" OR "older adults" OR "older adult" OR "older women" OR "older men" OR “geriatric” OR “geriatrics” OR “frail elderly” OR “elder” OR “elders” OR “aged people”) AND (“whole body vibration” OR “WBV” OR “vibration” OR “vibration therapy” OR “vibration training” OR “oscillating platforms” OR “vibrating platform” OR “vibration device” OR “mechanical vibration”) AND (“muscle strength” OR “muscle strength dynamometer” OR “strengthening” OR “strength” OR “torque ” OR “maximal voluntary contraction” OR “1RM” OR “one repetition maximum” OR “1 repetition maximum” OR “muscular endurance” OR “muscle endurance” OR “isometric” OR “isometry” OR “muscle power” OR “power” OR “muscular power” OR “power output” OR "functional tests" OR "functional autonomy" OR "functional mobility")

PEDro (pedro.org.au, 2000 to 1, May 2022)
Abstract and Title: whole body vibration AND older*

ClinicalTrials.gov and **WHO ICTRP** search strategy
Last searched 1, May 2022
Basic search: whole body vibration AND older

Supplementary Table S1. Records that a full text was not found.

Author	Title	Journal, volume and page / or web site	Reason*
Rogan et al., 2011	Acute effects of stochastic resonance whole body vibration on chair rising performance in the elderly: a pilot study	Physiotherapy (United Kingdom). 97:eS1053-eS1054	1
Genest et al., 2017	Combined efficacy of different exercise interventions in osteosarcopenic men	Journal of Bone and Mineral Research. 32:S398	1
Li et al., 2014	Effects of different interventions on falls and falls-related functional factors in veteran elderly: a pilot randomized controlled trial	Annals of Physical and Rehabilitation Medicine. 57:e153	1
Borges et al., 2015	Effects of whole-body Vibration Exercises on Muscle Strength, Balance and Gait Performance in Elderly Women	Archives of Physical Medicine & Rehabilitation. 96:e17-e17	1
Von Stengel et al., 2010	Effects of Whole Body Vibration on vertical versus rotational devices on osteoporotic risk factors - Preliminary results of the ELVIS II study	Osteoporosis International. 21:S380-S381	1
Wu et al., 2011	Effects of Whole Body Vibration Training on Functional Fitness in Female Elderly	Medicine & Science in Sports & Exercise. 43:519	1
Lee et al., 2019	Effects of whole body vibration training using side-alternating vibration platform with tilt table in hospitalized older adults with sarcopenia: a randomized controlled pilot study	Aging Medicine and Healthcare. 10:35	1
Seo et al., 2019	Effects of whole body vibration training using side-alternating vibration platform with tilt table in hospitalized older adults with sarcopenia: a randomized controlled pilot study	Age and Ageing. 48:iv18	1
Sievänen et al., 2012	Effect of whole body vibration training on physical performance among institutionalized older people: a 10-WK pilot blinded randomized controlled trial	Osteoporosis International. 23:S83-S84	1

De Souza et al., 2019	Efficacy of the association of inspiratory muscle training with whole body vibration on respiratory muscle strength, functionality, balance and physical performance in prefrail older women: a randomized double-blind clinical trial	European Respiratory Journal. 54:PA724	1
Rogan et al., 2015	Feasibility study evaluating the effects of a four weeks stochastic resonance whole-body vibration intervention on functional performance in frail elderly	Physiotherapy (United Kingdom). 101:eS1250	1
Li et al., 2013	Low-magnitude, high-frequency vibration treatment reduced fall incidences and fracture risks in community elderly: a prospective cluster- randomized controlled trial and 1 year follow-up	Osteoporosis International. 24: S529	1
Rosado et al., 2021	Novel approaches to reduce the risk of falling in community dwellings: effects of two multimodal programs in lower-body strength—a pilot study	European Journal of Public Health. 31:1	1
Zhu et al., 2016	Tai chi and whole-body vibration therapy in the elderly: a randomized controlled trial	Journal of the American Geriatrics Society. 64:S373	1
Rogan et al., 2011	The effects of a four-week stochastic resonance whole body vibration training on chair rising performance in the elderly: pilot study	Physiotherapy (United Kingdom). 97:eS1053	1
Bogaerts et al., 2008	The effects of long term Whole Body Vibration training in older individuals	Isokinetics & Exercise Science. 16:179-180	1
Lark et al., 2017	Time lag between perceived decreased confidence and actual physical function following 16 weeks vibration training in frail elderly	New Zealand Journal of Sports Medicine. 44:35	1
Wei et al., 2015	Whole-body vibration training with medium frequency improves muscle performance in people with Sarcopenia	Physiotherapy (United Kingdom). 101: eS1620-eS1621	1

Rogan, 2012	Application of Whole-body Vibration With Stochastic Resonance in Frail Elderly: The Effects on Postural Control	https://clinicaltrials.gov/show/NCT01543243	2
Borja, 2013	Cost-utility and cost-effectiveness of a 8-wk whole-body vibration (WBV)-based intervention to improve balance and mobility among older adults with reduced mobility: a randomized controlled trial	https://trialsearch.who.int/Trial2.aspx?TrialID=ACTRN12613000189729	2
Souza, 2018	Effects of WBV Associated With IMT on Inflammatory Markers, Body Composition, Muscle Strength and Thickness	https://clinicaltrials.gov/show/NCT03689322	2
Zhang., 2014	Effects of Whole-body Vibration in the Frail Elderly	https://clinicaltrials.gov/show/NCT02090192	2
Von Stengel, 2008	Effects of Whole Body Vibration on Postmenopausal Risk-factors in Elderly Women	https://clinicaltrials.gov/show/NCT00667667	2
Deslandes, 2012	Effect of Strength Training and Whole Body Vibration in Healthy Elderly	https://clinicaltrials.gov/show/NCT01526109	2
Kalender, 2006	Effect of Whole Body Vibration on Bone and Fall Related Parameters	https://clinicaltrials.gov/show/NCT00292916	2
Araújo, 2020	Efficacy of a multimodal exercise training associated with whole body vibration at falls risk and quality of life in older adults	https://trialsearch.who.int/Trial2.aspx?TrialID=RBR-3xdf4k	2
Araújo, 2018	Immediate Effect Of Whole-Body Vibration And Walk in Osteoporosis/Osteopenia	https://clinicaltrials.gov/show/NCT03448276	2
Lim, 2018	Impact of Whole-body Vibration Training on Sarcopenic Elderly	https://clinicaltrials.gov/show/NCT03695354	2
Wei, 2017	The effects and mechanism of whole body vibration training on muscle performance, physical performance and balance in the older people with age-related loss of muscle mass	https://trialsearch.who.int/Trial2.aspx?TrialID=ISRCTN63583948	2
Manimmanakorn, 2018	The effects of weighted vest and whole body vibration on body balance in the elderly	https://trialsearch.who.int/Trial2.aspx?TrialID=TCTR20190306001	2

Lai, 2016	The Effectiveness of Whole-Body Vibration With Hot Pack on Muscle strength, Balance Performance, and Flexibility in the Elderly	https://trialsearch.who.int/ Trial2.aspx?TrialID=ChiCTR-IOR-16008059	2
Verschueren, 2011	The Effect of 6 Months of Local Vibration Training in Institutionalized Elderly	https://clinicaltrials.gov/ show/NCT01499186	2
Jounaghani, 2014	Vibration and Creatine Effects on Physical Fitness	https://trialsearch.who.int/ Trial2.aspx?TrialID=IRCT2014061517743N2	2
Machado, 2016	Whole Body Vibrations on Functional Capacity, Muscular Strength, and Biochemical Profile in Elders	https://clinicaltrials.gov/ ct2/show/study/NCT03030456	2
Zhang et al., 2013	Effects of Whole-Body Vibration Exercise on Lower-Extremity Muscle Strength and Power of the Elderly	Journal of Shenyang Sport University. 32:79-81	3
Li et al., 2018	Effects of whole body vibration training at different frequencies on the balance, lower limb muscle strength and position sense of elderly women	Journal of Physical Education / Tiyu Xuekan. 25:128-134	3
Amaral et al., 2014	Whole-body vibration training does not modify anthropometric parameters and lower limb strength in elderly people	Clinical and Experimental Medical Letters. 55:6-10	3
McClain, 2014	The effect of whole body vibration training on balance and strength of the lower extremity muscles in older individuals	These. Doctor in Physical Therapy, Florida Gulf Coast University	3

* Reasons: 1 – Abstract published in Conference; 2 – Clinical trial registration; 3 – Full report not found.

Supplementary Table S2. Reports excluded in the full text reading phase.

Author and year	Title	Journal, volume and page	Reason*
Grubbs et al. (2020)	Whole-body Vibration Training in Frail, Skilled Nursing Home Residents	International Journal of Exercise Science. 13:140-156	1
He et al. (2020)	The Genetic Effect on Muscular Changes in an Older Population: A Follow-Up Study after One-Year Cessation of Structured Training	Genes. 11:968	1
Cheung et al. (2016)	Improvement in muscle performance after one-year cessation of low-magnitude high-frequency vibration in community elderly	Journal of Musculoskeletal & Neuronal Interactions. 16:4-11	1
Lark et al. (2015)	Physiological, psychological and functional changes with whole body vibration exercise in the elderly: FEVER methodology and protocols	Contemporary Clinical Trials. 44:129-133	1
Kennis et al. (2013)	Effects of Fitness and Vibration Training on Muscle Quality: A 1-Year Postintervention Follow-Up in Older Men	Archives of Physical Medicine and Rehabilitation. 94:910-918	1
Cristi et al. (2014)	Whole-body vibration training increases physical fitness measures without alteration of inflammatory markers in older adults	European Journal of Sport Science. 14:611-619	1
Carr et al. (2012)	Whole-body Vibration Training in Older Adults: Retention of the Strengthening Effects	Critical Reviews in Physical and Rehabilitation Medicine. 24:51-67	1
Sitjà-Rabert et al. (2011)	Whole body vibration for older persons: an open randomized, multicentre, parallel, clinical trial	BMC Geriatrics. 11:89	1
Bemben et al. (2010)	Effects of combined whole-body vibration and resistance training on muscular strength and bone metabolism in postmenopausal women	Bone. 47:650-656	1
Silva et al. (2010)	Efeito do treinamento vibratório na força muscular e em testes funcionais em idosos fisicamente ativos	Revista Brasileira de Cineantropometria e Desempenho Humano. 11:166-173	1
Lin and Woollacott (2005)	Association between sensorimotor function and functional and reactive balance control in the elderly	Age and Ageing. 34:358-363	1
Tsuji et al. (2012)	Effects of static acceleration training with a whole-body vibration machine in community-dwelling older adults	Japanese Journal of Physical Fitness and Sports Medicine. 61:2011-219	1
Kessler et al. (2014)	Effect of stochastic resonance whole body vibration on functional performance in the frail elderly: A pilot study	Archives of Gerontology and Geriatrics. 59:305-311	2
Rogan et al. (2015)	Preliminary inconclusive results of a randomised double blinded cross-over pilot trial in long-term-care dwelling elderly assessing the feasibility of stochastic resonance whole-body vibration	European Review of Aging and Physical Activity. 12:5	2
Tankisheva et al. (2015)	Effects of a Six-Month Local Vibration Training on Bone Density, Muscle Strength, Muscle Mass, and Physical Performance in Postmenopausal Women	Journal of Strength and Conditioning Research. 29:2613-2622	2
Bellomo et al. (2013)	Muscle Strength and Balance Training in Sarcopenic Elderly: A Pilot Study with Randomized Controlled Trial	European Journal of Inflammation. 11:193-201	2
Wadsworth and Lark (2020)	Effects of Whole-Body Vibration Training on the Physical Function of the Frail Elderly: An Open, Randomized Controlled Trial	Archives of Physical Medicine and Rehabilitation. 101, 1111-1119	3

Saedmocheshi et al. (2014)	Effect of whole body vibration on muscle performance in elderly men	Medical Journal of Tabriz University of Medical Sciences and Health Services. 36:34-39	3
Pollock et al. (2012)	Whole-body vibration in addition to strength and balance exercise for falls-related functional mobility of frail older adults: a single-blind randomized controlled trial	Clinical Rehabilitation. 26:915-923	3
Rogan et al. (2012)	Stochastic resonance whole-body vibration training for chair rising performance on untrained elderly: A pilot study	Archives of Gerontology and Geriatrics. 55:468-473	3
Von Stengel et al. (2011)	Effects of whole body vibration on bone mineral density and falls: results of the randomized controlled ELVIS study with postmenopausal women	Osteoporosis International. 22:317-325	3
Gloeckl et al. (2017)	What's the secret behind the benefits of whole-body vibration training in patients with COPD? A randomized, controlled trial	Respiratory Medicine. 126:17-24	4
Perchthaler et al. (2015)	Evaluation of a Six-Week Whole-Body Vibration Intervention on Neuromuscular Performance in Older Adults	Journal of Strength and Conditioning Research. 29:86-95	4
Roelants et al. (2004)	Whole-Body-Vibration Training Increases Knee-Extension Strength and Speed of Movement in Older Women	Journal of the American Geriatrics Society. 52:901-908	4
Verschueren et al. (2004)	Effect of 6-Month Whole Body Vibration Training on Hip Density, Muscle Strength, and Postural Control in Postmenopausal Women: A Randomized Controlled Pilot Study	Journal of Bone and Mineral Research. 19:352-359	4
Russo et al. (2003)	High-frequency vibration training increases muscle power in postmenopausal women	Archives of Physical Medicine and Rehabilitation. 84:1854-1857	4
Jo et al. (2021)	Effectiveness of Whole-Body Vibration Training to Improve Muscle Strength and Physical Performance in Older Adults: Prospective, Single-Blinded, Randomized Controlled Trial	Healthcare.31:652	5
Pouyafar et al. (2021)	Comparing the Effects of Eight Weeks of Whole Body Vibration Exercise Combined With Rope Skipping at Two Different Intensities on Physical Performance of Older Men: A Randomized Single-Blind Clinical Trial	Salmand: Iranian Journal of Ageing. 16:376-395	5
Bruin et al. (2020)	Combining Stochastic Resonance Vibration With Exergaming for Motor-Cognitive Training in Long-Term Care; A Sham-Control Randomized Controlled Pilot Trial	Frontiers in Medicine. 30:507155	5
Rogan et al. (2016)	Sensory-motor training targeting motor dysfunction and muscle weakness in long-term care elderly combined with motivational strategies: a single blind randomized controlled study	European Review of Aging and Physical Activity. 13:4	5
Ramos et al. (2019)	A single whole body vibration session influences quadriceps muscle strength, functional mobility and balance of elderly with osteopenia and/or osteoporosis? Pragmatic clinical trial	Journal of Diabetes & Metabolic Disorders. 18:73-80	6
Rogan et al. (2014)	Immediate effects after stochastic resonance whole-body vibration on physical performance on frail elderly for skilling-up training: a blind cross-over randomised pilot study	Aging Clinical and Experimental Research. 26:519–527	6

Giombini et al. (2013)	Acute Effect of Whole-Body Vibration at Optimal Frequency on Muscle Power Output of the Lower Limbs in Older Women	American Journal of Physical Medicine & Rehabilitation. 92:797-804	6
Cochrane et al. (2008)	A Comparison of the Physiologic Effects of Acute Whole-Body Vibration Exercise in Young and Older People	Archives of Physical Medicine and Rehabilitation. 89:815-821	6
Klarner et al. (2011)	Effekte unterschiedlicher Ganzkörpervibrationssysteme auf die neuromuskuläre Leistungsfähigkeit und die Körperzusammensetzung postmenopausaler Frauen Ergebnisse der kontrollierten randomisierten ELVIS-II-Studie	Deutsche Medizinische Wochenschrift. 136:2133-2139	8
Kemmler et al. (2010)	Effekte von Ganzkörpervibrationen auf die neuromuskuläre Leistungsfähigkeit von Frauen über dem 65. Lebensjahr: Einjahresergebnisse der kontrollierten randomisierten ELVIS-Studie	Zeitschrift für Gerontologie und Geriatrie. 43:125-132	8
Rees et al. (2008)	Effects of Whole-Body Vibration Exercise on Lower-Extremity Muscle Strength and Power in an Older Population: A Randomized Clinical Trial	Physical Therapy. 88:462-470	8

* Reasons: 1 – Not RCT (Randomized Clinical Trial); 2 – Not sinusoidal WBV; 3 – Did not assess outcomes of interest; 4 – Age group < 60 years; 5 – WBV associated with another intervention; 6 – Only evaluated acute effect; 7 – Did not stand during WBV; 8 – Information in another RCT included.

a)

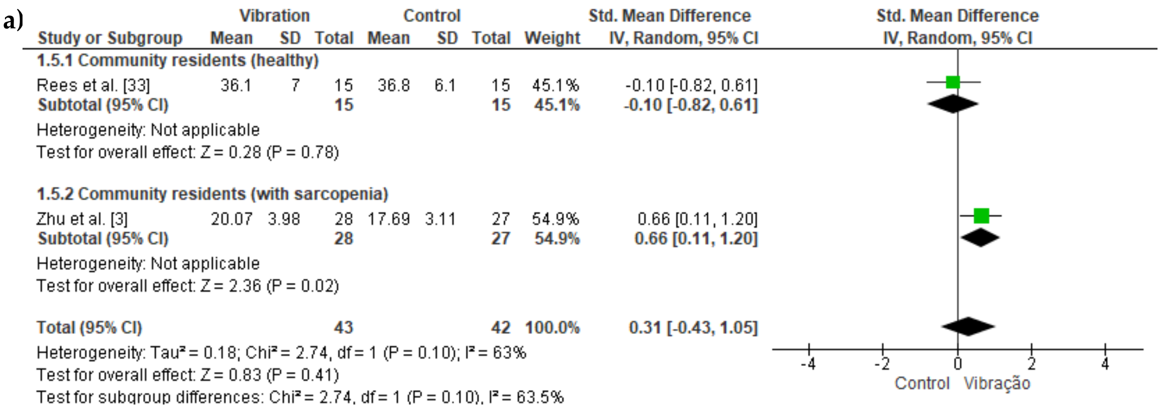

b

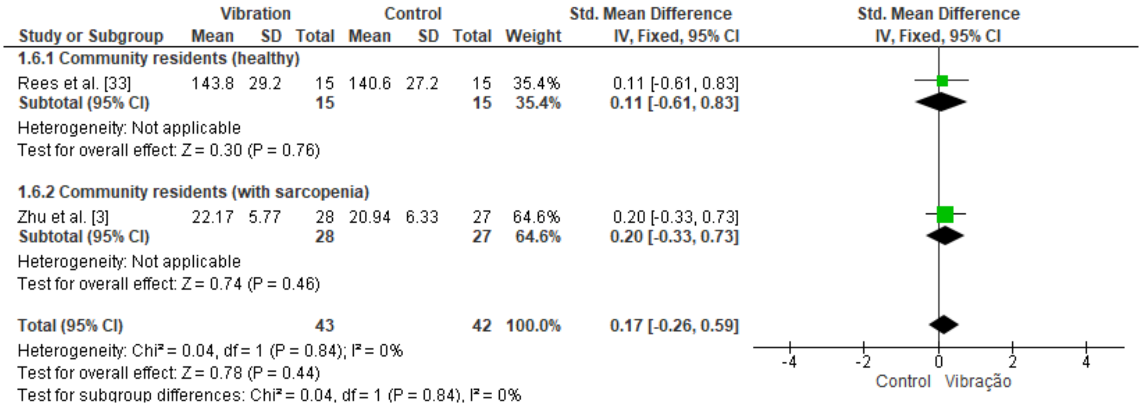

c)

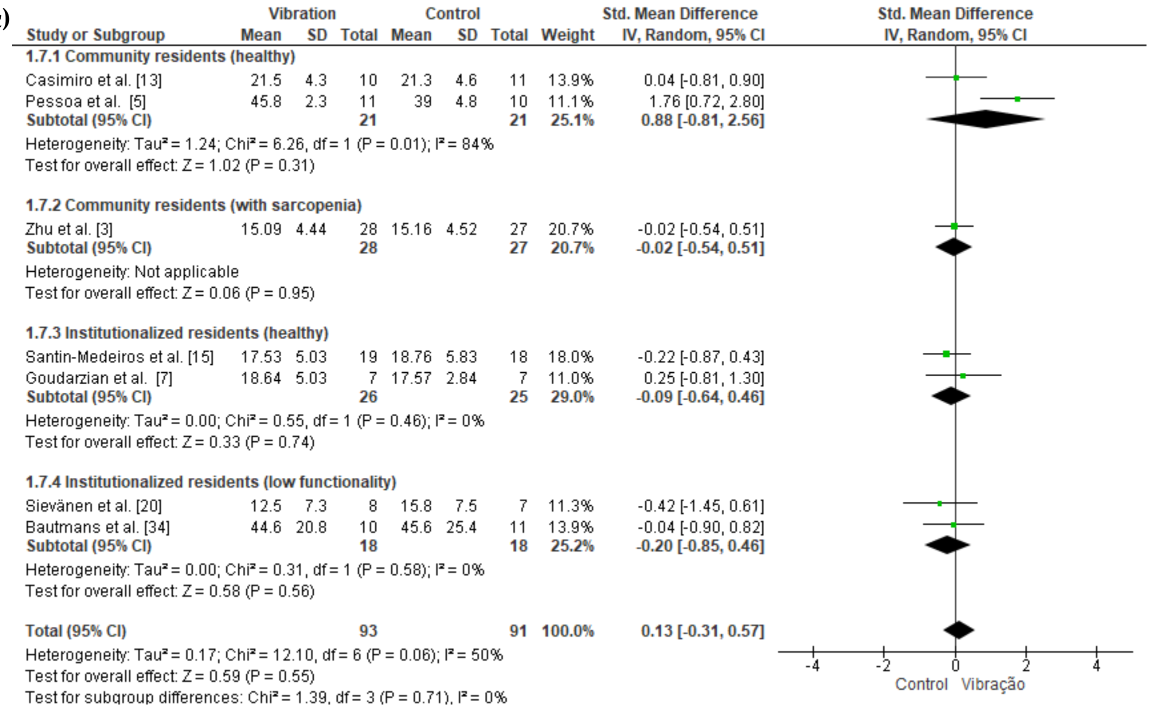

Supplementary Figure S1. Primary analysis comparing the effectiveness of WBV vs. control groups for muscle strength: a) ankle dorsiflexors; b) hip flexors; c) hand grip.

a)

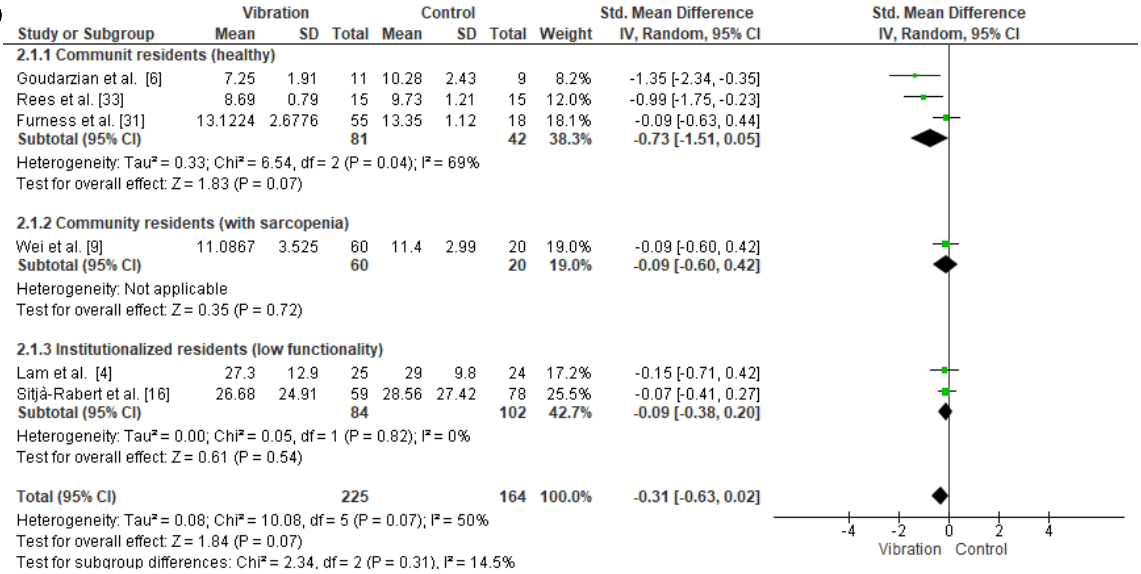

b

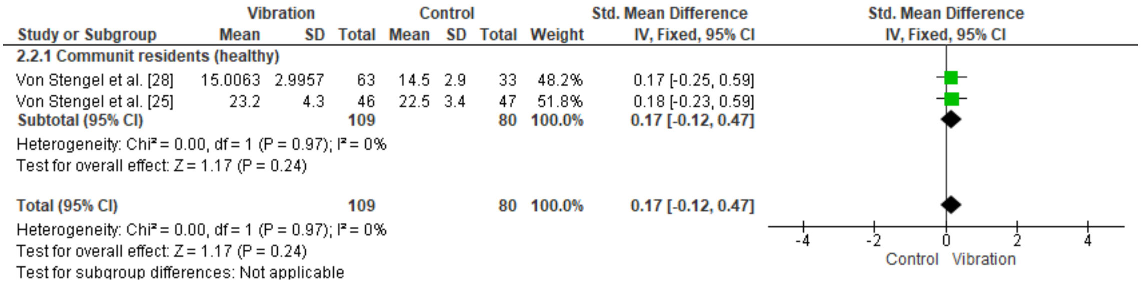

Supplementary Figure S2. Primary analysis comparing the effectiveness of WBV vs. control groups for muscle power: a) five-times-sit-to-stand test; b) countermovement jump.

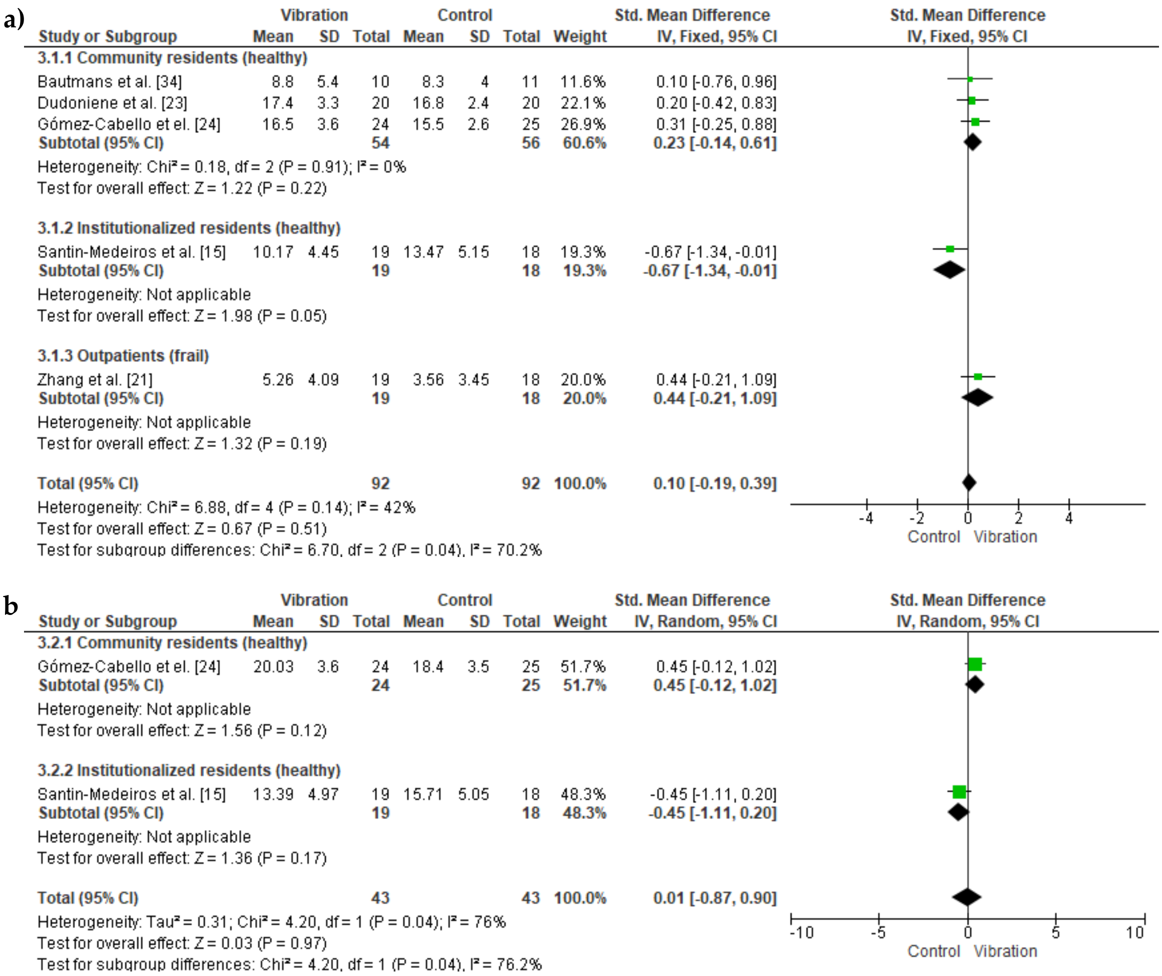

Supplementary Figure S3. Primary analysis comparing the effectiveness of WBV vs. control groups for muscular endurance: a) 30-second sit to stand; b) 30-second arm curl.

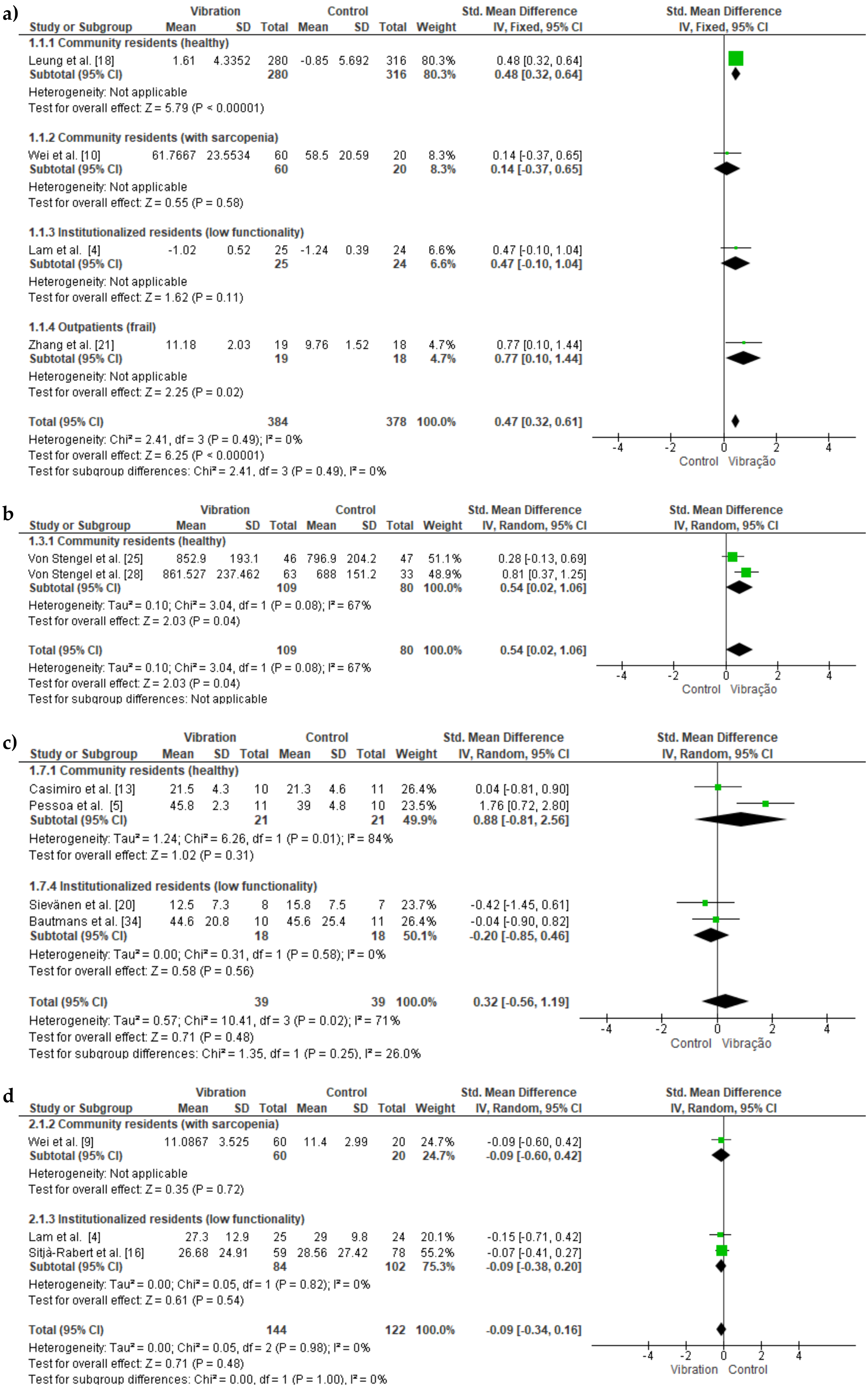
a)
Vibration Control Std. Mean Difference Std. Mean Difference
Study or Subgroup Mean SD Total Mean SD Total Weight IV, Fixed, 95% CI IV, Fixed, 95% CI
1.1.1 Community residents (healthy)
Leung et al. [18] 1.61 4.3352 280 -0.85 5.692 316 80.3% 0.48 [0.32, 0.64]
Subtotal (95% CI) 280 316 80.3% 0.48 [0.32, 0.64]
Heterogeneity: Not applicable
Test for overall effect: Z = 5.79 (P < 0.00001)
1.1.2 Community residents (with sarcopenia)
Wei et al. [10] 61.7667 23.5534 60 58.5 20.59 20 8.3% 0.14 [-0.37, 0.65]
Subtotal (95% CI) 60 20 8.3% 0.14 [-0.37, 0.65]
Heterogeneity: Not applicable
Test for overall effect: Z = 0.55 (P = 0.58)
1.1.3 Institutionalized residents (low functionality)
Lam et al. [4] -1.02 0.52 25 -1.24 0.39 24 6.6% 0.47 [-0.10, 1.04]
Subtotal (95% CI) 25 24 6.6% 0.47 [-0.10, 1.04]
Heterogeneity: Not applicable
Test for overall effect: Z = 1.62 (P = 0.11)
1.1.4 Outpatients (frail)
Zhang et al. [21] 11.18 2.03 19 9.76 1.52 18 4.7% 0.77 [0.10, 1.44]
Subtotal (95% CI) 19 18 4.7% 0.77 [0.10, 1.44]
Heterogeneity: Not applicable
Test for overall effect: Z = 2.25 (P = 0.02)
Total (95% CI) 384 378 100.0% 0.47 [0.32, 0.61]
Heterogeneity: Chi² = 2.41, df = 3 (P = 0.49); I² = 0%
Test for overall effect: Z = 6.25 (P < 0.00001)
Test for subgroup differences: Chi² = 2.41, df = 3 (P = 0.49), I² = 0%
-4 -2 0 2 4
Control Vibração
b
Vibration Control Std. Mean Difference Std. Mean Difference
Study or Subgroup Mean SD Total Mean SD Total Weight IV, Random, 95% CI IV, Random, 95% CI
1.3.1 Community residents (healthy)
Von Stengel et al. [25] 852.9 193.1 46 796.9 204.2 47 51.1% 0.28 [-0.13, 0.69]
Von Stengel et al. [28] 861.527 237.462 63 688 151.2 33 48.9% 0.81 [0.37, 1.25]
Subtotal (95% CI) 109 80 100.0% 0.54 [0.02, 1.06]
Heterogeneity: Tau² = 0.10; Chi² = 3.04, df = 1 (P = 0.08); I² = 67%
Test for overall effect: Z = 2.03 (P = 0.04)
Total (95% CI) 109 80 100.0% 0.54 [0.02, 1.06]
Heterogeneity: Tau² = 0.10; Chi² = 3.04, df = 1 (P = 0.08); I² = 67%
Test for overall effect: Z = 2.03 (P = 0.04)
Test for subgroup differences: Not applicable
-4 -2 0 2 4
Control Vibração
c)
Vibration Control Std. Mean Difference Std. Mean Difference
Study or Subgroup Mean SD Total Mean SD Total Weight IV, Random, 95% CI IV, Random, 95% CI
1.7.1 Community residents (healthy)
Casimiro et al. [13] 21.5 4.3 10 21.3 4.6 11 26.4% 0.04 [-0.81, 0.90]
Pessoa et al. [5] 45.8 2.3 11 39 4.8 10 23.5% 1.76 [0.72, 2.80]
Subtotal (95% CI) 21 21 49.9% 0.88 [-0.81, 2.56]
Heterogeneity: Tau² = 1.24; Chi² = 6.26, df = 1 (P = 0.01); I² = 84%
Test for overall effect: Z = 1.02 (P = 0.31)
1.7.4 Institutionalized residents (low functionality)
Sievänen et al. [20] 12.5 7.3 8 15.8 7.5 7 23.7% -0.42 [-1.45, 0.61]
Bautmans et al. [34] 44.6 20.8 10 45.6 25.4 11 26.4% -0.04 [-0.90, 0.82]
Subtotal (95% CI) 18 18 50.1% -0.20 [-0.85, 0.46]
Heterogeneity: Tau² = 0.00; Chi² = 0.31, df = 1 (P = 0.58); I² = 0%
Test for overall effect: Z = 0.58 (P = 0.56)
Total (95% CI) 39 39 100.0% 0.32 [-0.56, 1.19]
Heterogeneity: Tau² = 0.57; Chi² = 10.41, df = 3 (P = 0.02); I² = 71%
Test for overall effect: Z = 0.71 (P = 0.48)
Test for subgroup differences: Chi² = 1.35, df = 1 (P = 0.25), I² = 26.0%
-4 -2 0 2 4
Control Vibração
d
Vibration Control Std. Mean Difference Std. Mean Difference
Study or Subgroup Mean SD Total Mean SD Total Weight IV, Random, 95% CI IV, Random, 95% CI
2.1.2 Community residents (with sarcopenia)
Wei et al. [9] 11.0867 3.525 60 11.4 2.99 20 24.7% -0.09 [-0.60, 0.42]
Subtotal (95% CI) 60 20 24.7% -0.09 [-0.60, 0.42]
Heterogeneity: Not applicable
Test for overall effect: Z = 0.35 (P = 0.72)
2.1.3 Institutionalized residents (low functionality)
Lam et al. [4] 27.3 12.9 25 29 9.8 24 20.1% -0.15 [-0.71, 0.42]
Sitjà-Rabert et al. [16] 26.68 24.91 59 28.56 27.42 78 55.2% -0.07 [-0.41, 0.27]
Subtotal (95% CI) 84 102 75.3% -0.09 [-0.38, 0.20]
Heterogeneity: Tau² = 0.00; Chi² = 0.05, df = 1 (P = 0.82); I² = 0%
Test for overall effect: Z = 0.61 (P = 0.54)
Total (95% CI) 144 122 100.0% -0.09 [-0.34, 0.16]
Heterogeneity: Tau² = 0.00; Chi² = 0.05, df = 2 (P = 0.98); I² = 0%
Test for overall effect: Z = 0.71 (P = 0.48)
Test for subgroup differences: Chi² = 0.00, df = 1 (P = 1.00), I² = 0%
-4 -2 0 2 4
Vibration Control

e)

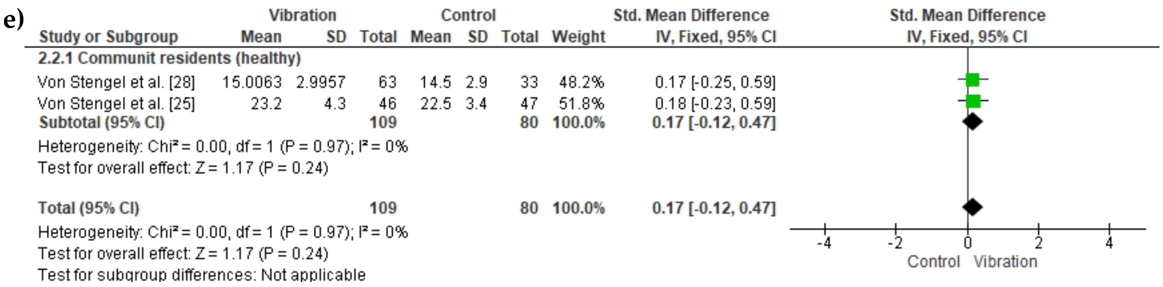

f)

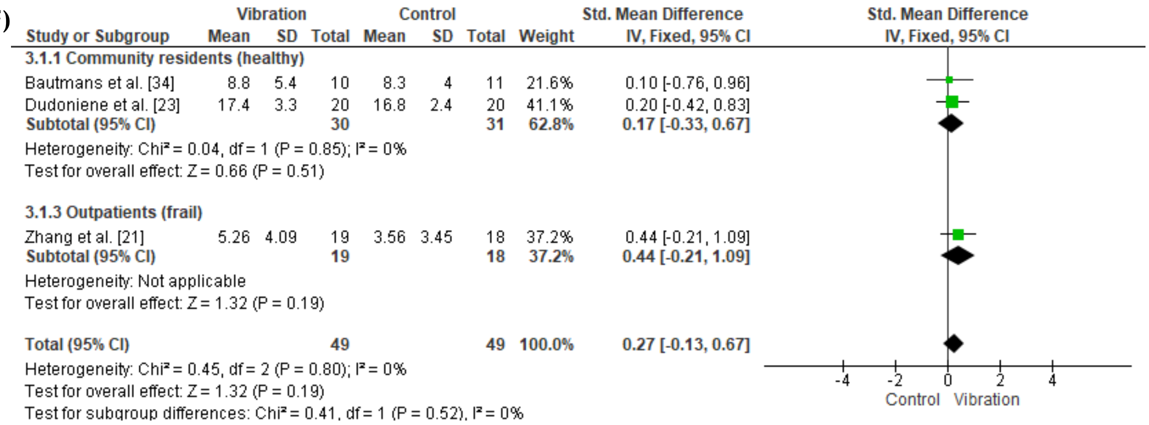

Supplementary Figure S4. Sensitivity analysis comparing the effectiveness of WBV vs. control groups for: a) knee extensor muscle strength; b) muscular strength of the extensors of the lower limbs; c) handgrip strength; d) five-times-sit-to-stand; e) countermovement jump; f) 30-second sit-to-stand test.

a)

Study or Subgroup	Vibration Mean	Vibration SD	Vibration Total	Exercise Mean	Exercise SD	Exercise Total	Weight	Std. Mean Difference IV, Fixed, 95% CI
**1.1.1 Community residents (healthy)**								
Rees et al. [33]	177.8	34.4	15	179.5	41.8	13	35.0%	-0.04 [-0.79, 0.70]
**Subtotal (95% CI)**			**15**			**13**	**35.0%**	**-0.04 [-0.79, 0.70]**
Heterogeneity: Not applicable								
Test for overall effect: Z = 0.11 (P = 0.91)								
**1.1.2 Community residents (with sarcopenia)**								
Zhu et al. [3]	19.74	3.92	28	19.63	3.04	24	65.0%	0.03 [-0.51, 0.58]
**Subtotal (95% CI)**			**28**			**24**	**65.0%**	**0.03 [-0.51, 0.58]**
Heterogeneity: Not applicable								
Test for overall effect: Z = 0.11 (P = 0.91)								
**Total (95% CI)**			**43**			**37**	**100.0%**	**0.00 [-0.43, 0.44]**

Heterogeneity: Chi² = 0.02, df = 1 (P = 0.87); I² = 0%
Test for overall effect: Z = 0.02 (P = 0.98)
Test for subgroup differences: Chi² = 0.02, df = 1 (P = 0.87), I² = 0%

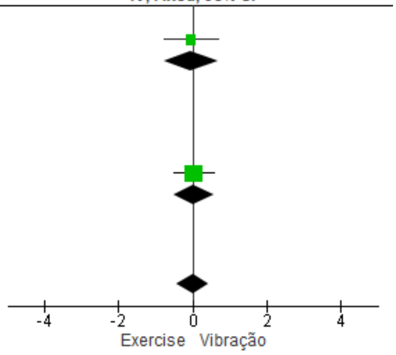

b

Study or Subgroup	Vibration Mean	Vibration SD	Vibration Total	Exercise Mean	Exercise SD	Exercise Total	Weight	Std. Mean Difference IV, Fixed, 95% CI
**1.2.1 Community residents (healthy)**								
Rees et al. [33]	113.6	29.4	15	116.1	36.3	13	35.3%	-0.07 [-0.82, 0.67]
**Subtotal (95% CI)**			**15**			**13**	**35.3%**	**-0.07 [-0.82, 0.67]**
Heterogeneity: Not applicable								
Test for overall effect: Z = 0.20 (P = 0.85)								
**1.2.2 Community residents (with sarcopenia)**								
Zhu et al. [3]	23.9	3.42	28	22.8	4	24	64.7%	0.29 [-0.26, 0.84]
**Subtotal (95% CI)**			**28**			**24**	**64.7%**	**0.29 [-0.26, 0.84]**
Heterogeneity: Not applicable								
Test for overall effect: Z = 1.05 (P = 0.30)								
**Total (95% CI)**			**43**			**37**	**100.0%**	**0.16 [-0.28, 0.60]**

Heterogeneity: Chi² = 0.61, df = 1 (P = 0.44); I² = 0%
Test for overall effect: Z = 0.73 (P = 0.47)
Test for subgroup differences: Chi² = 0.61, df = 1 (P = 0.44), I² = 0%

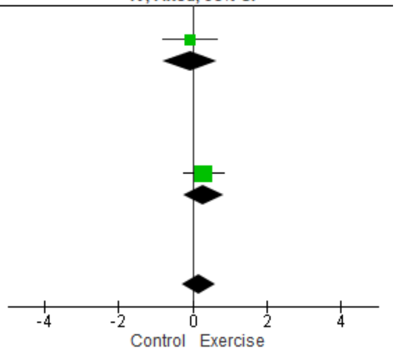

c)

Study or Subgroup	Vibration Mean	Vibration SD	Vibration Total	Exercise Mean	Exercise SD	Exercise Total	Weight	Std. Mean Difference IV, Random, 95% CI
**1.5.1 Community residents (healthy)**								
Rees et al. [33]	36.1	7	15	37.4	10.7	13	35.0%	-0.14 [-0.89, 0.60]
**Subtotal (95% CI)**			**15**			**13**	**35.0%**	**-0.14 [-0.89, 0.60]**
Heterogeneity: Not applicable								
Test for overall effect: Z = 0.37 (P = 0.71)								
**1.5.2 Community residents (with sarcopenia)**								
Zhu et al. [3]	20.07	3.98	28	20.25	4.65	24	65.0%	-0.04 [-0.59, 0.50]
**Subtotal (95% CI)**			**28**			**24**	**65.0%**	**-0.04 [-0.59, 0.50]**
Heterogeneity: Not applicable								
Test for overall effect: Z = 0.15 (P = 0.88)								
**Total (95% CI)**			**43**			**37**	**100.0%**	**-0.08 [-0.52, 0.36]**

Heterogeneity: Tau² = 0.00; Chi² = 0.05, df = 1 (P = 0.83); I² = 0%
Test for overall effect: Z = 0.34 (P = 0.73)
Test for subgroup differences: Chi² = 0.05, df = 1 (P = 0.83), I² = 0%

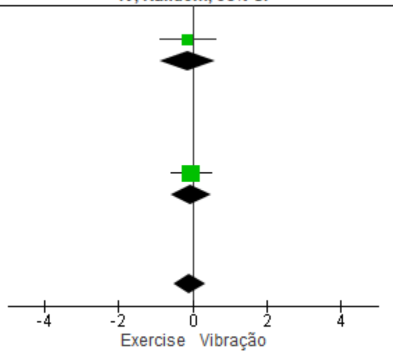

d

Study or Subgroup	Vibration Mean	Vibration SD	Vibration Total	Exercise Mean	Exercise SD	Exercise Total	Weight	Std. Mean Difference IV, Random, 95% CI
**1.6.1 Community residents (healthy)**								
Rees et al. [33]	143.8	29.2	15	141.2	44.3	13	46.4%	0.07 [-0.67, 0.81]
**Subtotal (95% CI)**			**15**			**13**	**46.4%**	**0.07 [-0.67, 0.81]**
Heterogeneity: Not applicable								
Test for overall effect: Z = 0.18 (P = 0.86)								
**1.6.2 Community residents (with sarcopenia)**								
Zhu et al. [3]	22.17	5.77	28	26.78	5.02	24	53.6%	-0.83 [-1.41, -0.26]
**Subtotal (95% CI)**			**28**			**24**	**53.6%**	**-0.83 [-1.41, -0.26]**
Heterogeneity: Not applicable								
Test for overall effect: Z = 2.87 (P = 0.004)								
**Total (95% CI)**			**43**			**37**	**100.0%**	**-0.42 [-1.30, 0.47]**

Heterogeneity: Tau² = 0.29; Chi² = 3.57, df = 1 (P = 0.06); I² = 72%
Test for overall effect: Z = 0.92 (P = 0.36)
Test for subgroup differences: Chi² = 3.57, df = 1 (P = 0.06), I² = 72.0%

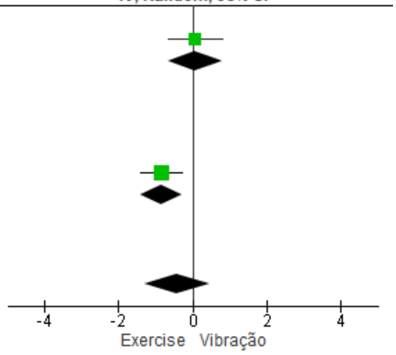

e)

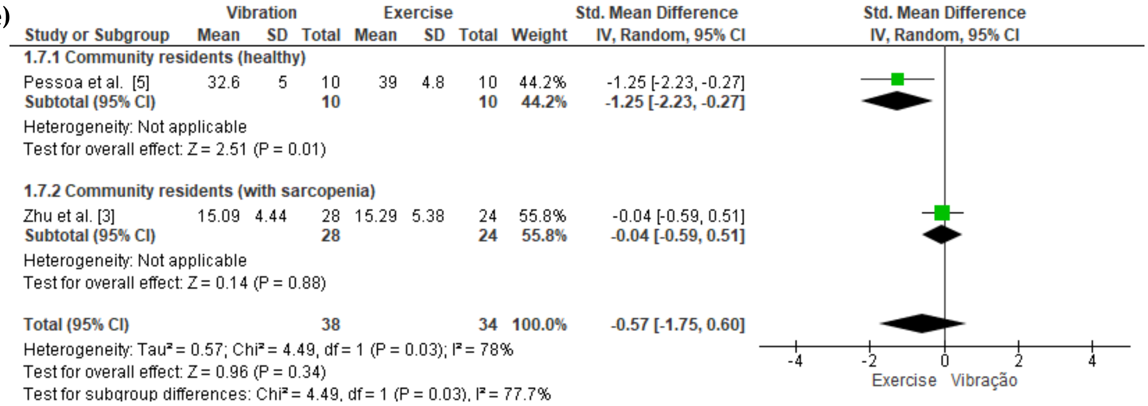

Supplementary Figure S5. Analysis comparing the effectiveness of WBV vs. other forms of exercise for muscle strength: a) knee extensors; b) knee flexors; c) ankle dorsiflexors; d) hip flexors; e) hand grip.

a)

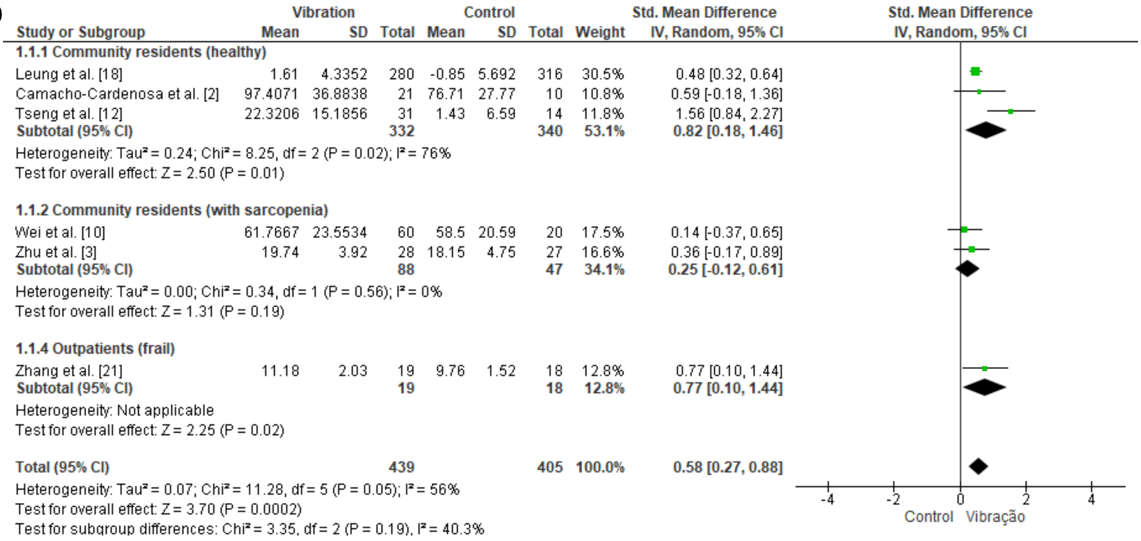

Vibration
Control
Std. Mean Difference
Std. Mean Difference
Study or Subgroup Mean SD Total Mean SD Total Weight IV, Random, 95% CI
IV, Random, 95% CI
1.1.1 Community residents (healthy)
Leung et al. [18] 1.61 4.3352 280 -0.85 5.692 316 30.5% 0.48 [0.32, 0.64]
Camacho-Cardenosa et al. [2] 97.4071 36.8838 21 76.71 27.77 10 10.8% 0.59 [-0.18, 1.36]
Tseng et al. [12] 22.3206 15.1856 31 1.43 6.59 14 11.8% 1.56 [0.84, 2.27]
Subtotal (95% CI) 332 340 53.1% 0.82 [0.18, 1.46]
Heterogeneity: Tau² = 0.24; Chi² = 8.25, df = 2 (P = 0.02); I² = 76%
Test for overall effect: Z = 2.50 (P = 0.01)
1.1.2 Community residents (with sarcopenia)
Wei et al. [10] 61.7667 23.5534 60 58.5 20.59 20 17.5% 0.14 [-0.37, 0.65]
Zhu et al. [3] 19.74 3.92 28 18.15 4.75 27 16.6% 0.36 [-0.17, 0.89]
Subtotal (95% CI) 88 47 34.1% 0.25 [-0.12, 0.61]
Heterogeneity: Tau² = 0.00; Chi² = 0.34, df = 1 (P = 0.56); I² = 0%
Test for overall effect: Z = 1.31 (P = 0.19)
1.1.4 Outpatients (frail)
Zhang et al. [21] 11.18 2.03 19 9.76 1.52 18 12.8% 0.77 [0.10, 1.44]
Subtotal (95% CI) 19 18 12.8% 0.77 [0.10, 1.44]
Heterogeneity: Not applicable
Test for overall effect: Z = 2.25 (P = 0.02)
Total (95% CI) 439 405 100.0% 0.58 [0.27, 0.88]
Heterogeneity: Tau² = 0.07; Chi² = 11.28, df = 5 (P = 0.05); I² = 56%
Test for overall effect: Z = 3.70 (P = 0.0002)
Test for subgroup differences: Chi² = 3.35, df = 2 (P = 0.19), I² = 40.3%
-4 -2 0 2 4
Control Vibração

b

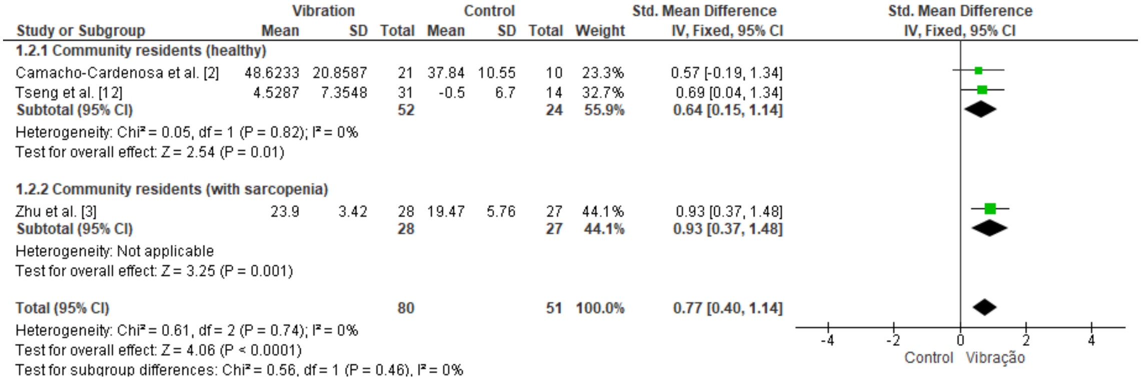

Vibration
Control
Std. Mean Difference
Std. Mean Difference
Study or Subgroup Mean SD Total Mean SD Total Weight IV, Fixed, 95% CI
IV, Fixed, 95% CI
1.2.1 Community residents (healthy)
Camacho-Cardenosa et al. [2] 48.6233 20.8587 21 37.84 10.55 10 23.3% 0.57 [-0.19, 1.34]
Tseng et al. [12] 4.5287 7.3548 31 -0.5 6.7 14 32.7% 0.69 [0.04, 1.34]
Subtotal (95% CI) 52 24 55.9% 0.64 [0.15, 1.14]
Heterogeneity: Chi² = 0.05, df = 1 (P = 0.82); I² = 0%
Test for overall effect: Z = 2.54 (P = 0.01)
1.2.2 Community residents (with sarcopenia)
Zhu et al. [3] 23.9 3.42 28 19.47 5.76 27 44.1% 0.93 [0.37, 1.48]
Subtotal (95% CI) 28 27 44.1% 0.93 [0.37, 1.48]
Heterogeneity: Not applicable
Test for overall effect: Z = 3.25 (P = 0.001)
Total (95% CI) 80 51 100.0% 0.77 [0.40, 1.14]
Heterogeneity: Chi² = 0.61, df = 2 (P = 0.74); I² = 0%
Test for overall effect: Z = 4.06 (P < 0.0001)
Test for subgroup differences: Chi² = 0.56, df = 1 (P = 0.46), I² = 0%
-4 -2 0 2 4
Control Vibração

c)

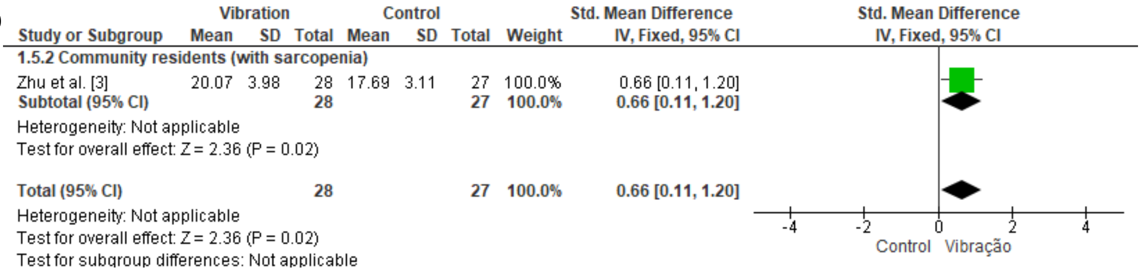

Vibration
Control
Std. Mean Difference
Std. Mean Difference
Study or Subgroup Mean SD Total Mean SD Total Weight IV, Fixed, 95% CI
IV, Fixed, 95% CI
1.5.2 Community residents (with sarcopenia)
Zhu et al. [3] 20.07 3.98 28 17.69 3.11 27 100.0% 0.66 [0.11, 1.20]
Subtotal (95% CI) 28 27 100.0% 0.66 [0.11, 1.20]
Heterogeneity: Not applicable
Test for overall effect: Z = 2.36 (P = 0.02)
Total (95% CI) 28 27 100.0% 0.66 [0.11, 1.20]
Heterogeneity: Not applicable
Test for overall effect: Z = 2.36 (P = 0.02)
Test for subgroup differences: Not applicable
-4 -2 0 2 4
Control Vibração

d

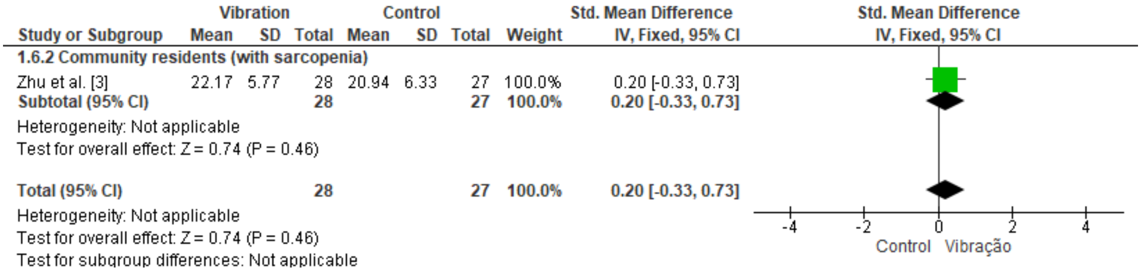

Vibration
Control
Std. Mean Difference
Std. Mean Difference
Study or Subgroup Mean SD Total Mean SD Total Weight IV, Fixed, 95% CI
IV, Fixed, 95% CI
1.6.2 Community residents (with sarcopenia)
Zhu et al. [3] 22.17 5.77 28 20.94 6.33 27 100.0% 0.20 [-0.33, 0.73]
Subtotal (95% CI) 28 27 100.0% 0.20 [-0.33, 0.73]
Heterogeneity: Not applicable
Test for overall effect: Z = 0.74 (P = 0.46)
Total (95% CI) 28 27 100.0% 0.20 [-0.33, 0.73]
Heterogeneity: Not applicable
Test for overall effect: Z = 0.74 (P = 0.46)
Test for subgroup differences: Not applicable
-4 -2 0 2 4
Control Vibração

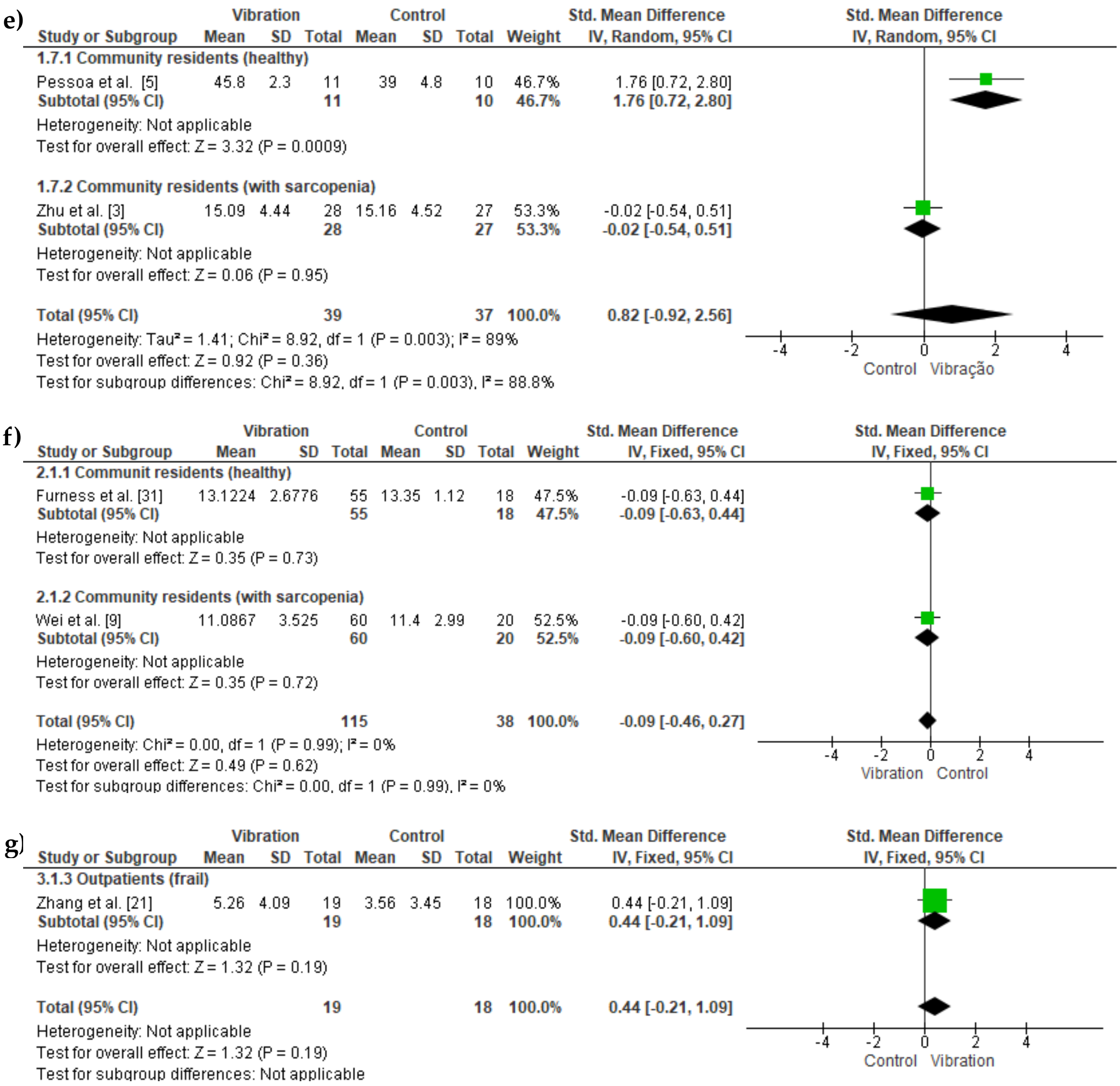

Supplementary Figure S6. Analysis comparing the effectiveness of WBV with participants maintaining a static position vs. control groups: a) muscle strength of the knee extensors; b) muscle strength of the knee flexors; c) muscular strength of the ankle dorsiflexors; d) muscle strength of the hip flexors; e) handgrip muscle strength; f) five-times-sit-to-stand; g) 30-s sit-to-stand.

a)

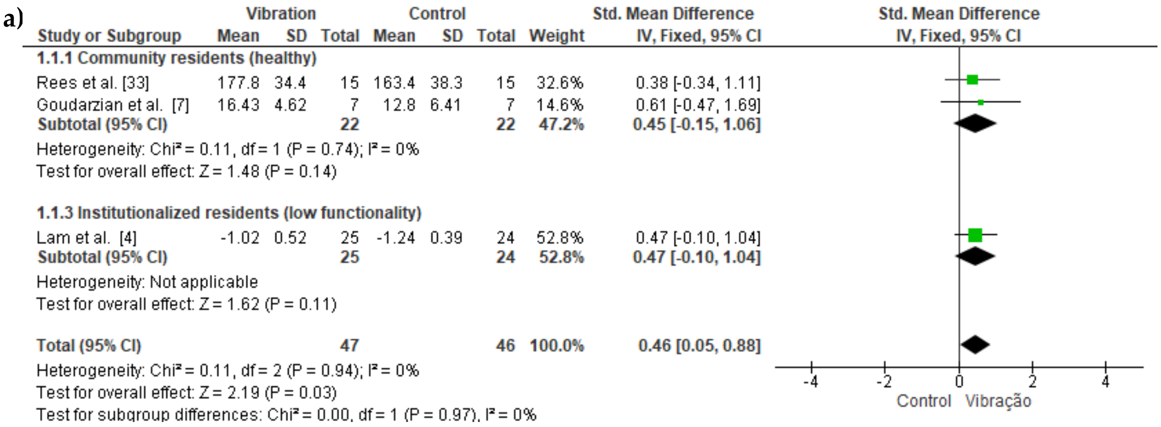
Vibration Control Std. Mean Difference Std. Mean Difference
Study or Subgroup Mean SD Total Mean SD Total Weight IV, Fixed, 95% CI IV, Fixed, 95% CI
1.1.1 Community residents (healthy)
Rees et al. [33] 177.8 34.4 15 163.4 38.3 15 32.6% 0.38 [-0.34, 1.11]
Goudarzian et al. [7] 16.43 4.62 7 12.8 6.41 7 14.6% 0.61 [-0.47, 1.69]
Subtotal (95% CI) 22 22 47.2% 0.45 [-0.15, 1.06]
Heterogeneity: Chi² = 0.11, df = 1 (P = 0.74); I² = 0%
Test for overall effect: Z = 1.48 (P = 0.14)
1.1.3 Institutionalized residents (low functionality)
Lam et al. [4] -1.02 0.52 25 -1.24 0.39 24 52.8% 0.47 [-0.10, 1.04]
Subtotal (95% CI) 25 24 52.8% 0.47 [-0.10, 1.04]
Heterogeneity: Not applicable
Test for overall effect: Z = 1.62 (P = 0.11)
Total (95% CI) 47 46 100.0% 0.46 [0.05, 0.88]
Heterogeneity: Chi² = 0.11, df = 2 (P = 0.94); I² = 0%
Test for overall effect: Z = 2.19 (P = 0.03)
Test for subgroup differences: Chi² = 0.00, df = 1 (P = 0.97), I² = 0%
-4 -2 0 2 4
Control Vibração

b

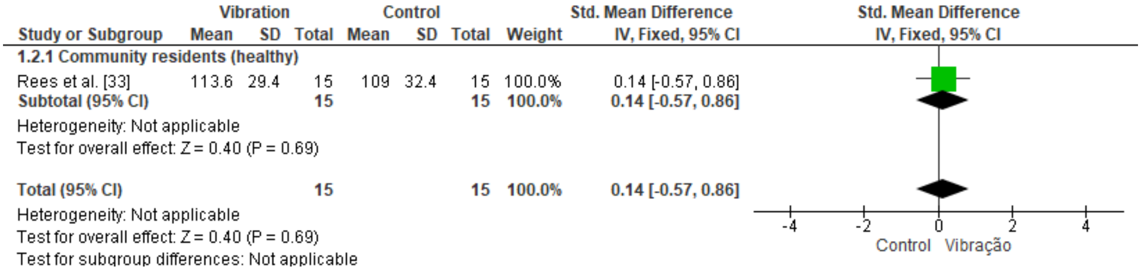
Vibration Control Std. Mean Difference Std. Mean Difference
Study or Subgroup Mean SD Total Mean SD Total Weight IV, Fixed, 95% CI IV, Fixed, 95% CI
1.2.1 Community residents (healthy)
Rees et al. [33] 113.6 29.4 15 109 32.4 15 100.0% 0.14 [-0.57, 0.86]
Subtotal (95% CI) 15 15 100.0% 0.14 [-0.57, 0.86]
Heterogeneity: Not applicable
Test for overall effect: Z = 0.40 (P = 0.69)
Total (95% CI) 15 15 100.0% 0.14 [-0.57, 0.86]
Heterogeneity: Not applicable
Test for overall effect: Z = 0.40 (P = 0.69)
Test for subgroup differences: Not applicable
-4 -2 0 2 4
Control Vibração

c)

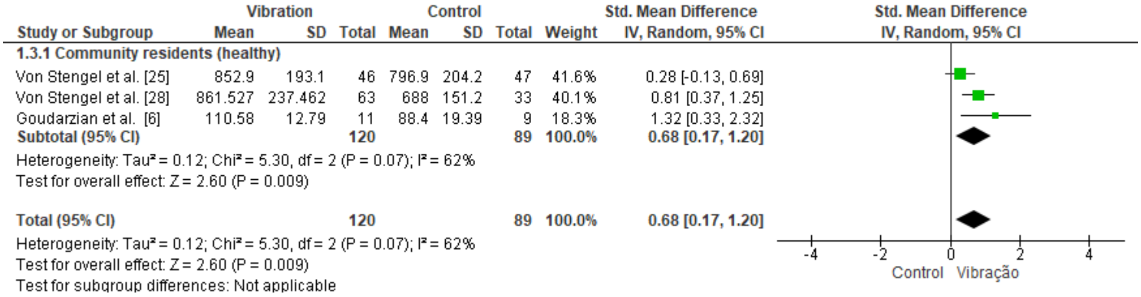
Vibration Control Std. Mean Difference Std. Mean Difference
Study or Subgroup Mean SD Total Mean SD Total Weight IV, Random, 95% CI IV, Random, 95% CI
1.3.1 Community residents (healthy)
Von Stengel et al. [25] 852.9 193.1 46 796.9 204.2 47 41.6% 0.28 [-0.13, 0.69]
Von Stengel et al. [28] 861.527 237.462 63 688 151.2 33 40.1% 0.81 [0.37, 1.25]
Goudarzian et al. [6] 110.58 12.79 11 88.4 19.39 9 18.3% 1.32 [0.33, 2.32]
Subtotal (95% CI) 120 89 100.0% 0.68 [0.17, 1.20]
Heterogeneity: Tau² = 0.12; Chi² = 5.30, df = 2 (P = 0.07); I² = 62%
Test for overall effect: Z = 2.60 (P = 0.009)
Total (95% CI) 120 89 100.0% 0.68 [0.17, 1.20]
Heterogeneity: Tau² = 0.12; Chi² = 5.30, df = 2 (P = 0.07); I² = 62%
Test for overall effect: Z = 2.60 (P = 0.009)
Test for subgroup differences: Not applicable
-4 -2 0 2 4
Control Vibração

d

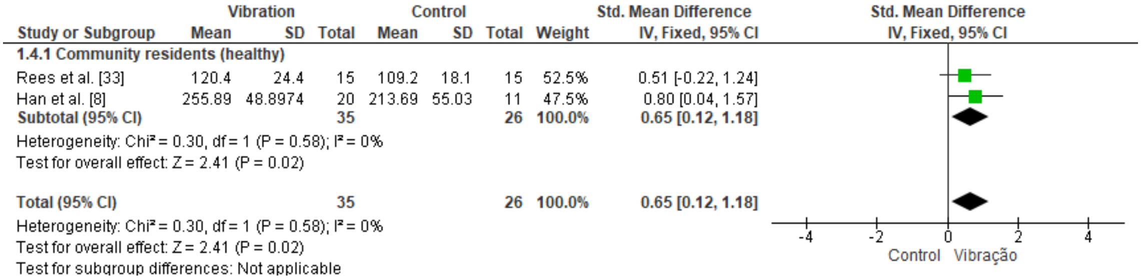
Vibration Control Std. Mean Difference Std. Mean Difference
Study or Subgroup Mean SD Total Mean SD Total Weight IV, Fixed, 95% CI IV, Fixed, 95% CI
1.4.1 Community residents (healthy)
Rees et al. [33] 120.4 24.4 15 109.2 18.1 15 52.5% 0.51 [-0.22, 1.24]
Han et al. [8] 255.89 48.8974 20 213.69 55.03 11 47.5% 0.80 [0.04, 1.57]
Subtotal (95% CI) 35 26 100.0% 0.65 [0.12, 1.18]
Heterogeneity: Chi² = 0.30, df = 1 (P = 0.58); I² = 0%
Test for overall effect: Z = 2.41 (P = 0.02)
Total (95% CI) 35 26 100.0% 0.65 [0.12, 1.18]
Heterogeneity: Chi² = 0.30, df = 1 (P = 0.58); I² = 0%
Test for overall effect: Z = 2.41 (P = 0.02)
Test for subgroup differences: Not applicable
-4 -2 0 2 4
Control Vibração

e)

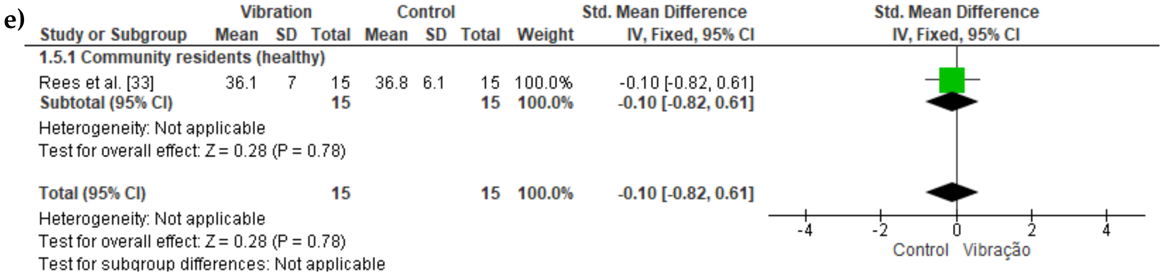
Vibration Control Std. Mean Difference Std. Mean Difference
Study or Subgroup Mean SD Total Mean SD Total Weight IV, Fixed, 95% CI IV, Fixed, 95% CI
1.5.1 Community residents (healthy)
Rees et al. [33] 36.1 7 15 36.8 6.1 15 100.0% -0.10 [-0.82, 0.61]
Subtotal (95% CI) 15 15 100.0% -0.10 [-0.82, 0.61]
Heterogeneity: Not applicable
Test for overall effect: Z = 0.28 (P = 0.78)
Total (95% CI) 15 15 100.0% -0.10 [-0.82, 0.61]
Heterogeneity: Not applicable
Test for overall effect: Z = 0.28 (P = 0.78)
Test for subgroup differences: Not applicable
-4 -2 0 2 4
Control Vibração

f)

Study or Subgroup	Vibration Mean	Vibration SD	Vibration Total	Control Mean	Control SD	Control Total	Weight	Std. Mean Difference IV, Fixed, 95% CI
**1.6.1 Community residents (healthy)**								
Rees et al. [33]	143.8	29.2	15	140.6	27.2	15	100.0%	0.11 [-0.61, 0.83]
**Subtotal (95% CI)**			**15**			**15**	**100.0%**	**0.11 [-0.61, 0.83]**
Heterogeneity: Not applicable								
Test for overall effect: Z = 0.30 (P = 0.76)								
**Total (95% CI)**			**15**			**15**	**100.0%**	**0.11 [-0.61, 0.83]**
Heterogeneity: Not applicable								
Test for overall effect: Z = 0.30 (P = 0.76)								
Test for subgroup differences: Not applicable								

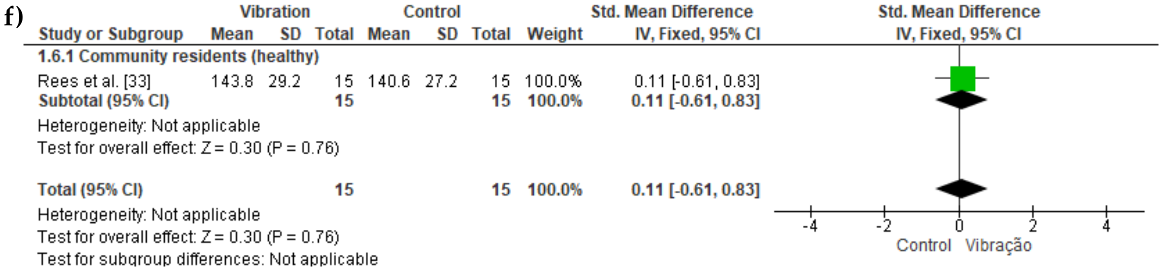

g)

Study or Subgroup	Vibration Mean	Vibration SD	Vibration Total	Control Mean	Control SD	Control Total	Weight	Std. Mean Difference IV, Fixed, 95% CI
**1.7.1 Community residents (healthy)**								
Casimiro et al. [13]	21.5	4.3	10	21.3	4.6	11	19.6%	0.04 [-0.81, 0.90]
**Subtotal (95% CI)**			**10**			**11**	**19.6%**	**0.04 [-0.81, 0.90]**
Heterogeneity: Not applicable								
Test for overall effect: Z = 0.10 (P = 0.92)								
**1.7.3 Institutionalized residents (healthy)**								
Santin-Medeiros et al. [15]	17.53	5.03	19	18.76	5.83	18	34.3%	-0.22 [-0.87, 0.43]
Goudarzian et al. [7]	18.64	5.03	7	17.57	2.84	7	13.0%	0.25 [-0.81, 1.30]
**Subtotal (95% CI)**			**26**			**25**	**47.3%**	**-0.09 [-0.64, 0.46]**
Heterogeneity: Chi² = 0.55, df = 1 (P = 0.46); I² = 0%								
Test for overall effect: Z = 0.33 (P = 0.74)								
**1.7.4 Institutionalized residents (low functionality)**								
Sievänen et al. [20]	12.5	7.3	8	15.8	7.5	7	13.6%	-0.42 [-1.45, 0.61]
Bautmans et al. [34]	44.6	20.8	10	45.6	25.4	11	19.6%	-0.04 [-0.90, 0.82]
**Subtotal (95% CI)**			**18**			**18**	**33.1%**	**-0.20 [-0.85, 0.46]**
Heterogeneity: Chi² = 0.31, df = 1 (P = 0.58); I² = 0%								
Test for overall effect: Z = 0.58 (P = 0.56)								
**Total (95% CI)**			**54**			**54**	**100.0%**	**-0.10 [-0.48, 0.28]**
Heterogeneity: Chi² = 1.05, df = 4 (P = 0.90); I² = 0%								
Test for overall effect: Z = 0.52 (P = 0.60)								
Test for subgroup differences: Chi² = 0.19, df = 2 (P = 0.91), I² = 0%								

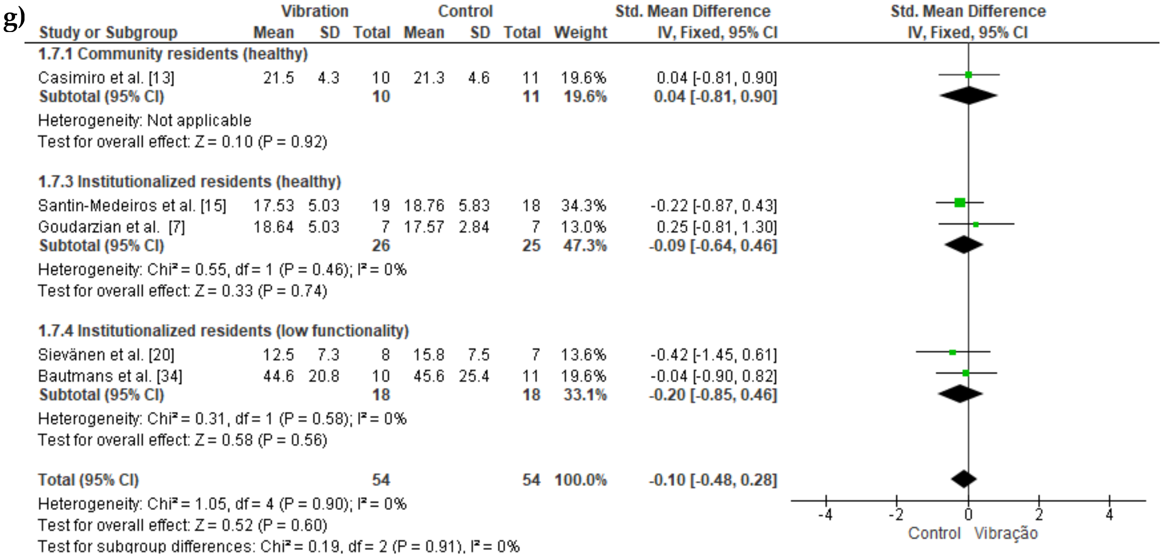

h

Study or Subgroup	Vibration Mean	Vibration SD	Vibration Total	Control Mean	Control SD	Control Total	Weight	Std. Mean Difference IV, Random, 95% CI
**2.1.1 Communit residents (healthy)**								
Goudarzian et al. [6]	7.25	1.91	11	10.28	2.43	9	16.8%	-1.35 [-2.34, -0.35]
Rees et al. [33]	8.69	0.79	15	9.73	1.21	15	21.9%	-0.99 [-1.75, -0.23]
**Subtotal (95% CI)**			**26**			**24**	**38.8%**	**-1.12 [-1.73, -0.52]**
Heterogeneity: Tau² = 0.00; Chi² = 0.31, df = 1 (P = 0.58); I² = 0%								
Test for overall effect: Z = 3.63 (P = 0.0003)								
**2.1.3 Institutionalized residents (low functionality)**								
Lam et al. [4]	27.3	12.9	25	29	9.8	24	27.5%	-0.15 [-0.71, 0.42]
Sitjà-Rabert et al. [16]	26.68	24.91	59	28.56	27.42	78	33.8%	-0.07 [-0.41, 0.27]
**Subtotal (95% CI)**			**84**			**102**	**61.2%**	**-0.09 [-0.38, 0.20]**
Heterogeneity: Tau² = 0.00; Chi² = 0.05, df = 1 (P = 0.82); I² = 0%								
Test for overall effect: Z = 0.61 (P = 0.54)								
**Total (95% CI)**			**110**			**126**	**100.0%**	**-0.51 [-1.05, 0.03]**
Heterogeneity: Tau² = 0.20; Chi² = 9.40, df = 3 (P = 0.02); I² = 68%								
Test for overall effect: Z = 1.83 (P = 0.07)								
Test for subgroup differences: Chi² = 9.04, df = 1 (P = 0.003), I² = 88.9%								

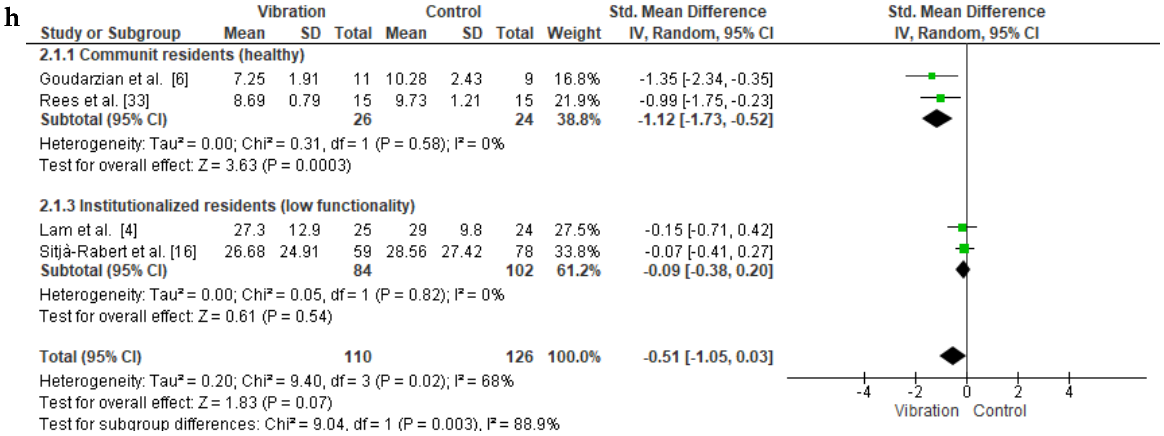

i)

Study or Subgroup	Vibration Mean	Vibration SD	Vibration Total	Control Mean	Control SD	Control Total	Weight	Std. Mean Difference IV, Fixed, 95% CI
**2.2.1 Communit residents (healthy)**								
Von Stengel et al. [28]	15.0063	2.9957	63	14.5	2.9	33	48.2%	0.17 [-0.25, 0.59]
Von Stengel et al. [25]	23.2	4.3	46	22.5	3.4	47	51.8%	0.18 [-0.23, 0.59]
**Subtotal (95% CI)**			**109**			**80**	**100.0%**	**0.17 [-0.12, 0.47]**
Heterogeneity: Chi² = 0.00, df = 1 (P = 0.97); I² = 0%								
Test for overall effect: Z = 1.17 (P = 0.24)								
**Total (95% CI)**			**109**			**80**	**100.0%**	**0.17 [-0.12, 0.47]**
Heterogeneity: Chi² = 0.00, df = 1 (P = 0.97); I² = 0%								
Test for overall effect: Z = 1.17 (P = 0.24)								
Test for subgroup differences: Not applicable								

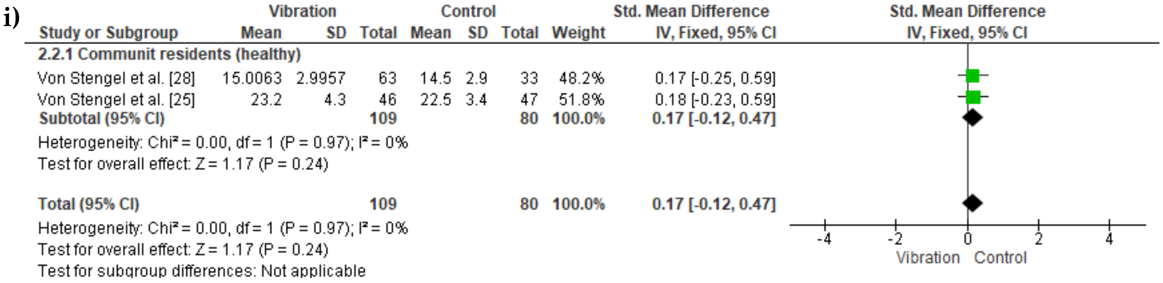

j)

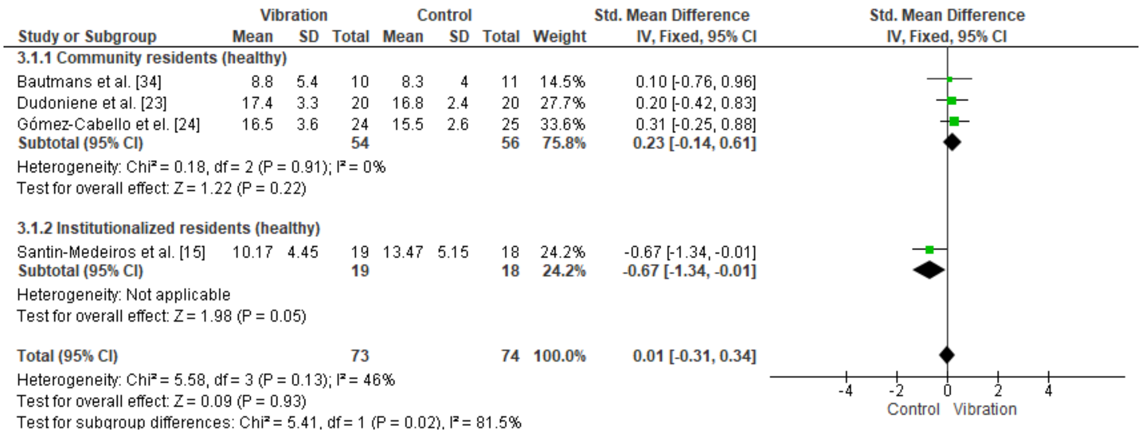

k

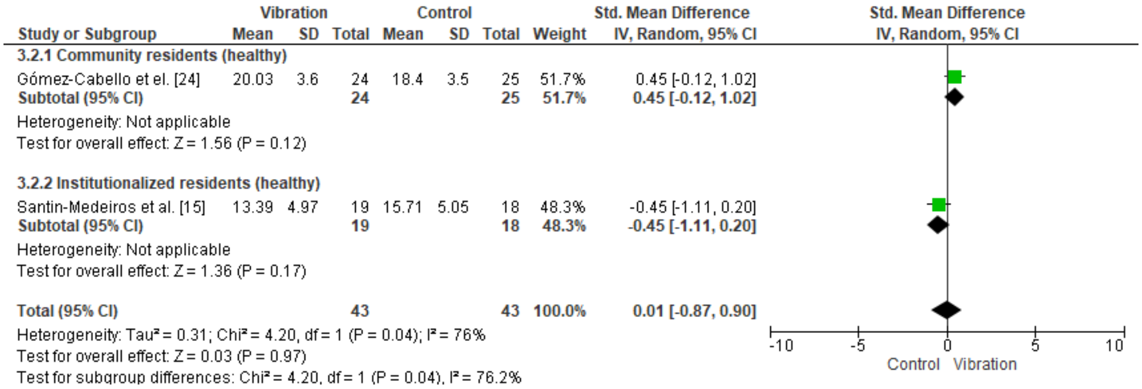

Supplementary Figure S7. Analysis comparing the effectiveness of WBV with participants performing muscle-strengthening exercises vs. control groups: a) muscle strength of the knee extensors; b) muscle strength of the knee flexors; c) muscular strength of the extensors of the lower limbs; d) muscular strength of the plantar flexors; e) muscular strength of the ankle dorsiflexors; f) muscle strength of the hip flexors; g) handgrip muscle strength; h) five-times-sit-to-stand; i) countermovement jump; j) 30-s sit-to-stand; k) 30-s arm curl.

a)

Study or Subgroup	Vibration Mean	Vibration SD	Vibration Total	Control Mean	Control SD	Control Total	Weight	Std. Mean Difference IV, Fixed, 95% CI
**1.1.1 Community residents (healthy)**								
Leung et al. [18]	1.61	4.3352	280	-0.85	5.692	316	84.3%	0.48 [0.32, 0.64]
**Subtotal (95% CI)**			**280**			**316**	**84.3%**	**0.48 [0.32, 0.64]**
Heterogeneity: Not applicable								
Test for overall effect: Z = 5.79 (P < 0.00001)								
**1.1.2 Community residents (with sarcopenia)**								
Wei et al. [10]	61.7667	23.5534	60	58.5	20.59	20	8.7%	0.14 [-0.37, 0.65]
**Subtotal (95% CI)**			**60**			**20**	**8.7%**	**0.14 [-0.37, 0.65]**
Heterogeneity: Not applicable								
Test for overall effect: Z = 0.55 (P = 0.58)								
**1.1.3 Institutionalized residents (low functionality)**								
Lam et al. [4]	-1.02	0.52	25	-1.24	0.39	24	6.9%	0.47 [-0.10, 1.04]
**Subtotal (95% CI)**			**25**			**24**	**6.9%**	**0.47 [-0.10, 1.04]**
Heterogeneity: Not applicable								
Test for overall effect: Z = 1.62 (P = 0.11)								
**Total (95% CI)**			**365**			**360**	**100.0%**	**0.45 [0.30, 0.60]**

Heterogeneity: Chi² = 1.57, df = 2 (P = 0.46); I² = 0%
Test for overall effect: Z = 5.90 (P < 0.00001)
Test for subgroup differences: Chi² = 1.57, df = 2 (P = 0.46), I² = 0%

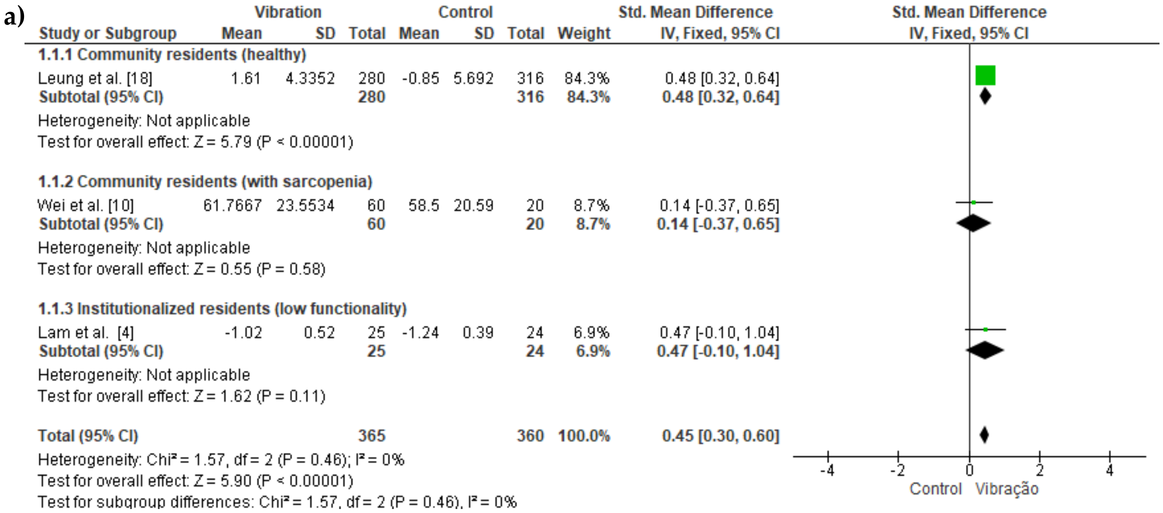

b

Study or Subgroup	Vibration Mean	Vibration SD	Vibration Total	Control Mean	Control SD	Control Total	Weight	Std. Mean Difference IV, Random, 95% CI
**1.3.1 Community residents (healthy)**								
Von Stengel et al. [25]	852.9	193.1	46	796.9	204.2	47	53.8%	0.28 [-0.13, 0.69]
Von Stengel et al. [28]	845.6	225.4	34	688	151.2	33	46.2%	0.81 [0.31, 1.31]
**Subtotal (95% CI)**			**80**			**80**	**100.0%**	**0.52 [0.01, 1.04]**
Heterogeneity: Tau² = 0.09; Chi² = 2.59, df = 1 (P = 0.11); I² = 61%								
Test for overall effect: Z = 1.98 (P = 0.05)								
**Total (95% CI)**			**80**			**80**	**100.0%**	**0.52 [0.01, 1.04]**

Heterogeneity: Tau² = 0.09; Chi² = 2.59, df = 1 (P = 0.11); I² = 61%
Test for overall effect: Z = 1.98 (P = 0.05)
Test for subgroup differences: Not applicable

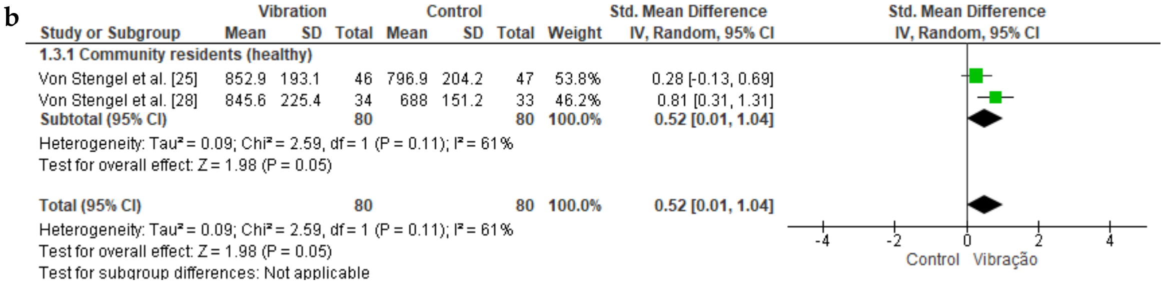

c)

Study or Subgroup	Vibration Mean	Vibration SD	Vibration Total	Control Mean	Control SD	Control Total	Weight	Std. Mean Difference IV, Fixed, 95% CI
**1.4.1 Community residents (healthy)**								
Han et al. [8]	255.89	48.8974	20	213.69	55.03	11	100.0%	0.80 [0.04, 1.57]
**Subtotal (95% CI)**			**20**			**11**	**100.0%**	**0.80 [0.04, 1.57]**
Heterogeneity: Not applicable								
Test for overall effect: Z = 2.06 (P = 0.04)								
**Total (95% CI)**			**20**			**11**	**100.0%**	**0.80 [0.04, 1.57]**

Heterogeneity: Not applicable
Test for overall effect: Z = 2.06 (P = 0.04)
Test for subgroup differences: Not applicable

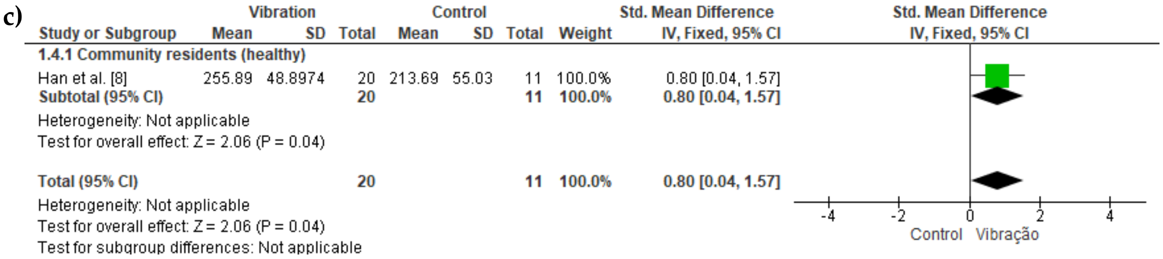

d

Study or Subgroup	Vibration Mean	Vibration SD	Vibration Total	Control Mean	Control SD	Control Total	Weight	Std. Mean Difference IV, Fixed, 95% CI
**1.6.1 Community residents (healthy)**								
Rees et al. [33]	143.8	29.2	15	140.6	27.2	15	35.4%	0.11 [-0.61, 0.83]
**Subtotal (95% CI)**			**15**			**15**	**35.4%**	**0.11 [-0.61, 0.83]**
Heterogeneity: Not applicable								
Test for overall effect: Z = 0.30 (P = 0.76)								
**1.6.2 Community residents (with sarcopenia)**								
Zhu et al. [3]	22.17	5.77	28	20.94	6.33	27	64.6%	0.20 [-0.33, 0.73]
**Subtotal (95% CI)**			**28**			**27**	**64.6%**	**0.20 [-0.33, 0.73]**
Heterogeneity: Not applicable								
Test for overall effect: Z = 0.74 (P = 0.46)								
**Total (95% CI)**			**43**			**42**	**100.0%**	**0.17 [-0.26, 0.59]**

Heterogeneity: Chi² = 0.04, df = 1 (P = 0.84); I² = 0%
Test for overall effect: Z = 0.78 (P = 0.44)
Test for subgroup differences: Chi² = 0.04, df = 1 (P = 0.84), I² = 0%

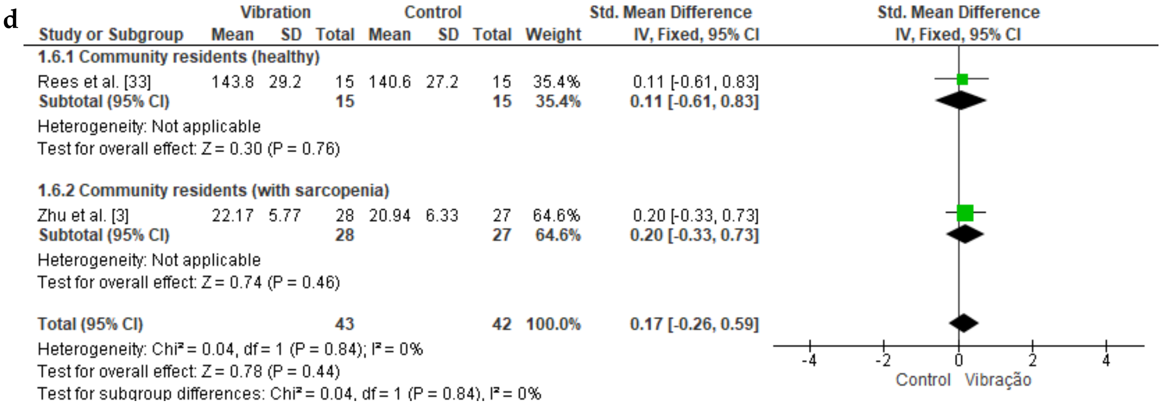

e)

Study or Subgroup	Vibration Mean	Vibration SD	Vibration Total	Control Mean	Control SD	Control Total	Weight	Std. Mean Difference IV, Random, 95% CI
**1.7.1 Community residents (healthy)**								
Pessoa et al. [5]	45.8	2.3	11	39	4.8	10	30.4%	1.76 [0.72, 2.80]
**Subtotal (95% CI)**			**11**			**10**	**30.4%**	**1.76 [0.72, 2.80]**
Heterogeneity: Not applicable								
Test for overall effect: Z = 3.32 (P = 0.0009)								
**1.7.3 Institutionalized residents (healthy)**								
Santin-Medeiros et al. [15]	17.53	5.03	19	18.76	5.83	18	36.3%	-0.22 [-0.87, 0.43]
**Subtotal (95% CI)**			**19**			**18**	**36.3%**	**-0.22 [-0.87, 0.43]**
Heterogeneity: Not applicable								
Test for overall effect: Z = 0.67 (P = 0.50)								
**1.7.4 Institutionalized residents (low functionality)**								
Bautmans et al. [34]	44.6	20.8	10	45.6	25.4	11	33.3%	-0.04 [-0.90, 0.82]
**Subtotal (95% CI)**			**10**			**11**	**33.3%**	**-0.04 [-0.90, 0.82]**
Heterogeneity: Not applicable								
Test for overall effect: Z = 0.09 (P = 0.92)								
**Total (95% CI)**			**40**			**39**	**100.0%**	**0.44 [-0.67, 1.55]**
Heterogeneity: Tau² = 0.78; Chi² = 10.60, df = 2 (P = 0.005); I² = 81%								
Test for overall effect: Z = 0.78 (P = 0.44)								
Test for subgroup differences: Chi² = 10.60, df = 2 (P = 0.005), I² = 81.1%								

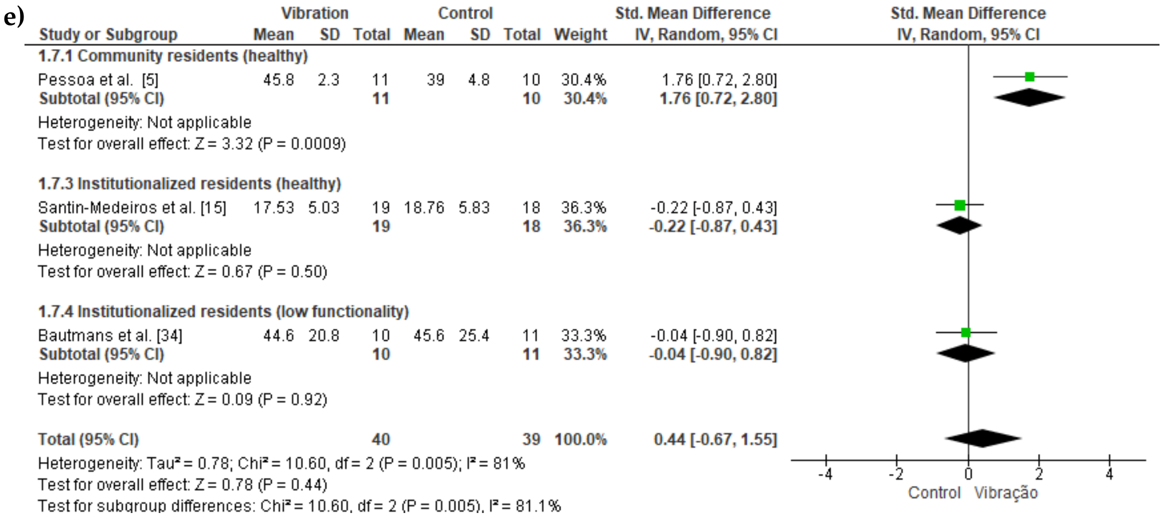

f)

Study or Subgroup	Vibration Mean	Vibration SD	Vibration Total	Control Mean	Control SD	Control Total	Weight	Std. Mean Difference IV, Fixed, 95% CI
**2.1.2 Community residents (with sarcopenia)**								
Wei et al. [9]	11.0867	3.525	60	11.4	2.99	20	24.7%	-0.09 [-0.60, 0.42]
**Subtotal (95% CI)**			**60**			**20**	**24.7%**	**-0.09 [-0.60, 0.42]**
Heterogeneity: Not applicable								
Test for overall effect: Z = 0.35 (P = 0.72)								
**2.1.3 Institutionalized residents (low functionality)**								
Lam et al. [4]	27.3	12.9	25	29	9.8	24	20.1%	-0.15 [-0.71, 0.42]
Sitjà-Rabert et al. [16]	26.68	24.91	59	28.56	27.42	78	55.2%	-0.07 [-0.41, 0.27]
**Subtotal (95% CI)**			**84**			**102**	**75.3%**	**-0.09 [-0.38, 0.20]**
Heterogeneity: Chi² = 0.05, df = 1 (P = 0.82); I² = 0%								
Test for overall effect: Z = 0.61 (P = 0.54)								
**Total (95% CI)**			**144**			**122**	**100.0%**	**-0.09 [-0.34, 0.16]**
Heterogeneity: Chi² = 0.05, df = 2 (P = 0.98); I² = 0%								
Test for overall effect: Z = 0.71 (P = 0.48)								
Test for subgroup differences: Chi² = 0.00, df = 1 (P = 1.00), I² = 0%								

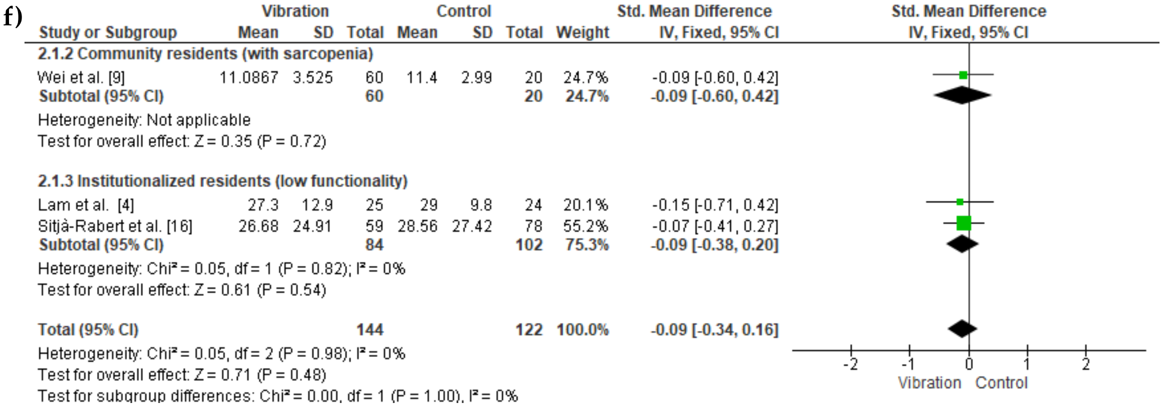

g)

Study or Subgroup	Vibration Mean	Vibration SD	Vibration Total	Control Mean	Control SD	Control Total	Weight	Std. Mean Difference IV, Fixed, 95% CI
**2.2.1 Communit residents (healthy)**								
Von Stengel et al. [28]	14.5	2.3	34	14.5	2.9	33	42.0%	0.00 [-0.48, 0.48]
Von Stengel et al. [25]	23.2	4.3	46	22.5	3.4	47	58.0%	0.18 [-0.23, 0.59]
**Subtotal (95% CI)**			**80**			**80**	**100.0%**	**0.10 [-0.21, 0.41]**
Heterogeneity: Chi² = 0.31, df = 1 (P = 0.58); I² = 0%								
Test for overall effect: Z = 0.66 (P = 0.51)								
**Total (95% CI)**			**80**			**80**	**100.0%**	**0.10 [-0.21, 0.41]**
Heterogeneity: Chi² = 0.31, df = 1 (P = 0.58); I² = 0%								
Test for overall effect: Z = 0.66 (P = 0.51)								
Test for subgroup differences: Not applicable								

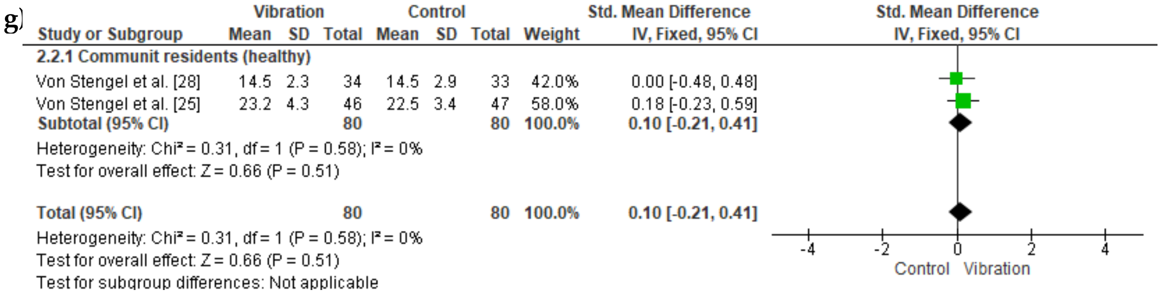

h

Study or Subgroup	Vibration Mean	Vibration SD	Vibration Total	Control Mean	Control SD	Control Total	Weight	Std. Mean Difference IV, Fixed, 95% CI
**3.1.1 Community residents (healthy)**								
Bautmans et al. [34]	8.8	5.4	10	8.3	4	11	14.5%	0.10 [-0.76, 0.96]
Dudoniene et al. [23]	17.4	3.3	20	16.8	2.4	20	27.7%	0.20 [-0.42, 0.83]
Gómez-Cabello et el. [24]	16.5	3.6	24	15.5	2.6	25	33.6%	0.31 [-0.25, 0.88]
**Subtotal (95% CI)**			**54**			**56**	**75.8%**	**0.23 [-0.14, 0.61]**
Heterogeneity: Chi² = 0.18, df = 2 (P = 0.91); I² = 0%								
Test for overall effect: Z = 1.22 (P = 0.22)								
**3.1.2 Institutionalized residents (healthy)**								
Santin-Medeiros et al. [15]	10.17	4.45	19	13.47	5.15	18	24.2%	-0.67 [-1.34, -0.01]
**Subtotal (95% CI)**			**19**			**18**	**24.2%**	**-0.67 [-1.34, -0.01]**
Heterogeneity: Not applicable								
Test for overall effect: Z = 1.98 (P = 0.05)								
**Total (95% CI)**			**73**			**74**	**100.0%**	**0.01 [-0.31, 0.34]**
Heterogeneity: Chi² = 5.58, df = 3 (P = 0.13); I² = 46%								
Test for overall effect: Z = 0.09 (P = 0.93)								
Test for subgroup differences: Chi² = 5.41, df = 1 (P = 0.02), I² = 81.5%								

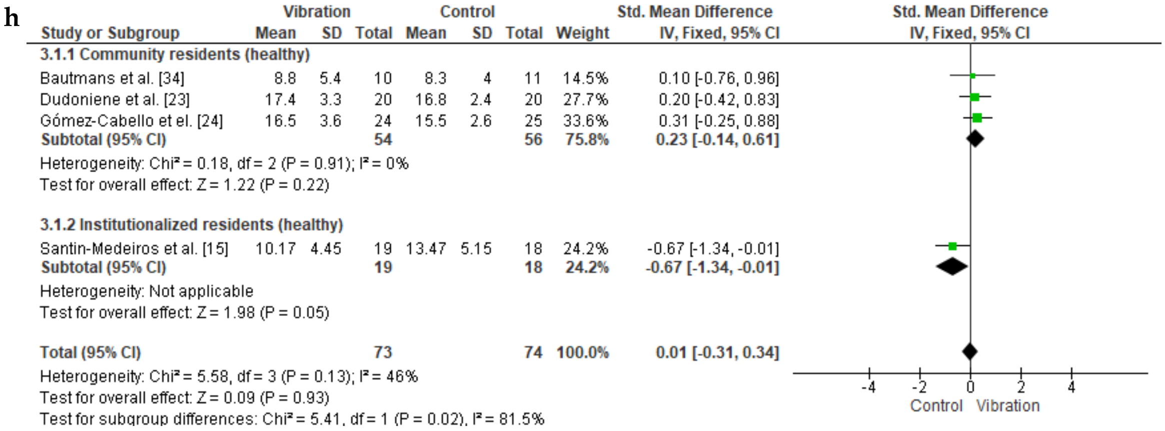

i)

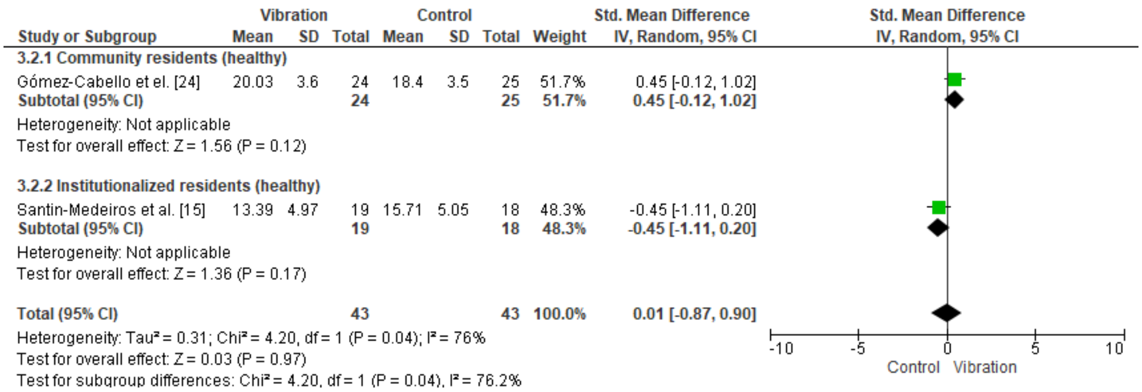

Supplementary Figure S8. Analysis comparing the effectiveness of WBV vs. control groups on synchronous type platforms: a) knee extensor muscle strength; b) muscular strength of the extensors of the lower limbs; c) muscular strength of the plantar flexors; d) muscle strength of the hip flexors; e) handgrip muscle strength; f) five-times-sit-to-stand; g) countermovement jump; h) 30-s sit-to-stand; i) 30-s arm curl.

a)

Study or Subgroup	Vibration Mean	SD	Total	Control Mean	SD	Total	Weight	Std. Mean Difference IV, Random, 95% CI
**1.1.1 Community residents (healthy)**								
Rees et al. [33]	177.8	34.4	15	163.4	38.3	15	16.7%	0.38 [-0.34, 1.11]
Camacho-Cardenosa et al. [2]	97.4071	36.8838	21	76.71	27.77	10	15.4%	0.59 [-0.18, 1.36]
Goudarzian et al. [7]	16.43	4.62	7	12.8	6.41	7	9.3%	0.61 [-0.47, 1.69]
Tseng et al. [12]	22.3206	15.1856	31	1.43	6.59	14	16.9%	1.56 [0.84, 2.27]
**Subtotal (95% CI)**			**74**			**46**	**58.2%**	**0.81 [0.24, 1.37]**
Heterogeneity: $Tau^2 = 0.16$; $Chi^2 = 5.95$, df = 3 (P = 0.11); $I^2 = 50\%$								
Test for overall effect: Z = 2.79 (P = 0.005)								
**1.1.2 Community residents (with sarcopenia)**								
Zhu et al. [3]	19.74	3.92	28	18.15	4.75	27	23.5%	0.36 [-0.17, 0.89]
**Subtotal (95% CI)**			**28**			**27**	**23.5%**	**0.36 [-0.17, 0.89]**
Heterogeneity: Not applicable								
Test for overall effect: Z = 1.33 (P = 0.19)								
**1.1.4 Outpatients (frail)**								
Zhang et al. [21]	11.18	2.03	19	9.76	1.52	18	18.3%	0.77 [0.10, 1.44]
**Subtotal (95% CI)**			**19**			**18**	**18.3%**	**0.77 [0.10, 1.44]**
Heterogeneity: Not applicable								
Test for overall effect: Z = 2.25 (P = 0.02)								
**Total (95% CI)**			**121**			**91**	**100.0%**	**0.70 [0.33, 1.07]**

Heterogeneity: $Tau^2 = 0.08$; $Chi^2 = 7.92$, df = 5 (P = 0.16); $I^2 = 37\%$
Test for overall effect: Z = 3.71 (P = 0.0002)
Test for subgroup differences: $Chi^2 = 1.52$, df = 2 (P = 0.47), $I^2 = 0\%$

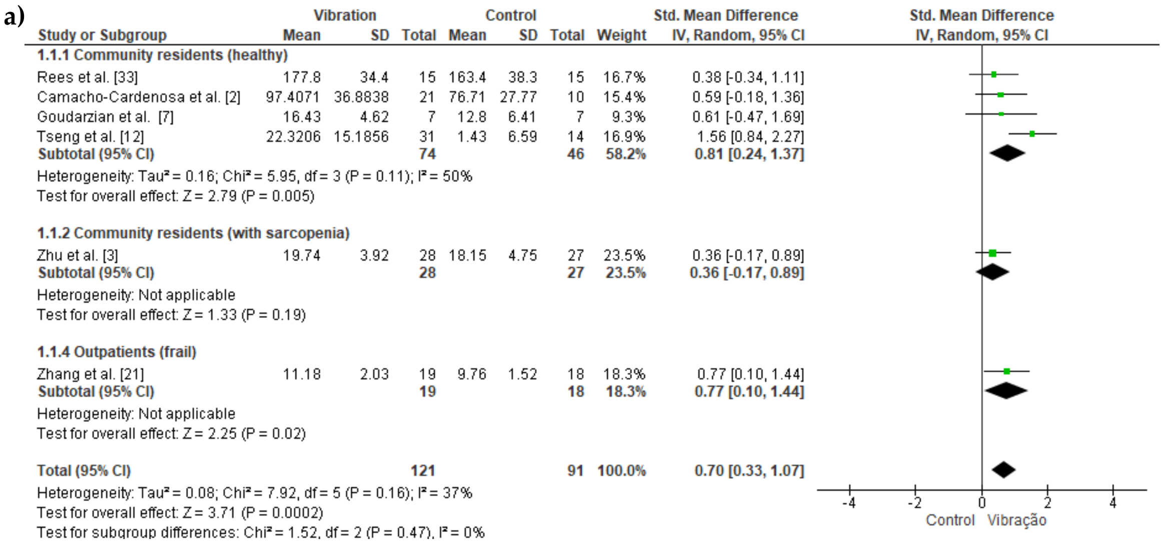

b

Study or Subgroup	Vibration Mean	SD	Total	Control Mean	SD	Total	Weight	Std. Mean Difference IV, Fixed, 95% CI
**1.2.1 Community residents (healthy)**								
Rees et al. [33]	113.6	29.4	15	109	32.4	15	21.1%	0.14 [-0.57, 0.86]
Camacho-Cardenosa et al. [2]	48.6233	20.8587	21	37.84	10.55	10	18.4%	0.57 [-0.19, 1.34]
Tseng et al. [12]	4.5287	7.3548	31	-0.5	6.7	14	25.8%	0.69 [0.04, 1.34]
**Subtotal (95% CI)**			**67**			**39**	**65.2%**	**0.48 [0.07, 0.89]**
Heterogeneity: $Chi^2 = 1.30$, df = 2 (P = 0.52); $I^2 = 0\%$								
Test for overall effect: Z = 2.31 (P = 0.02)								
**1.2.2 Community residents (with sarcopenia)**								
Zhu et al. [3]	23.9	3.42	28	19.47	5.76	27	34.8%	0.93 [0.37, 1.48]
**Subtotal (95% CI)**			**28**			**27**	**34.8%**	**0.93 [0.37, 1.48]**
Heterogeneity: Not applicable								
Test for overall effect: Z = 3.25 (P = 0.001)								
**Total (95% CI)**			**95**			**66**	**100.0%**	**0.64 [0.31, 0.96]**

Heterogeneity: $Chi^2 = 2.89$, df = 3 (P = 0.41); $I^2 = 0\%$
Test for overall effect: Z = 3.78 (P = 0.0002)
Test for subgroup differences: $Chi^2 = 1.59$, df = 1 (P = 0.21), $I^2 = 37.2\%$

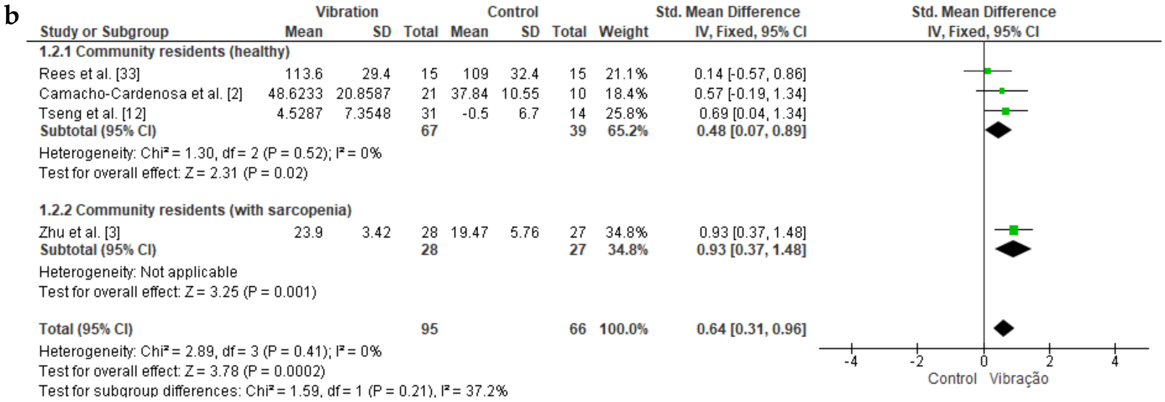

c)

Study or Subgroup	Vibration Mean	SD	Total	Control Mean	SD	Total	Weight	Std. Mean Difference IV, Fixed, 95% CI
**1.3.1 Community residents (healthy)**								
Von Stengel et al. [28]	880.2	253.6	29	688	151.2	33	78.0%	0.92 [0.40, 1.45]
Goudarzian et al. [6]	110.58	12.79	11	88.4	19.39	9	22.0%	1.32 [0.33, 2.32]
**Subtotal (95% CI)**			**40**			**42**	**100.0%**	**1.01 [0.55, 1.48]**
Heterogeneity: $Chi^2 = 0.48$, df = 1 (P = 0.49); $I^2 = 0\%$								
Test for overall effect: Z = 4.26 (P < 0.0001)								
**Total (95% CI)**			**40**			**42**	**100.0%**	**1.01 [0.55, 1.48]**

Heterogeneity: $Chi^2 = 0.48$, df = 1 (P = 0.49); $I^2 = 0\%$
Test for overall effect: Z = 4.26 (P < 0.0001)
Test for subgroup differences: Not applicable

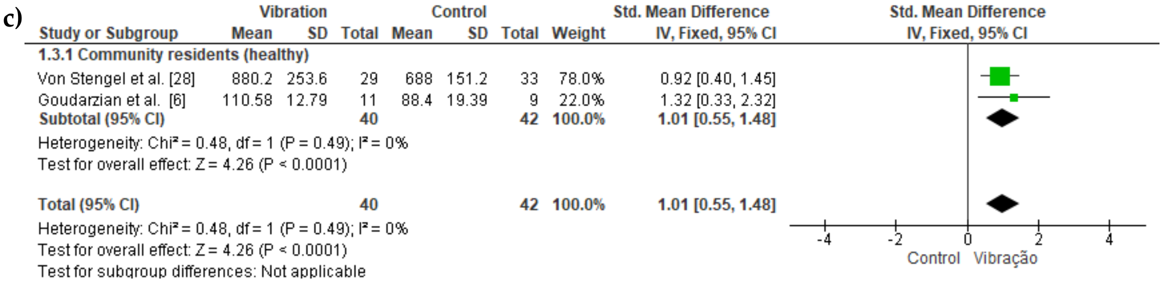

d

Study or Subgroup	Vibration Mean	SD	Total	Control Mean	SD	Total	Weight	Std. Mean Difference IV, Fixed, 95% CI
**1.4.1 Community residents (healthy)**								
Rees et al. [33]	120.4	24.4	15	109.2	18.1	15	100.0%	0.51 [-0.22, 1.24]
**Subtotal (95% CI)**			**15**			**15**	**100.0%**	**0.51 [-0.22, 1.24]**
Heterogeneity: Not applicable								
Test for overall effect: Z = 1.36 (P = 0.17)								
**Total (95% CI)**			**15**			**15**	**100.0%**	**0.51 [-0.22, 1.24]**

Heterogeneity: Not applicable
Test for overall effect: Z = 1.36 (P = 0.17)
Test for subgroup differences: Not applicable

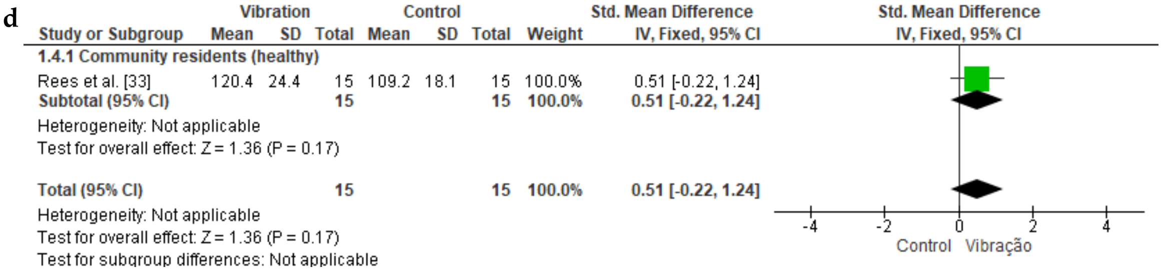

e)

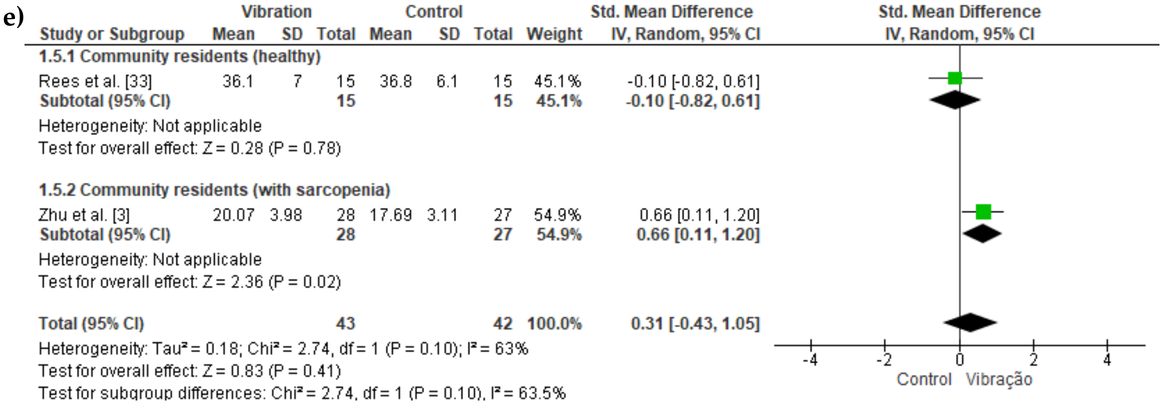
Vibration
Control
Std. Mean Difference
Std. Mean Difference
Study or Subgroup Mean SD Total Mean SD Total Weight IV, Random, 95% CI
IV, Random, 95% CI
1.5.1 Community residents (healthy)
Rees et al. [33] 36.1 7 15 36.8 6.1 15 45.1% -0.10 [-0.82, 0.61]
Subtotal (95% CI) 15 15 45.1% -0.10 [-0.82, 0.61]
Heterogeneity: Not applicable
Test for overall effect: Z = 0.28 (P = 0.78)
1.5.2 Community residents (with sarcopenia)
Zhu et al. [3] 20.07 3.98 28 17.69 3.11 27 54.9% 0.66 [0.11, 1.20]
Subtotal (95% CI) 28 27 54.9% 0.66 [0.11, 1.20]
Heterogeneity: Not applicable
Test for overall effect: Z = 2.36 (P = 0.02)
Total (95% CI) 43 42 100.0% 0.31 [-0.43, 1.05]
Heterogeneity: Tau² = 0.18; Chi² = 2.74, df = 1 (P = 0.10); I² = 63%
Test for overall effect: Z = 0.83 (P = 0.41)
Test for subgroup differences: Chi² = 2.74, df = 1 (P = 0.10), I² = 63.5%
-4 -2 0 2 4
Control Vibração

f)

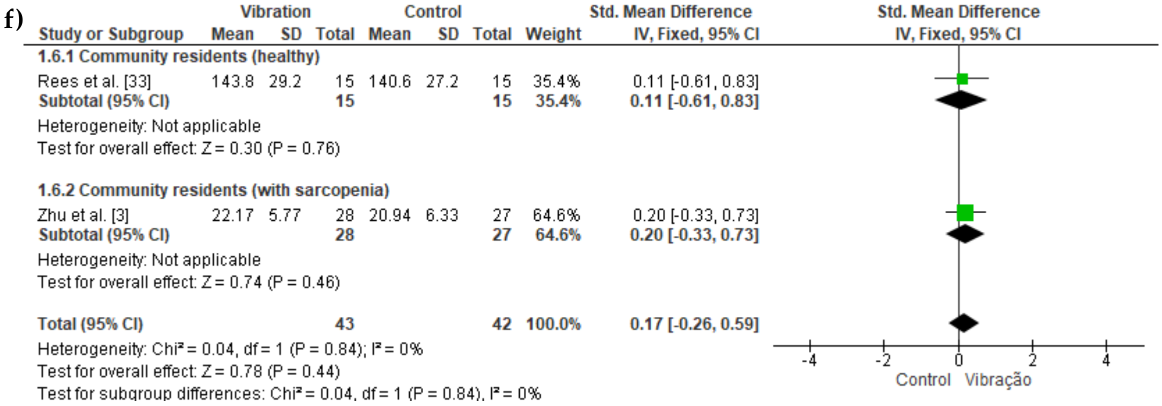
Vibration
Control
Std. Mean Difference
Std. Mean Difference
Study or Subgroup Mean SD Total Mean SD Total Weight IV, Fixed, 95% CI
IV, Fixed, 95% CI
1.6.1 Community residents (healthy)
Rees et al. [33] 143.8 29.2 15 140.6 27.2 15 35.4% 0.11 [-0.61, 0.83]
Subtotal (95% CI) 15 15 35.4% 0.11 [-0.61, 0.83]
Heterogeneity: Not applicable
Test for overall effect: Z = 0.30 (P = 0.76)
1.6.2 Community residents (with sarcopenia)
Zhu et al. [3] 22.17 5.77 28 20.94 6.33 27 64.6% 0.20 [-0.33, 0.73]
Subtotal (95% CI) 28 27 64.6% 0.20 [-0.33, 0.73]
Heterogeneity: Not applicable
Test for overall effect: Z = 0.74 (P = 0.46)
Total (95% CI) 43 42 100.0% 0.17 [-0.26, 0.59]
Heterogeneity: Chi² = 0.04, df = 1 (P = 0.84); I² = 0%
Test for overall effect: Z = 0.78 (P = 0.44)
Test for subgroup differences: Chi² = 0.04, df = 1 (P = 0.84), I² = 0%
-4 -2 0 2 4
Control Vibração

g)

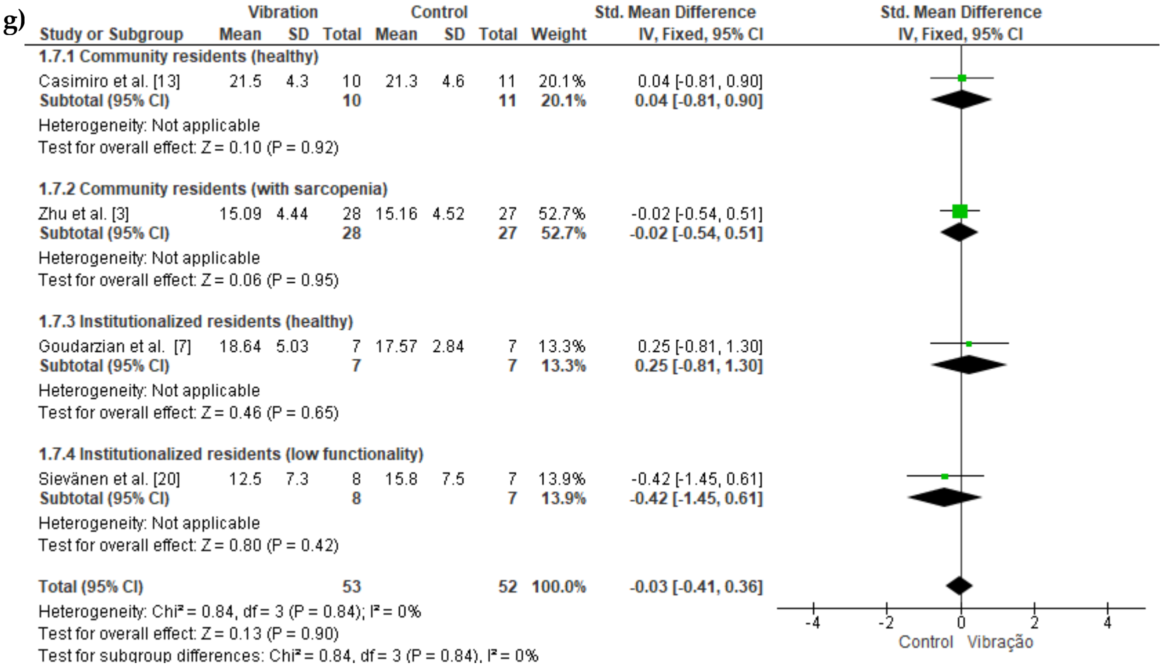
Vibration
Control
Std. Mean Difference
Std. Mean Difference
Study or Subgroup Mean SD Total Mean SD Total Weight IV, Fixed, 95% CI
IV, Fixed, 95% CI
1.7.1 Community residents (healthy)
Casimiro et al. [13] 21.5 4.3 10 21.3 4.6 11 20.1% 0.04 [-0.81, 0.90]
Subtotal (95% CI) 10 11 20.1% 0.04 [-0.81, 0.90]
Heterogeneity: Not applicable
Test for overall effect: Z = 0.10 (P = 0.92)
1.7.2 Community residents (with sarcopenia)
Zhu et al. [3] 15.09 4.44 28 15.16 4.52 27 52.7% -0.02 [-0.54, 0.51]
Subtotal (95% CI) 28 27 52.7% -0.02 [-0.54, 0.51]
Heterogeneity: Not applicable
Test for overall effect: Z = 0.06 (P = 0.95)
1.7.3 Institutionalized residents (healthy)
Goudarzian et al. [7] 18.64 5.03 7 17.57 2.84 7 13.3% 0.25 [-0.81, 1.30]
Subtotal (95% CI) 7 7 13.3% 0.25 [-0.81, 1.30]
Heterogeneity: Not applicable
Test for overall effect: Z = 0.46 (P = 0.65)
1.7.4 Institutionalized residents (low functionality)
Sievänen et al. [20] 12.5 7.3 8 15.8 7.5 7 13.9% -0.42 [-1.45, 0.61]
Subtotal (95% CI) 8 7 13.9% -0.42 [-1.45, 0.61]
Heterogeneity: Not applicable
Test for overall effect: Z = 0.80 (P = 0.42)
Total (95% CI) 53 52 100.0% -0.03 [-0.41, 0.36]
Heterogeneity: Chi² = 0.84, df = 3 (P = 0.84); I² = 0%
Test for overall effect: Z = 0.13 (P = 0.90)
Test for subgroup differences: Chi² = 0.84, df = 3 (P = 0.84), I² = 0%
-4 -2 0 2 4
Control Vibração

h

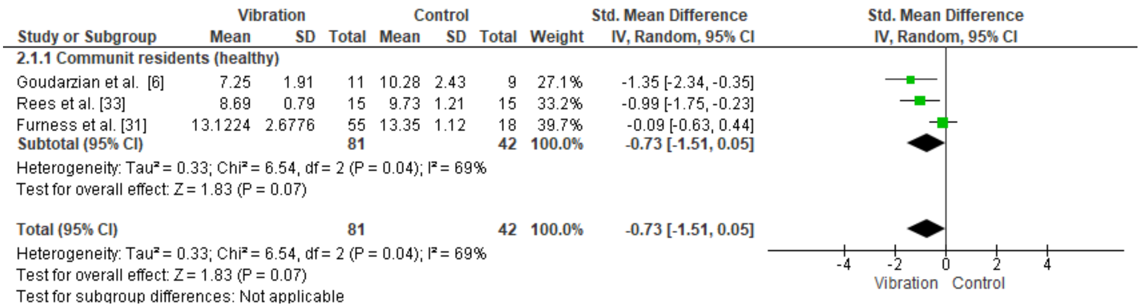

i)

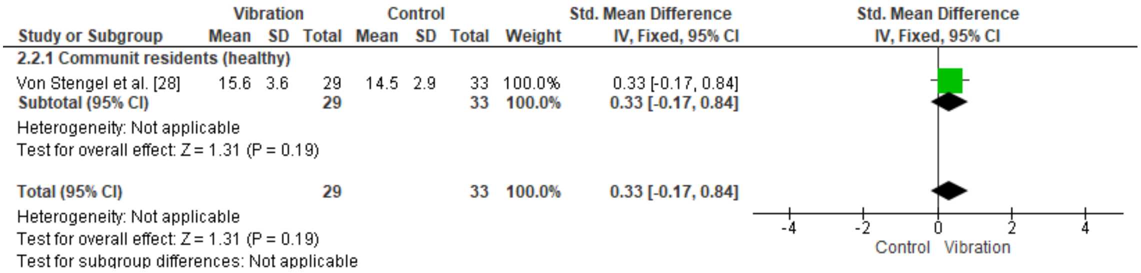

j)

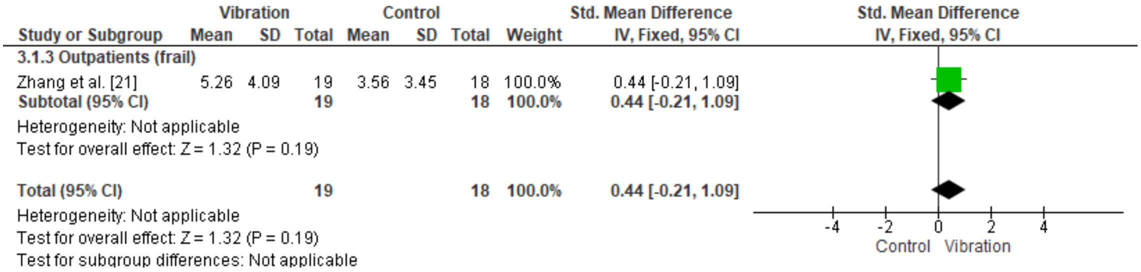

Supplementary Figure S9. Analysis comparing the effectiveness of WBV vs. control groups on side-alternating platforms: a) muscle strength of the knee extensors; b) muscle strength of the knee flexors; c) muscular strength of the extensors of the lower limbs; d) muscular strength of ankle plantar flexors; e) muscular strength of the ankle dorsiflexors; f) muscle strength of the hip flexors; g) handgrip muscle strength; h) five-times-sit-to-stand; i) countermovement jump; j) 30-s sit-to-stand.

a)

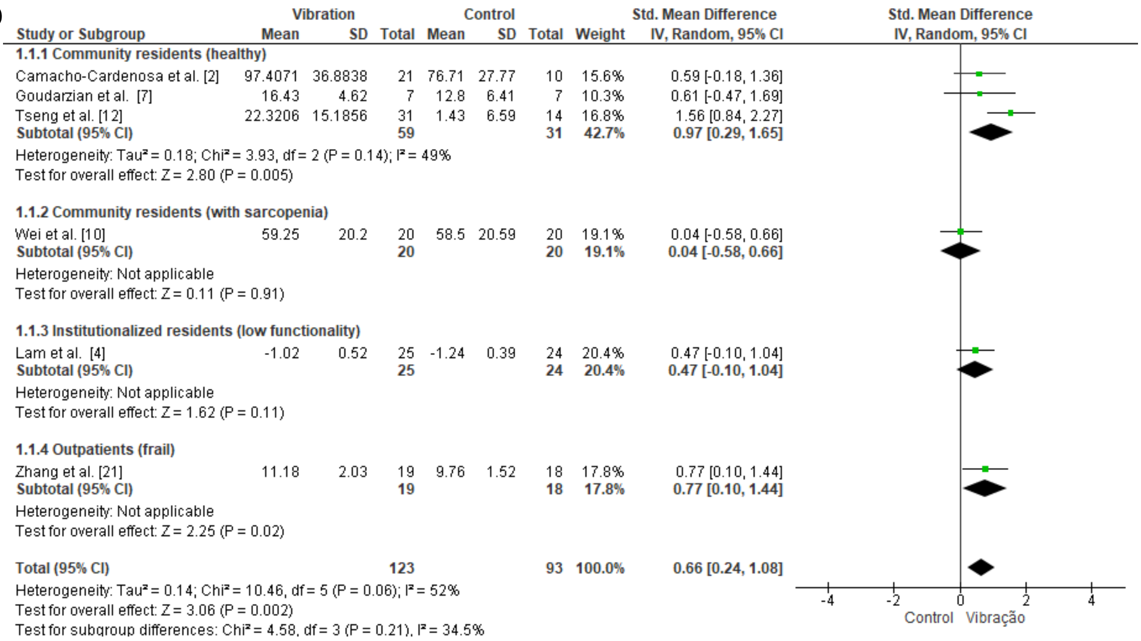
Vibration
Control
Std. Mean Difference
Std. Mean Difference
Study or Subgroup Mean SD Total Mean SD Total Weight IV, Random, 95% CI
IV, Random, 95% CI
1.1.1 Community residents (healthy)
Camacho-Cardenosa et al. [2] 97.4071 36.8838 21 76.71 27.77 10 15.6% 0.59 [-0.18, 1.36]
Goudarzian et al. [7] 16.43 4.62 7 12.8 6.41 7 10.3% 0.61 [-0.47, 1.69]
Tseng et al. [12] 22.3206 15.1856 31 1.43 6.59 14 16.8% 1.56 [0.84, 2.27]
Subtotal (95% CI) 59 31 42.7% 0.97 [0.29, 1.65]
Heterogeneity: Tau² = 0.18; Chi² = 3.93, df = 2 (P = 0.14); I² = 49%
Test for overall effect: Z = 2.80 (P = 0.005)
1.1.2 Community residents (with sarcopenia)
Wei et al. [10] 59.25 20.2 20 58.5 20.59 20 19.1% 0.04 [-0.58, 0.66]
Subtotal (95% CI) 20 20 19.1% 0.04 [-0.58, 0.66]
Heterogeneity: Not applicable
Test for overall effect: Z = 0.11 (P = 0.91)
1.1.3 Institutionalized residents (low functionality)
Lam et al. [4] -1.02 0.52 25 -1.24 0.39 24 20.4% 0.47 [-0.10, 1.04]
Subtotal (95% CI) 25 24 20.4% 0.47 [-0.10, 1.04]
Heterogeneity: Not applicable
Test for overall effect: Z = 1.62 (P = 0.11)
1.1.4 Outpatients (frail)
Zhang et al. [21] 11.18 2.03 19 9.76 1.52 18 17.8% 0.77 [0.10, 1.44]
Subtotal (95% CI) 19 18 17.8% 0.77 [0.10, 1.44]
Heterogeneity: Not applicable
Test for overall effect: Z = 2.25 (P = 0.02)
Total (95% CI) 123 93 100.0% 0.66 [0.24, 1.08]
Heterogeneity: Tau² = 0.14; Chi² = 10.46, df = 5 (P = 0.06); I² = 52%
Test for overall effect: Z = 3.06 (P = 0.002)
Test for subgroup differences: Chi² = 4.58, df = 3 (P = 0.21), I² = 34.5%
-4 -2 0 2 4
Control Vibração

b

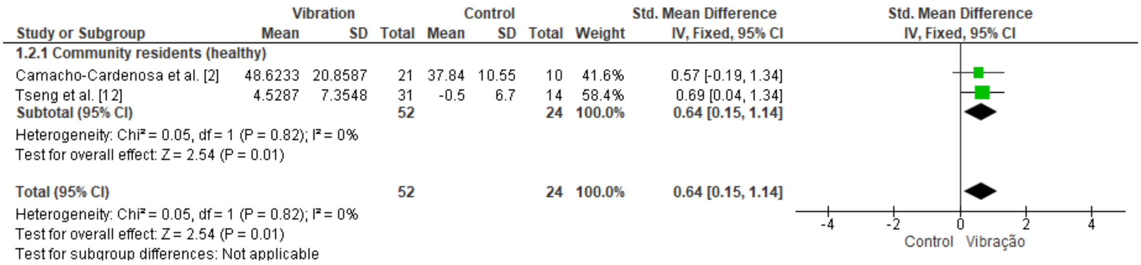
Vibration
Control
Std. Mean Difference
Std. Mean Difference
Study or Subgroup Mean SD Total Mean SD Total Weight IV, Fixed, 95% CI
IV, Fixed, 95% CI
1.2.1 Community residents (healthy)
Camacho-Cardenosa et al. [2] 48.6233 20.8587 21 37.84 10.55 10 41.6% 0.57 [-0.19, 1.34]
Tseng et al. [12] 4.5287 7.3548 31 -0.5 6.7 14 58.4% 0.69 [0.04, 1.34]
Subtotal (95% CI) 52 24 100.0% 0.64 [0.15, 1.14]
Heterogeneity: Chi² = 0.05, df = 1 (P = 0.82); I² = 0%
Test for overall effect: Z = 2.54 (P = 0.01)
Total (95% CI) 52 24 100.0% 0.64 [0.15, 1.14]
Heterogeneity: Chi² = 0.05, df = 1 (P = 0.82); I² = 0%
Test for overall effect: Z = 2.54 (P = 0.01)
Test for subgroup differences: Not applicable
-4 -2 0 2 4
Control Vibração

c)

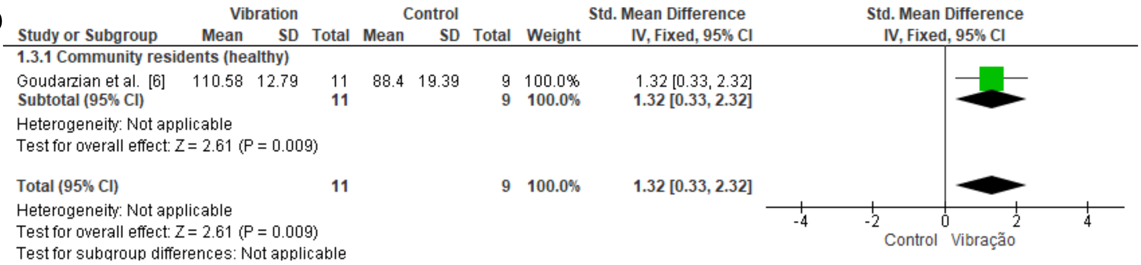
Vibration
Control
Std. Mean Difference
Std. Mean Difference
Study or Subgroup Mean SD Total Mean SD Total Weight IV, Fixed, 95% CI
IV, Fixed, 95% CI
1.3.1 Community residents (healthy)
Goudarzian et al. [6] 110.58 12.79 11 88.4 19.39 9 100.0% 1.32 [0.33, 2.32]
Subtotal (95% CI) 11 9 100.0% 1.32 [0.33, 2.32]
Heterogeneity: Not applicable
Test for overall effect: Z = 2.61 (P = 0.009)
Total (95% CI) 11 9 100.0% 1.32 [0.33, 2.32]
Heterogeneity: Not applicable
Test for overall effect: Z = 2.61 (P = 0.009)
Test for subgroup differences: Not applicable
-4 -2 0 2 4
Control Vibração

d

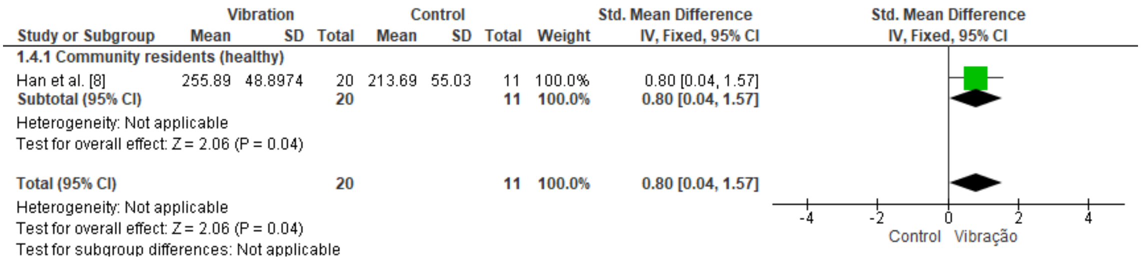
Vibration
Control
Std. Mean Difference
Std. Mean Difference
Study or Subgroup Mean SD Total Mean SD Total Weight IV, Fixed, 95% CI
IV, Fixed, 95% CI
1.4.1 Community residents (healthy)
Han et al. [8] 255.89 48.8974 20 213.69 55.03 11 100.0% 0.80 [0.04, 1.57]
Subtotal (95% CI) 20 11 100.0% 0.80 [0.04, 1.57]
Heterogeneity: Not applicable
Test for overall effect: Z = 2.06 (P = 0.04)
Total (95% CI) 20 11 100.0% 0.80 [0.04, 1.57]
Heterogeneity: Not applicable
Test for overall effect: Z = 2.06 (P = 0.04)
Test for subgroup differences: Not applicable
-4 -2 0 2 4
Control Vibração

e)

Study or Subgroup	Vibration Mean	Vibration SD	Vibration Total	Control Mean	Control SD	Control Total	Weight	Std. Mean Difference IV, Fixed, 95% CI
**1.7.3 Institutionalized residents (healthy)**								
Goudarzian et al. [7]	18.64	5.03	7	17.57	2.84	7	28.1%	0.25 [-0.81, 1.30]
**Subtotal (95% CI)**			**7**			**7**	**28.1%**	**0.25 [-0.81, 1.30]**
Heterogeneity: Not applicable								
Test for overall effect: Z = 0.46 (P = 0.65)								
**1.7.4 Institutionalized residents (low functionality)**								
Sievänen et al. [20]	12.5	7.3	8	15.8	7.5	7	29.4%	-0.42 [-1.45, 0.61]
Bautmans et al. [34]	44.6	20.8	10	45.6	25.4	11	42.5%	-0.04 [-0.90, 0.82]
**Subtotal (95% CI)**			**18**			**18**	**71.9%**	**-0.20 [-0.85, 0.46]**
Heterogeneity: Chi² = 0.31, df = 1 (P = 0.58); I² = 0%								
Test for overall effect: Z = 0.58 (P = 0.56)								
**Total (95% CI)**			**25**			**25**	**100.0%**	**-0.07 [-0.63, 0.49]**
Heterogeneity: Chi² = 0.79, df = 2 (P = 0.67); I² = 0%								
Test for overall effect: Z = 0.25 (P = 0.80)								
Test for subgroup differences: Chi² = 0.49, df = 1 (P = 0.49), I² = 0%								

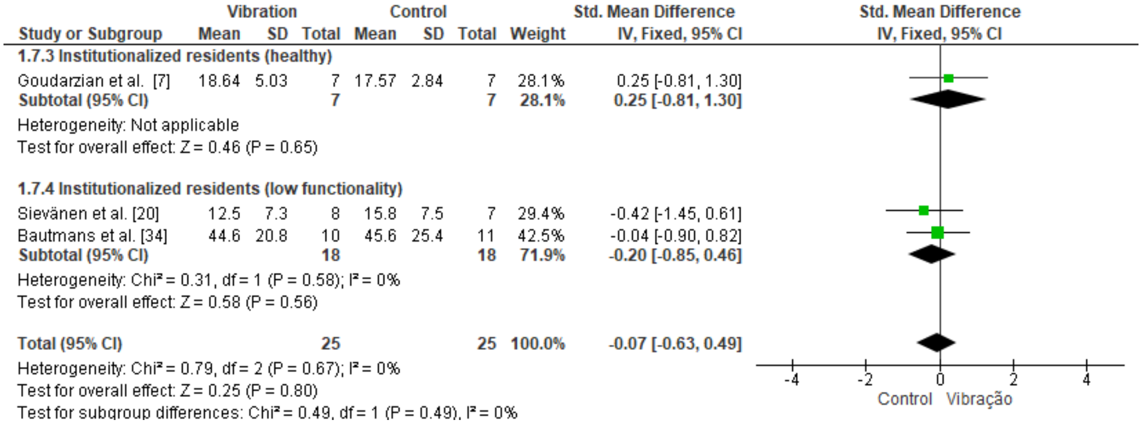

f)

Study or Subgroup	Vibration Mean	Vibration SD	Vibration Total	Control Mean	Control SD	Control Total	Weight	Std. Mean Difference IV, Random, 95% CI
**2.1.1 Communit residents (healthy)**								
Goudarzian et al. [6]	7.25	1.91	11	10.28	2.43	9	7.7%	-1.35 [-2.34, -0.35]
Furness et al. [31]	13.1224	2.6776	55	13.35	1.12	18	20.9%	-0.09 [-0.63, 0.44]
**Subtotal (95% CI)**			**66**			**27**	**28.6%**	**-0.65 [-1.86, 0.57]**
Heterogeneity: Tau² = 0.62; Chi² = 4.72, df = 1 (P = 0.03); I² = 79%								
Test for overall effect: Z = 1.04 (P = 0.30)								
**2.1.2 Community residents (with sarcopenia)**								
Wei et al. [9]	11.05	2.2	20	11.4	2.99	20	16.8%	-0.13 [-0.75, 0.49]
**Subtotal (95% CI)**			**20**			**20**	**16.8%**	**-0.13 [-0.75, 0.49]**
Heterogeneity: Not applicable								
Test for overall effect: Z = 0.41 (P = 0.68)								
**2.1.3 Institutionalized residents (low functionality)**								
Lam et al. [4]	27.3	12.9	25	29	9.8	24	19.4%	-0.15 [-0.71, 0.42]
Sitjà-Rabert et al. [16]	26.68	24.91	59	28.56	27.42	78	35.2%	-0.07 [-0.41, 0.27]
**Subtotal (95% CI)**			**84**			**102**	**54.6%**	**-0.09 [-0.38, 0.20]**
Heterogeneity: Tau² = 0.00; Chi² = 0.05, df = 1 (P = 0.82); I² = 0%								
Test for overall effect: Z = 0.61 (P = 0.54)								
**Total (95% CI)**			**170**			**149**	**100.0%**	**-0.20 [-0.49, 0.10]**
Heterogeneity: Tau² = 0.03; Chi² = 5.78, df = 4 (P = 0.22); I² = 31%								
Test for overall effect: Z = 1.32 (P = 0.19)								
Test for subgroup differences: Chi² = 0.76, df = 2 (P = 0.69), I² = 0%								

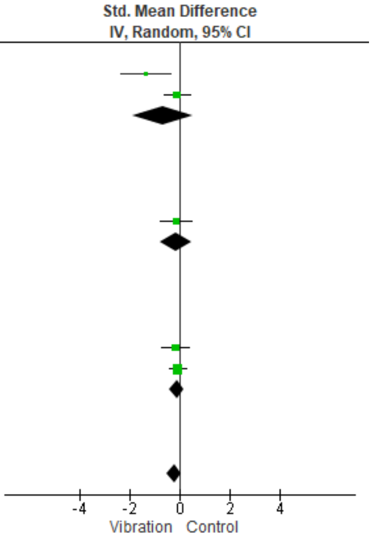

g)

Study or Subgroup	Vibration Mean	Vibration SD	Vibration Total	Control Mean	Control SD	Control Total	Weight	Std. Mean Difference IV, Fixed, 95% CI
**3.1.1 Community residents (healthy)**								
Bautmans et al. [34]	8.8	5.4	10	8.3	4	11	11.6%	0.10 [-0.76, 0.96]
Dudoniene et al. [23]	17.4	3.3	20	16.8	2.4	20	22.1%	0.20 [-0.42, 0.83]
Gómez-Cabello et el. [24]	16.5	3.6	24	15.5	2.6	25	26.9%	0.31 [-0.25, 0.88]
**Subtotal (95% CI)**			**54**			**56**	**60.6%**	**0.23 [-0.14, 0.61]**
Heterogeneity: Chi² = 0.18, df = 2 (P = 0.91); I² = 0%								
Test for overall effect: Z = 1.22 (P = 0.22)								
**3.1.2 Institutionalized residents (healthy)**								
Santin-Medeiros et al. [15]	10.17	4.45	19	13.47	5.15	18	19.3%	-0.67 [-1.34, -0.01]
**Subtotal (95% CI)**			**19**			**18**	**19.3%**	**-0.67 [-1.34, -0.01]**
Heterogeneity: Not applicable								
Test for overall effect: Z = 1.98 (P = 0.05)								
**3.1.3 Outpatients (frail)**								
Zhang et al. [21]	5.26	4.09	19	3.56	3.45	18	20.0%	0.44 [-0.21, 1.09]
**Subtotal (95% CI)**			**19**			**18**	**20.0%**	**0.44 [-0.21, 1.09]**
Heterogeneity: Not applicable								
Test for overall effect: Z = 1.32 (P = 0.19)								
**Total (95% CI)**			**92**			**92**	**100.0%**	**0.10 [-0.19, 0.39]**
Heterogeneity: Chi² = 6.88, df = 4 (P = 0.14); I² = 42%								
Test for overall effect: Z = 0.67 (P = 0.51)								
Test for subgroup differences: Chi² = 6.70, df = 2 (P = 0.04), I² = 70.2%								

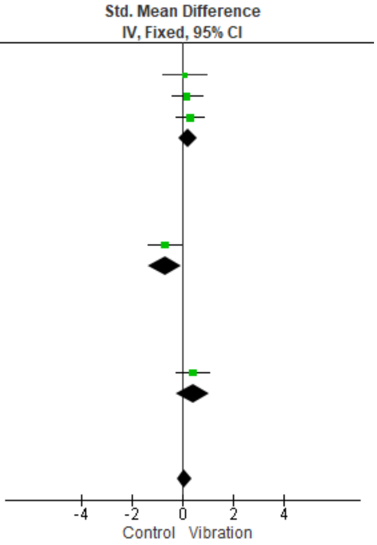

h

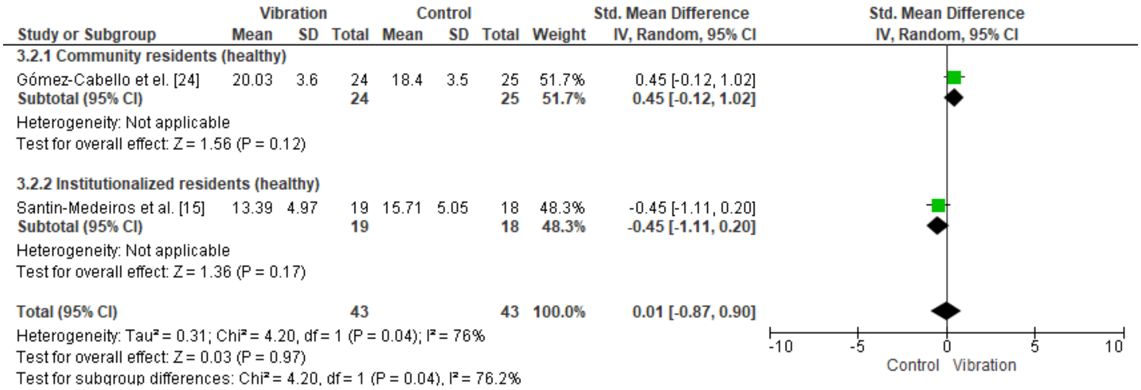

Supplementary Figure S10. Analysis comparing the effectiveness of WBV administered at a low cumulative dose (≤ 144 minutes) vs. control groups: a) muscle strength of the knee extensors; b) muscle strength of the knee flexors; c) muscular strength of the extensors of the lower limbs; d) muscular strength of the plantar flexors; e) handgrip muscle strength; f) five-times-sit-to-stand; g) 30-s sit-to-stand; h) 30-s arm curl.

a)

Study or Subgroup	Vibration Mean	Vibration SD	Vibration Total	Control Mean	Control SD	Control Total	Weight	Std. Mean Difference IV, Fixed, 95% CI
**1.1.1 Community residents (healthy)**								
Rees et al. [33]	177.8	34.4	15	163.4	38.3	15	4.1%	0.38 [-0.34, 1.11]
Leung et al. [18]	1.61	4.3352	280	-0.85	5.692	316	80.9%	0.48 [0.32, 0.64]
**Subtotal (95% CI)**			**295**			**331**	**85.0%**	**0.48 [0.32, 0.64]**
Heterogeneity: Chi² = 0.07, df = 1 (P = 0.80); I² = 0%								
Test for overall effect: Z = 5.87 (P < 0.00001)								
**1.1.2 Community residents (with sarcopenia)**								
Wei et al. [10]	63.025	25.211	40	58.5	20.59	20	7.4%	0.19 [-0.35, 0.73]
Zhu et al. [3]	19.74	3.92	28	18.15	4.75	27	7.6%	0.36 [-0.17, 0.89]
**Subtotal (95% CI)**			**68**			**47**	**15.0%**	**0.27 [-0.10, 0.65]**
Heterogeneity: Chi² = 0.20, df = 1 (P = 0.65); I² = 0%								
Test for overall effect: Z = 1.42 (P = 0.15)								
**Total (95% CI)**			**363**			**378**	**100.0%**	**0.45 [0.30, 0.59]**
Heterogeneity: Chi² = 1.20, df = 3 (P = 0.75); I² = 0%								
Test for overall effect: Z = 5.97 (P < 0.00001)								
Test for subgroup differences: Chi² = 0.93, df = 1 (P = 0.33), I² = 0%								

Std. Mean Difference IV, Fixed, 95% CI

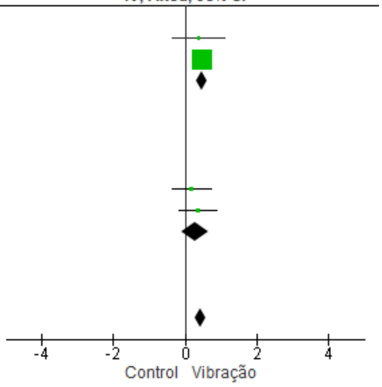

b

Study or Subgroup	Vibration Mean	Vibration SD	Vibration Total	Control Mean	Control SD	Control Total	Weight	Std. Mean Difference IV, Random, 95% CI
**1.2.1 Community residents (healthy)**								
Rees et al. [33]	113.6	29.4	15	109	32.4	15	45.7%	0.14 [-0.57, 0.86]
**Subtotal (95% CI)**			**15**			**15**	**45.7%**	**0.14 [-0.57, 0.86]**
Heterogeneity: Not applicable								
Test for overall effect: Z = 0.40 (P = 0.69)								
**1.2.2 Community residents (with sarcopenia)**								
Zhu et al. [3]	23.9	3.42	28	19.47	5.76	27	54.3%	0.93 [0.37, 1.48]
**Subtotal (95% CI)**			**28**			**27**	**54.3%**	**0.93 [0.37, 1.48]**
Heterogeneity: Not applicable								
Test for overall effect: Z = 3.25 (P = 0.001)								
**Total (95% CI)**			**43**			**42**	**100.0%**	**0.57 [-0.19, 1.33]**
Heterogeneity: Tau² = 0.20; Chi² = 2.84, df = 1 (P = 0.09); I² = 65%								
Test for overall effect: Z = 1.46 (P = 0.14)								
Test for subgroup differences: Chi² = 2.84, df = 1 (P = 0.09), I² = 64.8%								

Std. Mean Difference IV, Random, 95% CI

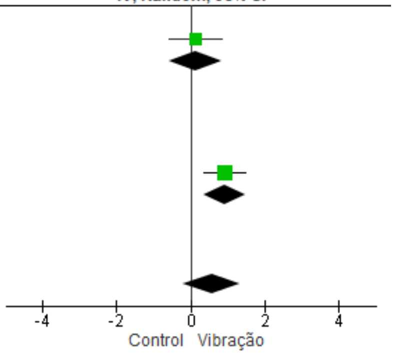

c)

Study or Subgroup	Vibration Mean	Vibration SD	Vibration Total	Control Mean	Control SD	Control Total	Weight	Std. Mean Difference IV, Random, 95% CI
**1.3.1 Community residents (healthy)**								
Von Stengel et al. [25]	852.9	193.1	46	796.9	204.2	47	51.1%	0.28 [-0.13, 0.69]
Von Stengel et al. [28]	861.527	237.462	63	688	151.2	33	48.9%	0.81 [0.37, 1.25]
**Subtotal (95% CI)**			**109**			**80**	**100.0%**	**0.54 [0.02, 1.06]**
Heterogeneity: Tau² = 0.10; Chi² = 3.04, df = 1 (P = 0.08); I² = 67%								
Test for overall effect: Z = 2.03 (P = 0.04)								
**Total (95% CI)**			**109**			**80**	**100.0%**	**0.54 [0.02, 1.06]**
Heterogeneity: Tau² = 0.10; Chi² = 3.04, df = 1 (P = 0.08); I² = 67%								
Test for overall effect: Z = 2.03 (P = 0.04)								
Test for subgroup differences: Not applicable								

Std. Mean Difference IV, Random, 95% CI

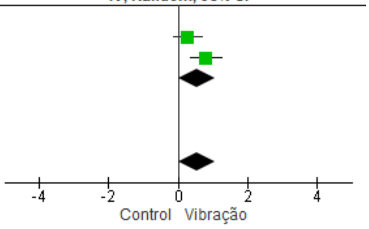

d

Study or Subgroup	Vibration Mean	Vibration SD	Vibration Total	Control Mean	Control SD	Control Total	Weight	Std. Mean Difference IV, Fixed, 95% CI
**1.4.1 Community residents (healthy)**								
Rees et al. [33]	120.4	24.4	15	109.2	18.1	15	100.0%	0.51 [-0.22, 1.24]
**Subtotal (95% CI)**			**15**			**15**	**100.0%**	**0.51 [-0.22, 1.24]**
Heterogeneity: Not applicable								
Test for overall effect: Z = 1.36 (P = 0.17)								
**Total (95% CI)**			**15**			**15**	**100.0%**	**0.51 [-0.22, 1.24]**
Heterogeneity: Not applicable								
Test for overall effect: Z = 1.36 (P = 0.17)								
Test for subgroup differences: Not applicable								

Std. Mean Difference IV, Fixed, 95% CI

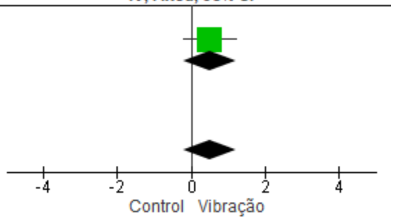

e)

Study or Subgroup	Vibration Mean	Vibration SD	Vibration Total	Control Mean	Control SD	Control Total	Weight	Std. Mean Difference IV, Random, 95% CI
**1.5.1 Community residents (healthy)**								
Rees et al. [33]	36.1	7	15	36.8	6.1	15	45.1%	-0.10 [-0.82, 0.61]
**Subtotal (95% CI)**			**15**			**15**	**45.1%**	**-0.10 [-0.82, 0.61]**
Heterogeneity: Not applicable								
Test for overall effect: Z = 0.28 (P = 0.78)								
**1.5.2 Community residents (with sarcopenia)**								
Zhu et al. [3]	20.07	3.98	28	17.69	3.11	27	54.9%	0.66 [0.11, 1.20]
**Subtotal (95% CI)**			**28**			**27**	**54.9%**	**0.66 [0.11, 1.20]**
Heterogeneity: Not applicable								
Test for overall effect: Z = 2.36 (P = 0.02)								
**Total (95% CI)**			**43**			**42**	**100.0%**	**0.31 [-0.43, 1.05]**
Heterogeneity: Tau² = 0.18; Chi² = 2.74, df = 1 (P = 0.10); I² = 63%								
Test for overall effect: Z = 0.83 (P = 0.41)								
Test for subgroup differences: Chi² = 2.74, df = 1 (P = 0.10), I² = 63.5%								

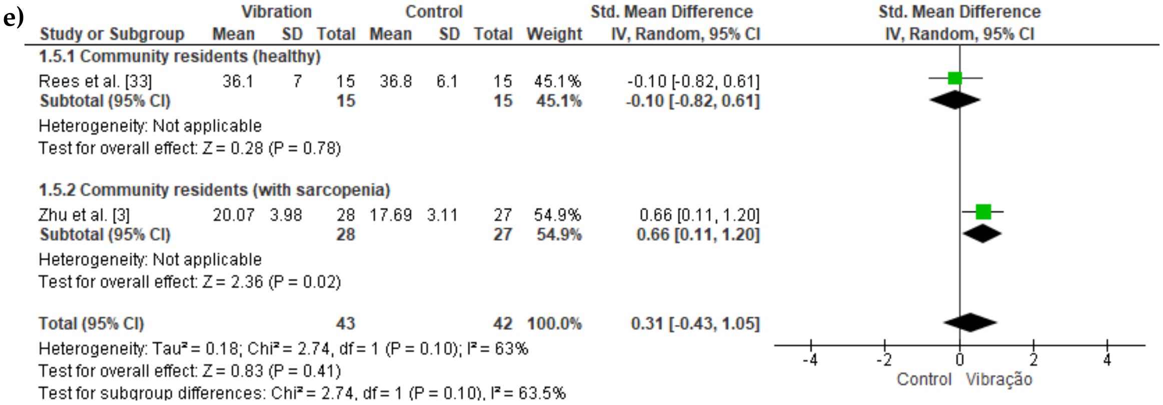

f)

Study or Subgroup	Vibration Mean	Vibration SD	Vibration Total	Control Mean	Control SD	Control Total	Weight	Std. Mean Difference IV, Fixed, 95% CI
**1.6.1 Community residents (healthy)**								
Rees et al. [33]	143.8	29.2	15	140.6	27.2	15	35.4%	0.11 [-0.61, 0.83]
**Subtotal (95% CI)**			**15**			**15**	**35.4%**	**0.11 [-0.61, 0.83]**
Heterogeneity: Not applicable								
Test for overall effect: Z = 0.30 (P = 0.76)								
**1.6.2 Community residents (with sarcopenia)**								
Zhu et al. [3]	22.17	5.77	28	20.94	6.33	27	64.6%	0.20 [-0.33, 0.73]
**Subtotal (95% CI)**			**28**			**27**	**64.6%**	**0.20 [-0.33, 0.73]**
Heterogeneity: Not applicable								
Test for overall effect: Z = 0.74 (P = 0.46)								
**Total (95% CI)**			**43**			**42**	**100.0%**	**0.17 [-0.26, 0.59]**
Heterogeneity: Chi² = 0.04, df = 1 (P = 0.84); I² = 0%								
Test for overall effect: Z = 0.78 (P = 0.44)								
Test for subgroup differences: Chi² = 0.04, df = 1 (P = 0.84), I² = 0%								

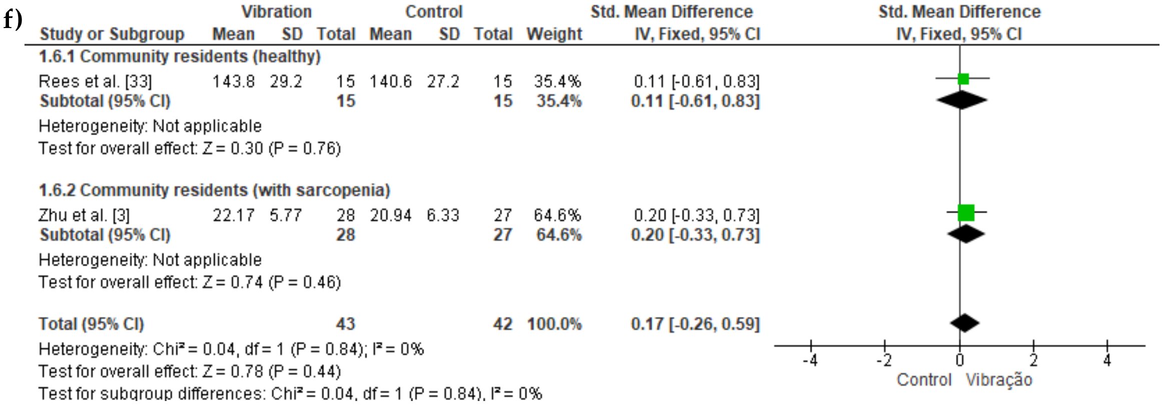

g)

Study or Subgroup	Vibration Mean	Vibration SD	Vibration Total	Control Mean	Control SD	Control Total	Weight	Std. Mean Difference IV, Random, 95% CI
**1.7.1 Community residents (healthy)**								
Casimiro et al. [13]	21.5	4.3	10	21.3	4.6	11	23.2%	0.04 [-0.81, 0.90]
Pessoa et al. [5]	45.8	2.3	11	39	4.8	10	20.0%	1.76 [0.72, 2.80]
**Subtotal (95% CI)**			**21**			**21**	**43.2%**	**0.88 [-0.81, 2.56]**
Heterogeneity: Tau² = 1.24; Chi² = 6.26, df = 1 (P = 0.01); I² = 84%								
Test for overall effect: Z = 1.02 (P = 0.31)								
**1.7.2 Community residents (with sarcopenia)**								
Zhu et al. [3]	15.09	4.44	28	15.16	4.52	27	29.5%	-0.02 [-0.54, 0.51]
**Subtotal (95% CI)**			**28**			**27**	**29.5%**	**-0.02 [-0.54, 0.51]**
Heterogeneity: Not applicable								
Test for overall effect: Z = 0.06 (P = 0.95)								
**1.7.3 Institutionalized residents (healthy)**								
Santin-Medeiros et al. [15]	17.53	5.03	19	18.76	5.83	18	27.3%	-0.22 [-0.87, 0.43]
**Subtotal (95% CI)**			**19**			**18**	**27.3%**	**-0.22 [-0.87, 0.43]**
Heterogeneity: Not applicable								
Test for overall effect: Z = 0.67 (P = 0.50)								
**Total (95% CI)**			**68**			**66**	**100.0%**	**0.30 [-0.41, 1.00]**
Heterogeneity: Tau² = 0.36; Chi² = 10.93, df = 3 (P = 0.01); I² = 73%								
Test for overall effect: Z = 0.83 (P = 0.41)								
Test for subgroup differences: Chi² = 1.45, df = 2 (P = 0.48), I² = 0%								

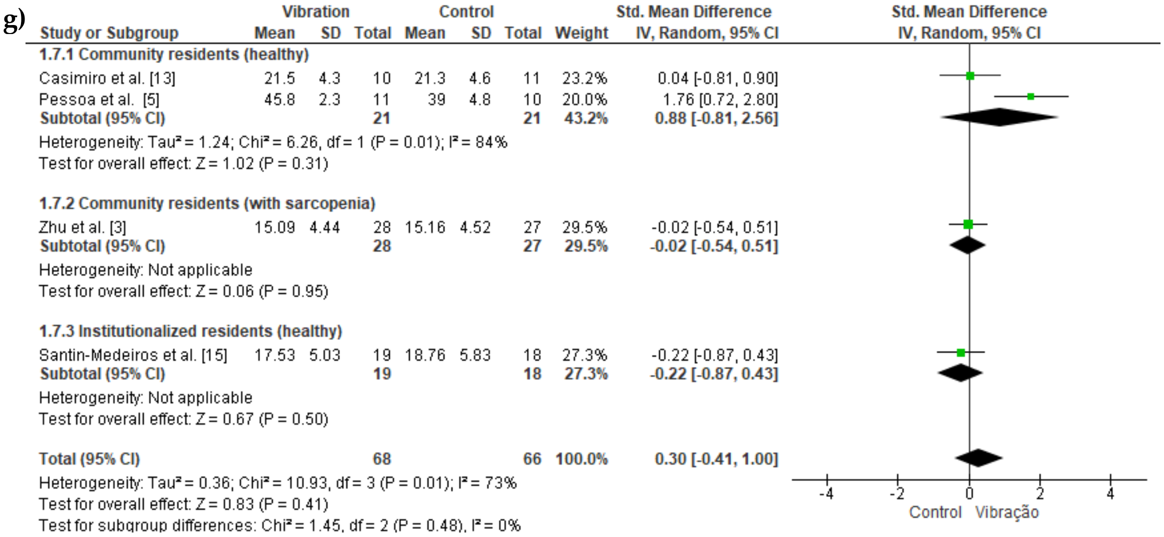

h

Study or Subgroup	Vibration Mean	Vibration SD	Vibration Total	Control Mean	Control SD	Control Total	Weight	Std. Mean Difference IV, Random, 95% CI
**2.1.1 Communit residents (healthy)**								
Rees et al. [33]	8.69	0.79	15	9.73	1.21	15	45.4%	-0.99 [-1.75, -0.23]
**Subtotal (95% CI)**			**15**			**15**	**45.4%**	**-0.99 [-1.75, -0.23]**
Heterogeneity: Not applicable								
Test for overall effect: Z = 2.54 (P = 0.01)								
**2.1.2 Community residents (with sarcopenia)**								
Wei et al. [9]	11.105	4.0544	40	11.4	2.99	20	54.6%	-0.08 [-0.61, 0.46]
**Subtotal (95% CI)**			**40**			**20**	**54.6%**	**-0.08 [-0.61, 0.46]**
Heterogeneity: Not applicable								
Test for overall effect: Z = 0.28 (P = 0.78)								
**Total (95% CI)**			**55**			**35**	**100.0%**	**-0.49 [-1.38, 0.40]**
Heterogeneity: Tau² = 0.30; Chi² = 3.66, df = 1 (P = 0.06); I² = 73%								
Test for overall effect: Z = 1.08 (P = 0.28)								
Test for subgroup differences: Chi² = 3.66, df = 1 (P = 0.06), I² = 72.7%								

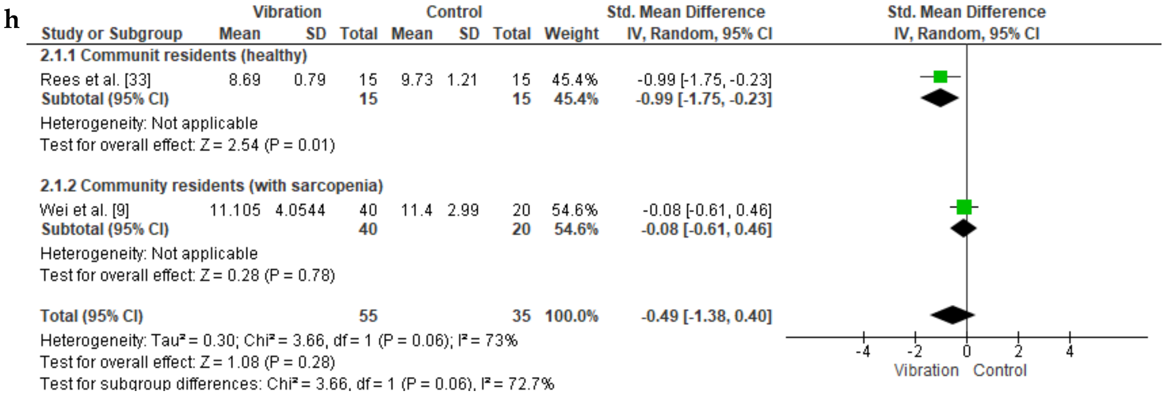

i)

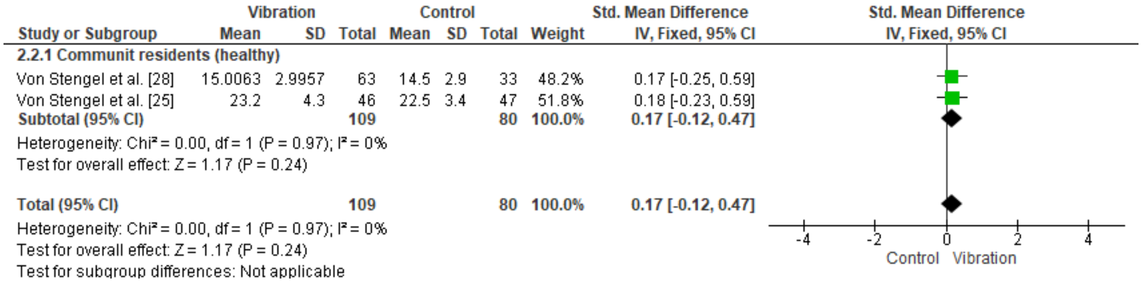

j)

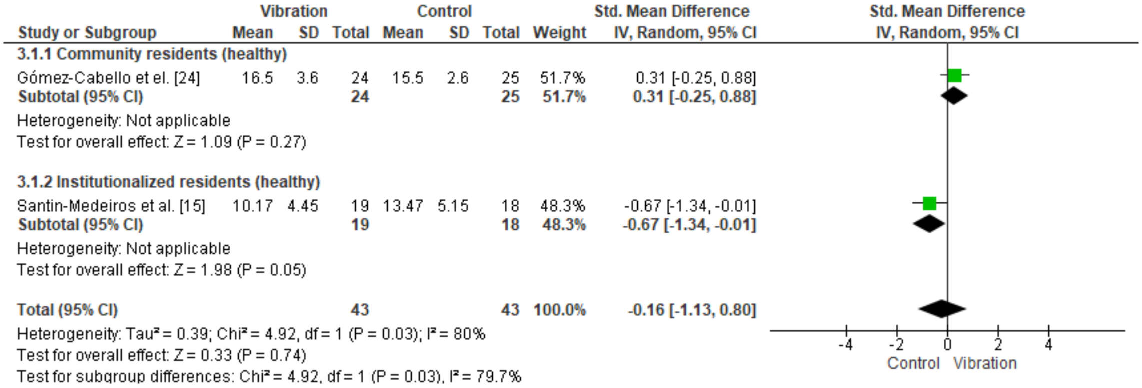

k

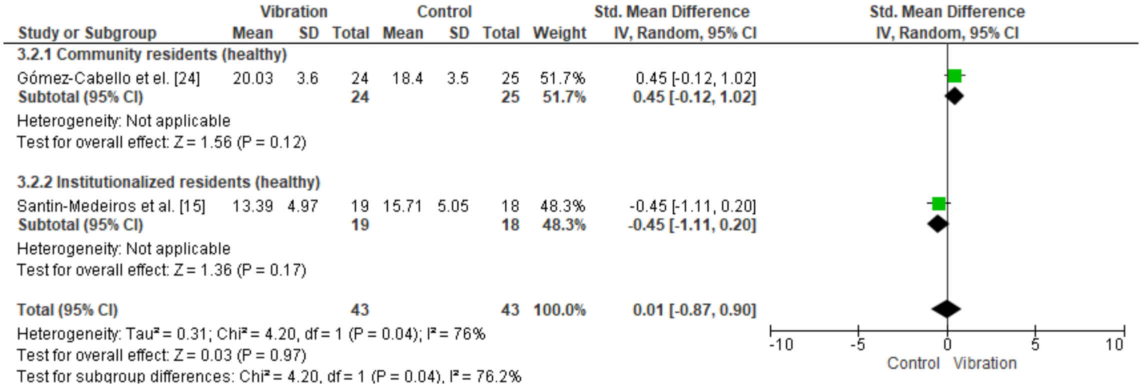

Supplementary Figure S11. Analysis comparing the effectiveness of WBV administered at a high cumulative dose (> 144 minutes) vs. control groups: a) muscle strength of the knee extensors; b) muscle strength of the knee flexors; c) muscular strength of the extensors of the lower limbs; d) muscular strength of the plantar flexors; e) muscular strength of the ankle dorsiflexors; f) muscle strength of the hip flexors; g) handgrip muscle strength; h) five-times-sit-to-stand; i) countermovement jump; j) 30-s sit-to-stand; k) 30-s arm curl.

a)

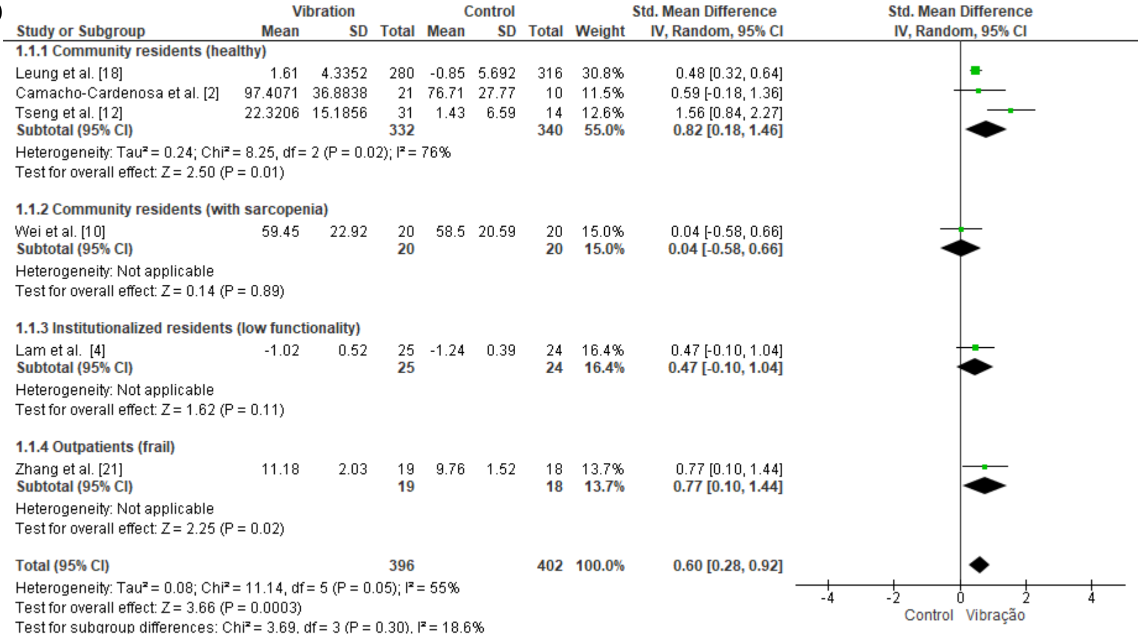
Vibration Control Std. Mean Difference Std. Mean Difference
Study or Subgroup Mean SD Total Mean SD Total Weight IV, Random, 95% CI IV, Random, 95% CI
1.1.1 Community residents (healthy)
Leung et al. [18] 1.61 4.3352 280 -0.85 5.692 316 30.8% 0.48 [0.32, 0.64]
Camacho-Cardenosa et al. [2] 97.4071 36.8838 21 76.71 27.77 10 11.5% 0.59 [-0.18, 1.36]
Tseng et al. [12] 22.3206 15.1856 31 1.43 6.59 14 12.6% 1.56 [0.84, 2.27]
Subtotal (95% CI) 332 340 55.0% 0.82 [0.18, 1.46]
Heterogeneity: Tau² = 0.24; Chi² = 8.25, df = 2 (P = 0.02); I² = 76%
Test for overall effect: Z = 2.50 (P = 0.01)
1.1.2 Community residents (with sarcopenia)
Wei et al. [10] 59.45 22.92 20 58.5 20.59 20 15.0% 0.04 [-0.58, 0.66]
Subtotal (95% CI) 20 20 15.0% 0.04 [-0.58, 0.66]
Heterogeneity: Not applicable
Test for overall effect: Z = 0.14 (P = 0.89)
1.1.3 Institutionalized residents (low functionality)
Lam et al. [4] -1.02 0.52 25 -1.24 0.39 24 16.4% 0.47 [-0.10, 1.04]
Subtotal (95% CI) 25 24 16.4% 0.47 [-0.10, 1.04]
Heterogeneity: Not applicable
Test for overall effect: Z = 1.62 (P = 0.11)
1.1.4 Outpatients (frail)
Zhang et al. [21] 11.18 2.03 19 9.76 1.52 18 13.7% 0.77 [0.10, 1.44]
Subtotal (95% CI) 19 18 13.7% 0.77 [0.10, 1.44]
Heterogeneity: Not applicable
Test for overall effect: Z = 2.25 (P = 0.02)
Total (95% CI) 396 402 100.0% 0.60 [0.28, 0.92]
Heterogeneity: Tau² = 0.08; Chi² = 11.14, df = 5 (P = 0.05); I² = 55%
Test for overall effect: Z = 3.66 (P = 0.0003)
Test for subgroup differences: Chi² = 3.69, df = 3 (P = 0.30), I² = 18.6%
-4 -2 0 2 4
Control Vibração

b

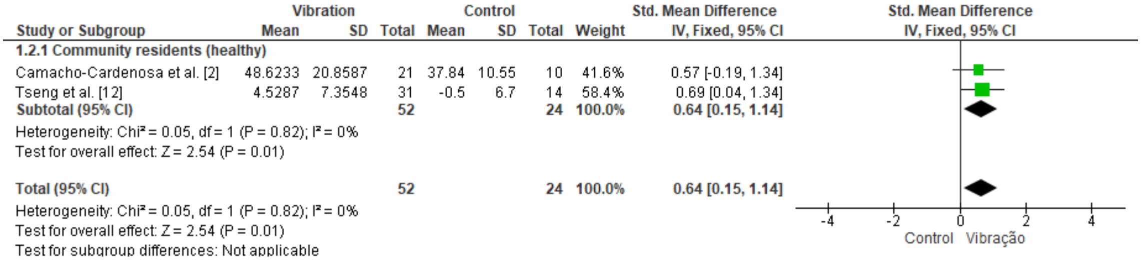
Vibration Control Std. Mean Difference Std. Mean Difference
Study or Subgroup Mean SD Total Mean SD Total Weight IV, Fixed, 95% CI IV, Fixed, 95% CI
1.2.1 Community residents (healthy)
Camacho-Cardenosa et al. [2] 48.6233 20.8587 21 37.84 10.55 10 41.6% 0.57 [-0.19, 1.34]
Tseng et al. [12] 4.5287 7.3548 31 -0.5 6.7 14 58.4% 0.69 [0.04, 1.34]
Subtotal (95% CI) 52 24 100.0% 0.64 [0.15, 1.14]
Heterogeneity: Chi² = 0.05, df = 1 (P = 0.82); I² = 0%
Test for overall effect: Z = 2.54 (P = 0.01)
Total (95% CI) 52 24 100.0% 0.64 [0.15, 1.14]
Heterogeneity: Chi² = 0.05, df = 1 (P = 0.82); I² = 0%
Test for overall effect: Z = 2.54 (P = 0.01)
Test for subgroup differences: Not applicable
-4 -2 0 2 4
Control Vibração

c)

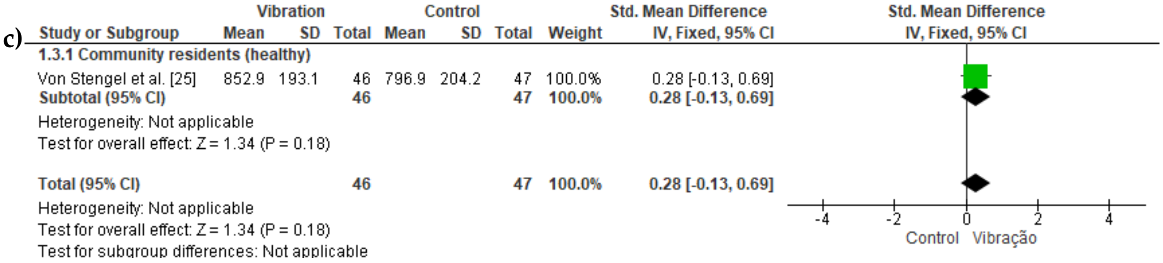
Vibration Control Std. Mean Difference Std. Mean Difference
Study or Subgroup Mean SD Total Mean SD Total Weight IV, Fixed, 95% CI IV, Fixed, 95% CI
1.3.1 Community residents (healthy)
Von Stengel et al. [25] 852.9 193.1 46 796.9 204.2 47 100.0% 0.28 [-0.13, 0.69]
Subtotal (95% CI) 46 47 100.0% 0.28 [-0.13, 0.69]
Heterogeneity: Not applicable
Test for overall effect: Z = 1.34 (P = 0.18)
Total (95% CI) 46 47 100.0% 0.28 [-0.13, 0.69]
Heterogeneity: Not applicable
Test for overall effect: Z = 1.34 (P = 0.18)
Test for subgroup differences: Not applicable
-4 -2 0 2 4
Control Vibração

d

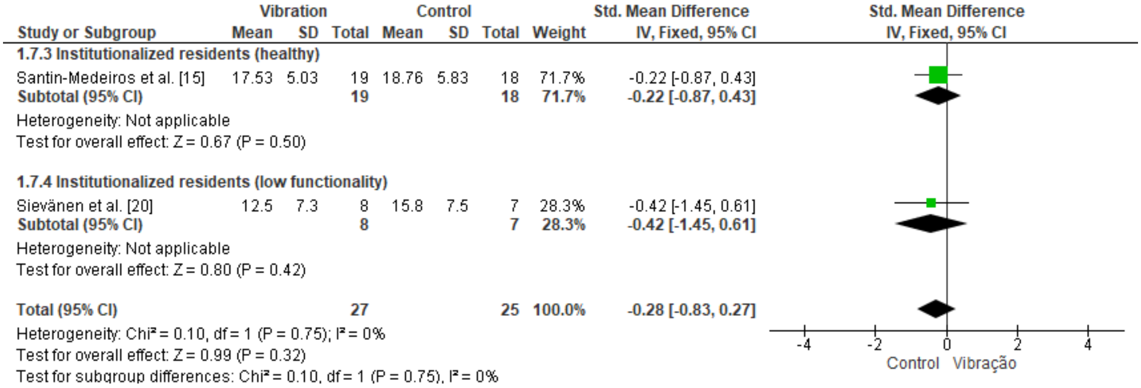
Vibration Control Std. Mean Difference Std. Mean Difference
Study or Subgroup Mean SD Total Mean SD Total Weight IV, Fixed, 95% CI IV, Fixed, 95% CI
1.7.3 Institutionalized residents (healthy)
Santin-Medeiros et al. [15] 17.53 5.03 19 18.76 5.83 18 71.7% -0.22 [-0.87, 0.43]
Subtotal (95% CI) 19 18 71.7% -0.22 [-0.87, 0.43]
Heterogeneity: Not applicable
Test for overall effect: Z = 0.67 (P = 0.50)
1.7.4 Institutionalized residents (low functionality)
Sievänen et al. [20] 12.5 7.3 8 15.8 7.5 7 28.3% -0.42 [-1.45, 0.61]
Subtotal (95% CI) 8 7 28.3% -0.42 [-1.45, 0.61]
Heterogeneity: Not applicable
Test for overall effect: Z = 0.80 (P = 0.42)
Total (95% CI) 27 25 100.0% -0.28 [-0.83, 0.27]
Heterogeneity: Chi² = 0.10, df = 1 (P = 0.75); I² = 0%
Test for overall effect: Z = 0.99 (P = 0.32)
Test for subgroup differences: Chi² = 0.10, df = 1 (P = 0.75), I² = 0%
-4 -2 0 2 4
Control Vibração

e)

Study or Subgroup	Vibration Mean	Vibration SD	Vibration Total	Control Mean	Control SD	Control Total	Weight	Std. Mean Difference IV, Fixed, 95% CI
**2.1.1 Communit residents (healthy)**								
Furness et al. [31]	13.1224	2.6776	55	13.35	1.12	18	37.9%	-0.09 [-0.63, 0.44]
**Subtotal (95% CI)**			**55**			**18**	**37.9%**	**-0.09 [-0.63, 0.44]**
Heterogeneity: Not applicable								
Test for overall effect: Z = 0.35 (P = 0.73)								
**2.1.2 Community residents (with sarcopenia)**								
Wei et al. [9]	11.75	5.26	20	11.4	2.99	20	27.9%	0.08 [-0.54, 0.70]
**Subtotal (95% CI)**			**20**			**20**	**27.9%**	**0.08 [-0.54, 0.70]**
Heterogeneity: Not applicable								
Test for overall effect: Z = 0.25 (P = 0.80)								
**2.1.3 Institutionalized residents (low functionality)**								
Lam et al. [4]	27.3	12.9	25	29	9.8	24	34.2%	-0.15 [-0.71, 0.42]
**Subtotal (95% CI)**			**25**			**24**	**34.2%**	**-0.15 [-0.71, 0.42]**
Heterogeneity: Not applicable								
Test for overall effect: Z = 0.51 (P = 0.61)								
**Total (95% CI)**			**100**			**62**	**100.0%**	**-0.06 [-0.39, 0.26]**
Heterogeneity: Chi² = 0.30, df = 2 (P = 0.86); I² = 0%								
Test for overall effect: Z = 0.38 (P = 0.71)								
Test for subgroup differences: Chi² = 0.30, df = 2 (P = 0.86), I² = 0%								

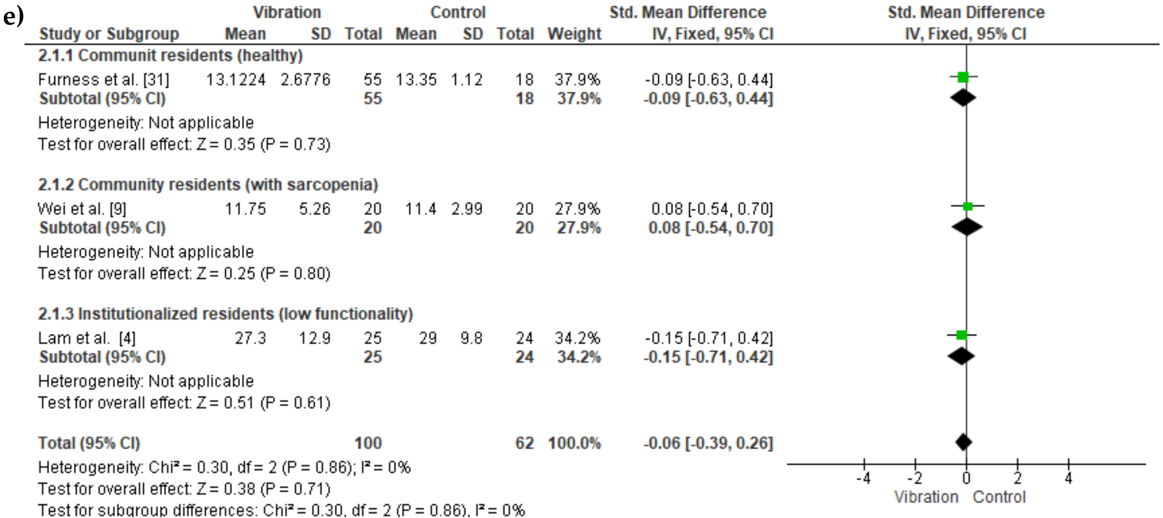

f)

Study or Subgroup	Vibration Mean	Vibration SD	Vibration Total	Control Mean	Control SD	Control Total	Weight	Std. Mean Difference IV, Fixed, 95% CI
**2.2.1 Communit residents (healthy)**								
Von Stengel et al. [25]	23.2	4.3	46	22.5	3.4	47	100.0%	0.18 [-0.23, 0.59]
**Subtotal (95% CI)**			**46**			**47**	**100.0%**	**0.18 [-0.23, 0.59]**
Heterogeneity: Not applicable								
Test for overall effect: Z = 0.86 (P = 0.39)								
**Total (95% CI)**			**46**			**47**	**100.0%**	**0.18 [-0.23, 0.59]**
Heterogeneity: Not applicable								
Test for overall effect: Z = 0.86 (P = 0.39)								
Test for subgroup differences: Not applicable								

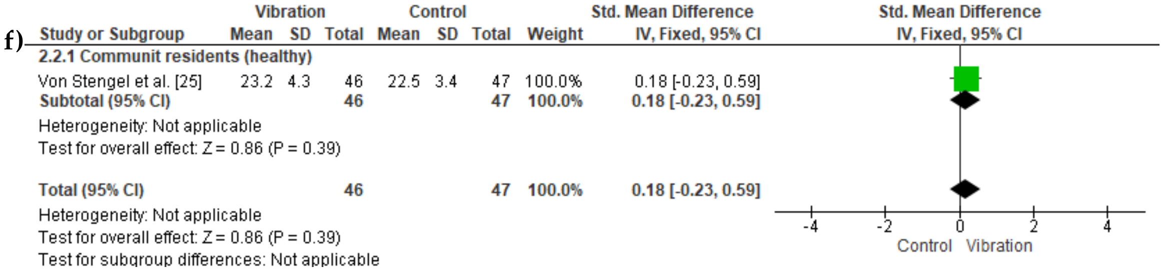

g)

Study or Subgroup	Vibration Mean	Vibration SD	Vibration Total	Control Mean	Control SD	Control Total	Weight	Std. Mean Difference IV, Random, 95% CI
**3.1.1 Community residents (healthy)**								
Dudoniene et al. [23]	17.4	3.3	20	16.8	2.4	20	34.2%	0.20 [-0.42, 0.83]
**Subtotal (95% CI)**			**20**			**20**	**34.2%**	**0.20 [-0.42, 0.83]**
Heterogeneity: Not applicable								
Test for overall effect: Z = 0.64 (P = 0.52)								
**3.1.2 Institutionalized residents (healthy)**								
Santin-Medeiros et al. [15]	10.17	4.45	19	13.47	5.15	18	32.7%	-0.67 [-1.34, -0.01]
**Subtotal (95% CI)**			**19**			**18**	**32.7%**	**-0.67 [-1.34, -0.01]**
Heterogeneity: Not applicable								
Test for overall effect: Z = 1.98 (P = 0.05)								
**3.1.3 Outpatients (frail)**								
Zhang et al. [21]	5.26	4.09	19	3.56	3.45	18	33.1%	0.44 [-0.21, 1.09]
**Subtotal (95% CI)**			**19**			**18**	**33.1%**	**0.44 [-0.21, 1.09]**
Heterogeneity: Not applicable								
Test for overall effect: Z = 1.32 (P = 0.19)								
**Total (95% CI)**			**58**			**56**	**100.0%**	**-0.01 [-0.66, 0.65]**
Heterogeneity: Tau² = 0.22; Chi² = 6.07, df = 2 (P = 0.05); I² = 67%								
Test for overall effect: Z = 0.02 (P = 0.99)								
Test for subgroup differences: Chi² = 6.07, df = 2 (P = 0.05), I² = 67.1%								

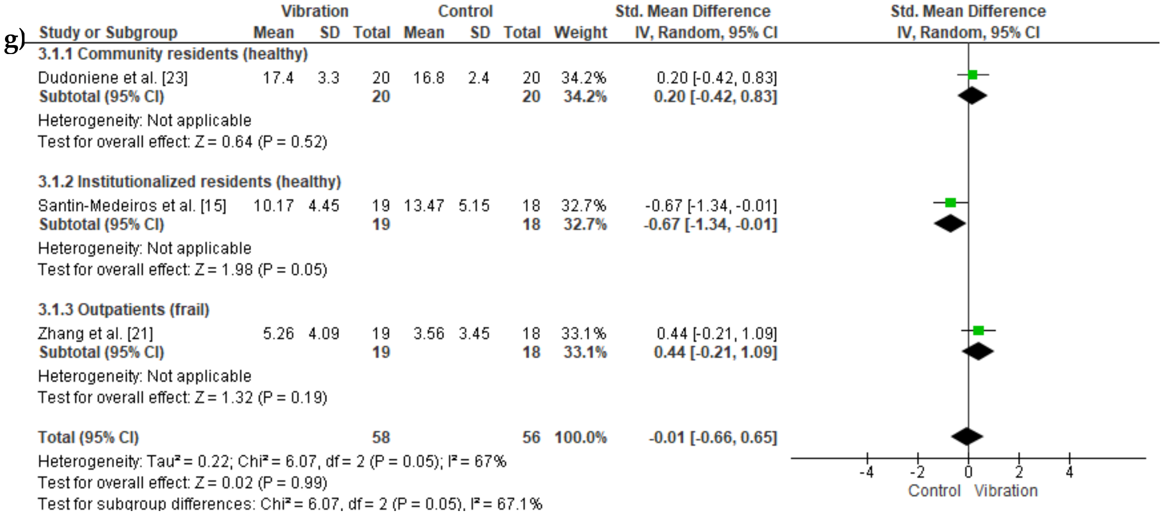

h

Study or Subgroup	Vibration Mean	Vibration SD	Vibration Total	Control Mean	Control SD	Control Total	Weight	Std. Mean Difference IV, Fixed, 95% CI
**3.2.2 Institutionalized residents (healthy)**								
Santin-Medeiros et al. [15]	13.39	4.97	19	15.71	5.05	18	100.0%	-0.45 [-1.11, 0.20]
**Subtotal (95% CI)**			**19**			**18**	**100.0%**	**-0.45 [-1.11, 0.20]**
Heterogeneity: Not applicable								
Test for overall effect: Z = 1.36 (P = 0.17)								
**Total (95% CI)**			**19**			**18**	**100.0%**	**-0.45 [-1.11, 0.20]**
Heterogeneity: Not applicable								
Test for overall effect: Z = 1.36 (P = 0.17)								
Test for subgroup differences: Not applicable								

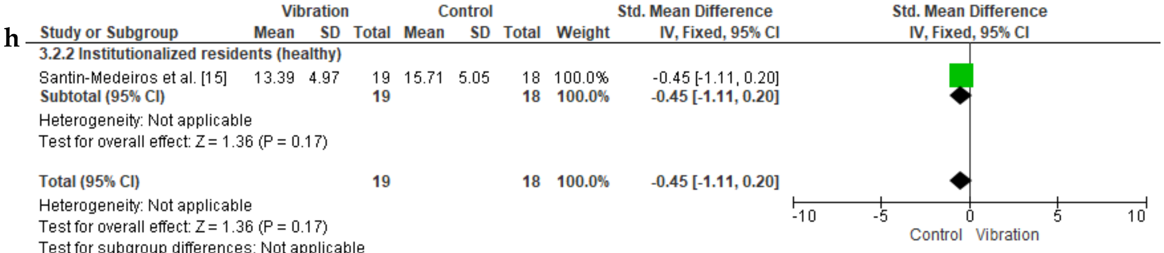

Supplementary Figure S12. Analysis comparing the effectiveness of WBV administered at low magnitude (≤ 4.4 g) vs. control groups: a) muscle strength of the knee extensors; b) muscle strength of the knee flexors; c) muscular strength of the extensors of the lower limbs; d) handgrip muscle strength; e) five-times-sit-to-stand; f) countermovement jump; g) 30-s sit-to-stand; h) 30-s arm curl.

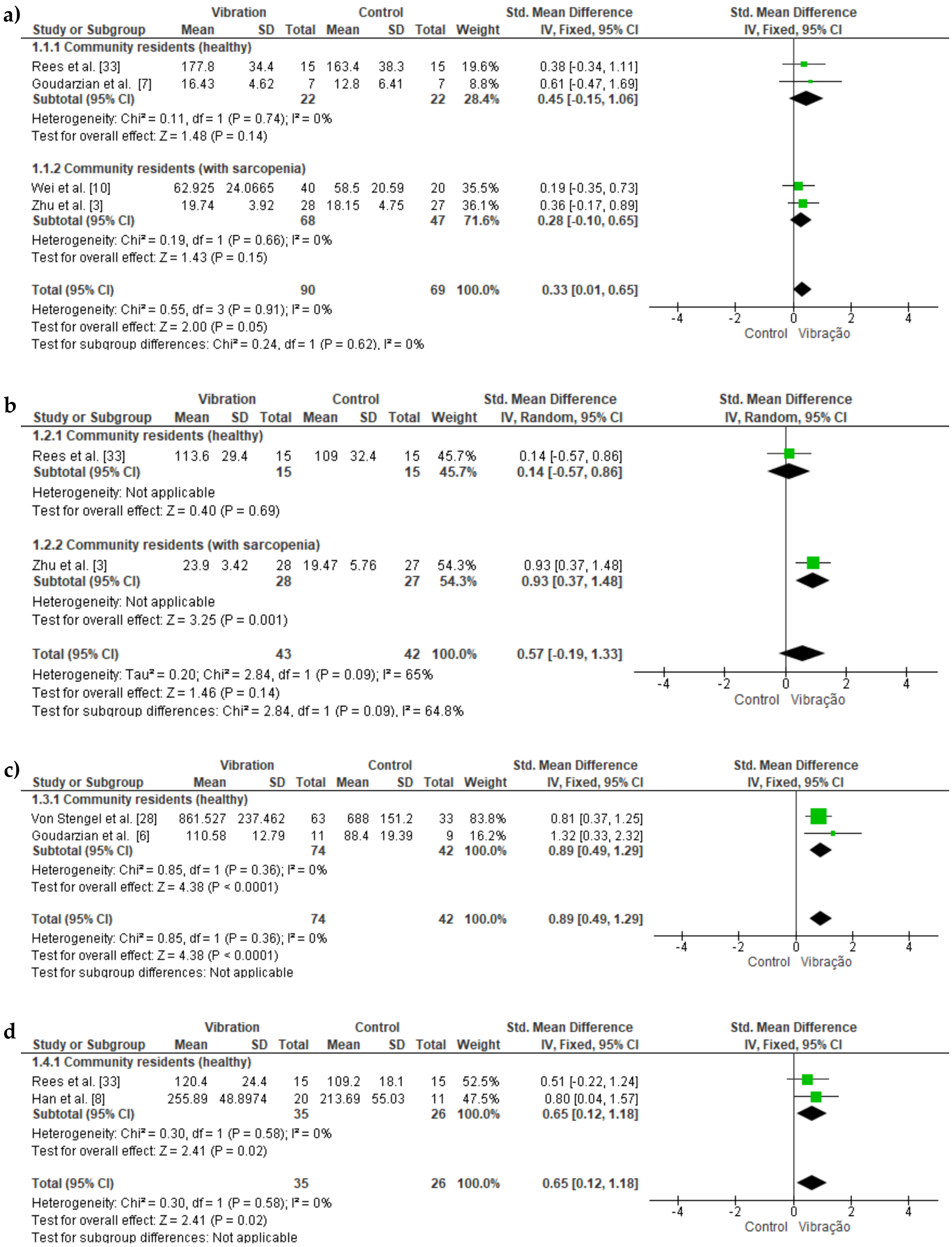
a)
Vibration
Control
Std. Mean Difference
Std. Mean Difference
Study or Subgroup Mean SD Total Mean SD Total Weight IV, Fixed, 95% CI
IV, Fixed, 95% CI
1.1.1 Community residents (healthy)
Rees et al. [33] 177.8 34.4 15 163.4 38.3 15 19.6% 0.38 [-0.34, 1.11]
Goudarzian et al. [7] 16.43 4.62 7 12.8 6.41 7 8.8% 0.61 [-0.47, 1.69]
Subtotal (95% CI) 22 22 28.4% 0.45 [-0.15, 1.06]
Heterogeneity: Chi² = 0.11, df = 1 (P = 0.74); I² = 0%
Test for overall effect: Z = 1.48 (P = 0.14)
1.1.2 Community residents (with sarcopenia)
Wei et al. [10] 62.925 24.0665 40 58.5 20.59 20 35.5% 0.19 [-0.35, 0.73]
Zhu et al. [3] 19.74 3.92 28 18.15 4.75 27 36.1% 0.36 [-0.17, 0.89]
Subtotal (95% CI) 68 47 71.6% 0.28 [-0.10, 0.65]
Heterogeneity: Chi² = 0.19, df = 1 (P = 0.66); I² = 0%
Test for overall effect: Z = 1.43 (P = 0.15)
Total (95% CI) 90 69 100.0% 0.33 [0.01, 0.65]
Heterogeneity: Chi² = 0.55, df = 3 (P = 0.91); I² = 0%
Test for overall effect: Z = 2.00 (P = 0.05)
Test for subgroup differences: Chi² = 0.24, df = 1 (P = 0.62), I² = 0%
-4 -2 0 2 4
Control Vibração
b
Vibration
Control
Std. Mean Difference
Std. Mean Difference
Study or Subgroup Mean SD Total Mean SD Total Weight IV, Random, 95% CI
IV, Random, 95% CI
1.2.1 Community residents (healthy)
Rees et al. [33] 113.6 29.4 15 109 32.4 15 45.7% 0.14 [-0.57, 0.86]
Subtotal (95% CI) 15 15 45.7% 0.14 [-0.57, 0.86]
Heterogeneity: Not applicable
Test for overall effect: Z = 0.40 (P = 0.69)
1.2.2 Community residents (with sarcopenia)
Zhu et al. [3] 23.9 3.42 28 19.47 5.76 27 54.3% 0.93 [0.37, 1.48]
Subtotal (95% CI) 28 27 54.3% 0.93 [0.37, 1.48]
Heterogeneity: Not applicable
Test for overall effect: Z = 3.25 (P = 0.001)
Total (95% CI) 43 42 100.0% 0.57 [-0.19, 1.33]
Heterogeneity: Tau² = 0.20; Chi² = 2.84, df = 1 (P = 0.09); I² = 65%
Test for overall effect: Z = 1.46 (P = 0.14)
Test for subgroup differences: Chi² = 2.84, df = 1 (P = 0.09), I² = 64.8%
-4 -2 0 2 4
Control Vibração
c)
Vibration
Control
Std. Mean Difference
Std. Mean Difference
Study or Subgroup Mean SD Total Mean SD Total Weight IV, Fixed, 95% CI
IV, Fixed, 95% CI
1.3.1 Community residents (healthy)
Von Stengel et al. [28] 861.527 237.462 63 688 151.2 33 83.8% 0.81 [0.37, 1.25]
Goudarzian et al. [6] 110.58 12.79 11 88.4 19.39 9 16.2% 1.32 [0.33, 2.32]
Subtotal (95% CI) 74 42 100.0% 0.89 [0.49, 1.29]
Heterogeneity: Chi² = 0.85, df = 1 (P = 0.36); I² = 0%
Test for overall effect: Z = 4.38 (P < 0.0001)
Total (95% CI) 74 42 100.0% 0.89 [0.49, 1.29]
Heterogeneity: Chi² = 0.85, df = 1 (P = 0.36); I² = 0%
Test for overall effect: Z = 4.38 (P < 0.0001)
Test for subgroup differences: Not applicable
-4 -2 0 2 4
Control Vibração
d
Vibration
Control
Std. Mean Difference
Std. Mean Difference
Study or Subgroup Mean SD Total Mean SD Total Weight IV, Fixed, 95% CI
IV, Fixed, 95% CI
1.4.1 Community residents (healthy)
Rees et al. [33] 120.4 24.4 15 109.2 18.1 15 52.5% 0.51 [-0.22, 1.24]
Han et al. [8] 255.89 48.8974 20 213.69 55.03 11 47.5% 0.80 [0.04, 1.57]
Subtotal (95% CI) 35 26 100.0% 0.65 [0.12, 1.18]
Heterogeneity: Chi² = 0.30, df = 1 (P = 0.58); I² = 0%
Test for overall effect: Z = 2.41 (P = 0.02)
Total (95% CI) 35 26 100.0% 0.65 [0.12, 1.18]
Heterogeneity: Chi² = 0.30, df = 1 (P = 0.58); I² = 0%
Test for overall effect: Z = 2.41 (P = 0.02)
Test for subgroup differences: Not applicable
-4 -2 0 2 4
Control Vibração

e)

Study or Subgroup	Vibration Mean	Vibration SD	Vibration Total	Control Mean	Control SD	Control Total	Weight	Std. Mean Difference IV, Random, 95% CI
**1.5.1 Community residents (healthy)**								
Rees et al. [33]	36.1	7	15	36.8	6.1	15	45.1%	-0.10 [-0.82, 0.61]
**Subtotal (95% CI)**			**15**			**15**	**45.1%**	**-0.10 [-0.82, 0.61]**
Heterogeneity: Not applicable								
Test for overall effect: Z = 0.28 (P = 0.78)								
**1.5.2 Community residents (with sarcopenia)**								
Zhu et al. [3]	20.07	3.98	28	17.69	3.11	27	54.9%	0.66 [0.11, 1.20]
**Subtotal (95% CI)**			**28**			**27**	**54.9%**	**0.66 [0.11, 1.20]**
Heterogeneity: Not applicable								
Test for overall effect: Z = 2.36 (P = 0.02)								
**Total (95% CI)**			**43**			**42**	**100.0%**	**0.31 [-0.43, 1.05]**
Heterogeneity: Tau² = 0.18; Chi² = 2.74, df = 1 (P = 0.10); I² = 63%								
Test for overall effect: Z = 0.83 (P = 0.41)								
Test for subgroup differences: Chi² = 2.74, df = 1 (P = 0.10), I² = 63.5%								

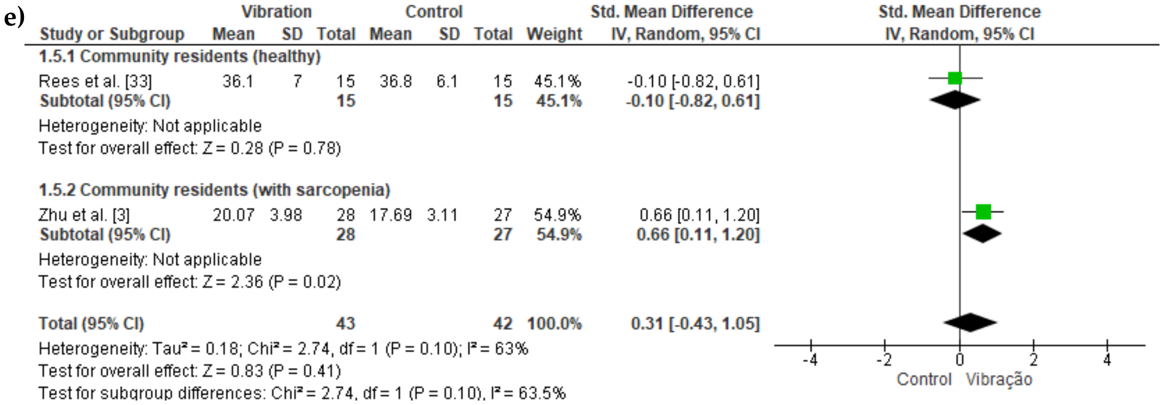

f)

Study or Subgroup	Vibration Mean	Vibration SD	Vibration Total	Control Mean	Control SD	Control Total	Weight	Std. Mean Difference IV, Fixed, 95% CI
**1.6.1 Community residents (healthy)**								
Rees et al. [33]	143.8	29.2	15	140.6	27.2	15	35.4%	0.11 [-0.61, 0.83]
**Subtotal (95% CI)**			**15**			**15**	**35.4%**	**0.11 [-0.61, 0.83]**
Heterogeneity: Not applicable								
Test for overall effect: Z = 0.30 (P = 0.76)								
**1.6.2 Community residents (with sarcopenia)**								
Zhu et al. [3]	22.17	5.77	28	20.94	6.33	27	64.6%	0.20 [-0.33, 0.73]
**Subtotal (95% CI)**			**28**			**27**	**64.6%**	**0.20 [-0.33, 0.73]**
Heterogeneity: Not applicable								
Test for overall effect: Z = 0.74 (P = 0.46)								
**Total (95% CI)**			**43**			**42**	**100.0%**	**0.17 [-0.26, 0.59]**
Heterogeneity: Chi² = 0.04, df = 1 (P = 0.84); I² = 0%								
Test for overall effect: Z = 0.78 (P = 0.44)								
Test for subgroup differences: Chi² = 0.04, df = 1 (P = 0.84), I² = 0%								

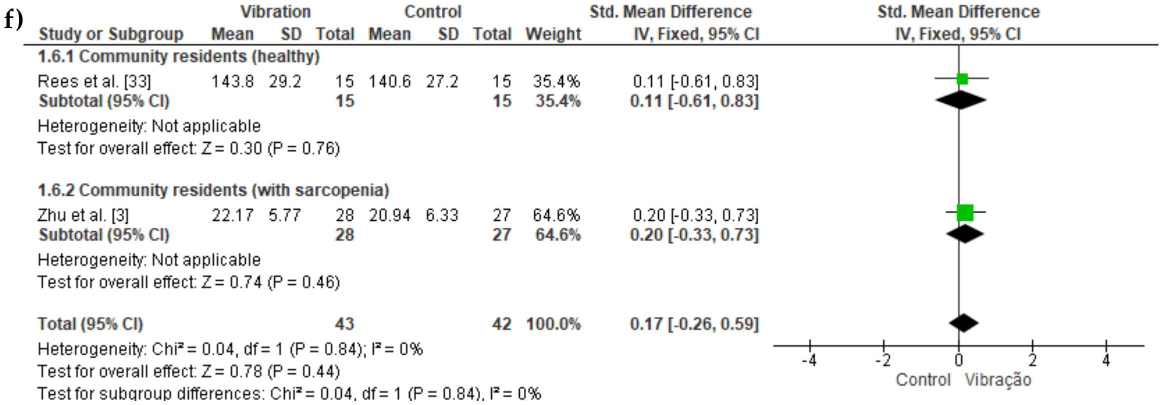

g)

Study or Subgroup	Vibration Mean	Vibration SD	Vibration Total	Control Mean	Control SD	Control Total	Weight	Std. Mean Difference IV, Random, 95% CI
**1.7.1 Community residents (healthy)**								
Casimiro et al. [13]	21.5	4.3	10	21.3	4.6	11	20.0%	0.04 [-0.81, 0.90]
Pessoa et al. [5]	45.8	2.3	11	39	4.8	10	16.6%	1.76 [0.72, 2.80]
**Subtotal (95% CI)**			**21**			**21**	**36.5%**	**0.88 [-0.81, 2.56]**
Heterogeneity: Tau² = 1.24; Chi² = 6.26, df = 1 (P = 0.01); I² = 84%								
Test for overall effect: Z = 1.02 (P = 0.31)								
**1.7.2 Community residents (with sarcopenia)**								
Zhu et al. [3]	15.09	4.44	28	15.16	4.52	27	27.2%	-0.02 [-0.54, 0.51]
**Subtotal (95% CI)**			**28**			**27**	**27.2%**	**-0.02 [-0.54, 0.51]**
Heterogeneity: Not applicable								
Test for overall effect: Z = 0.06 (P = 0.95)								
**1.7.3 Institutionalized residents (healthy)**								
Goudarzian et al. [7]	18.64	5.03	7	17.57	2.84	7	16.4%	0.25 [-0.81, 1.30]
**Subtotal (95% CI)**			**7**			**7**	**16.4%**	**0.25 [-0.81, 1.30]**
Heterogeneity: Not applicable								
Test for overall effect: Z = 0.46 (P = 0.65)								
**1.7.4 Institutionalized residents (low functionality)**								
Bautmans et al. [34]	44.6	20.8	10	45.6	25.4	11	20.0%	-0.04 [-0.90, 0.82]
**Subtotal (95% CI)**			**10**			**11**	**20.0%**	**-0.04 [-0.90, 0.82]**
Heterogeneity: Not applicable								
Test for overall effect: Z = 0.09 (P = 0.92)								
**Total (95% CI)**			**66**			**66**	**100.0%**	**0.33 [-0.26, 0.91]**
Heterogeneity: Tau² = 0.25; Chi² = 9.73, df = 4 (P = 0.05); I² = 59%								
Test for overall effect: Z = 1.10 (P = 0.27)								
Test for subgroup differences: Chi² = 1.15, df = 3 (P = 0.76), I² = 0%								

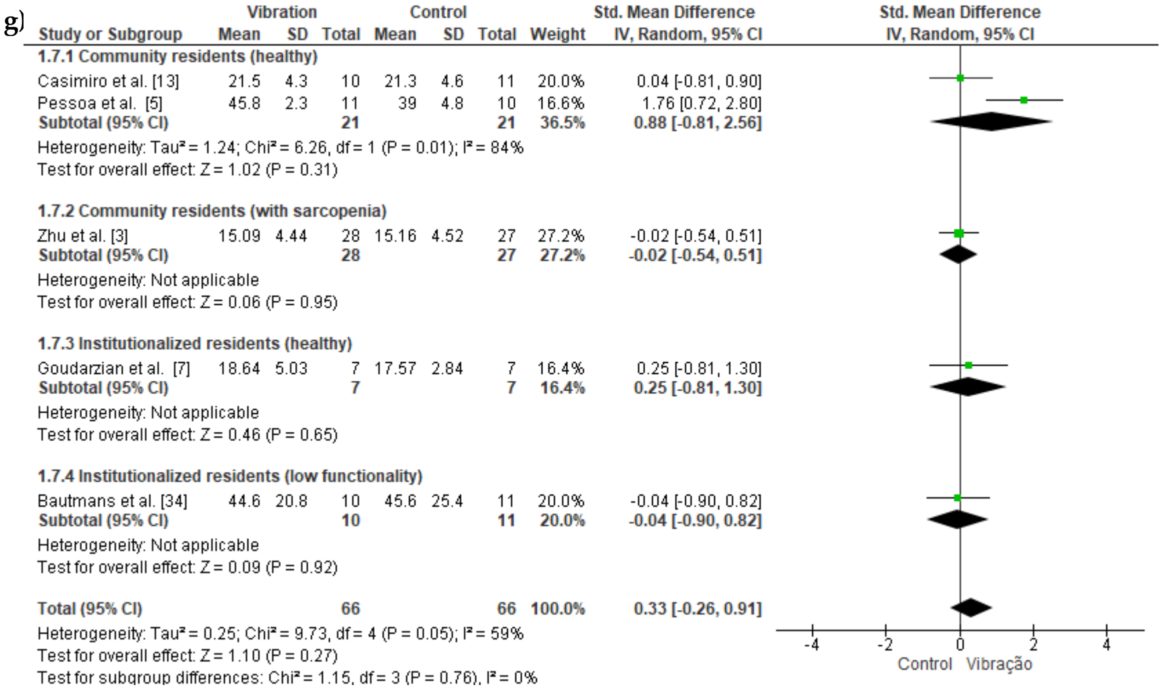

h

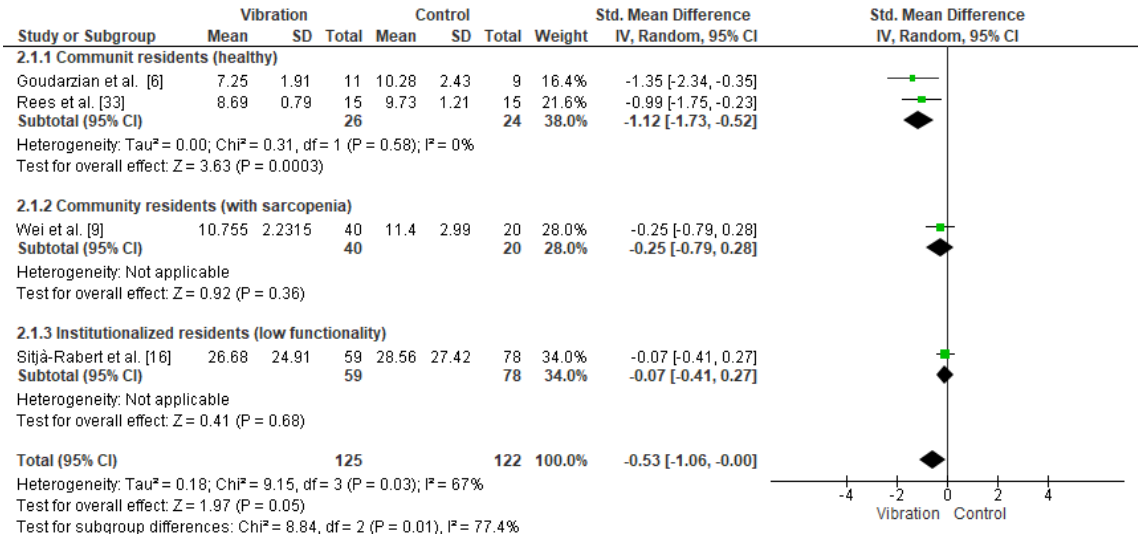

i)

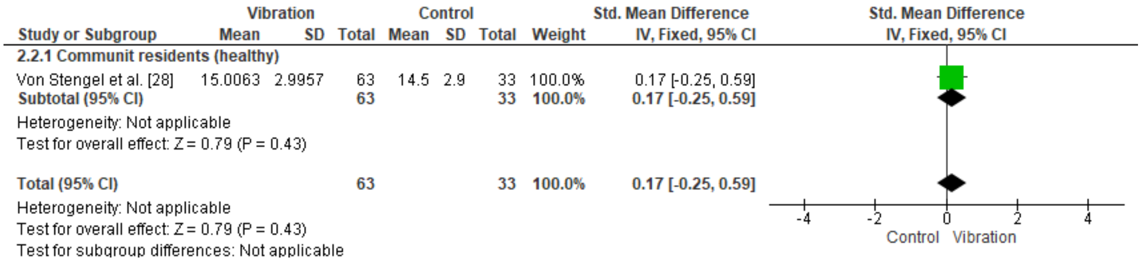

j)

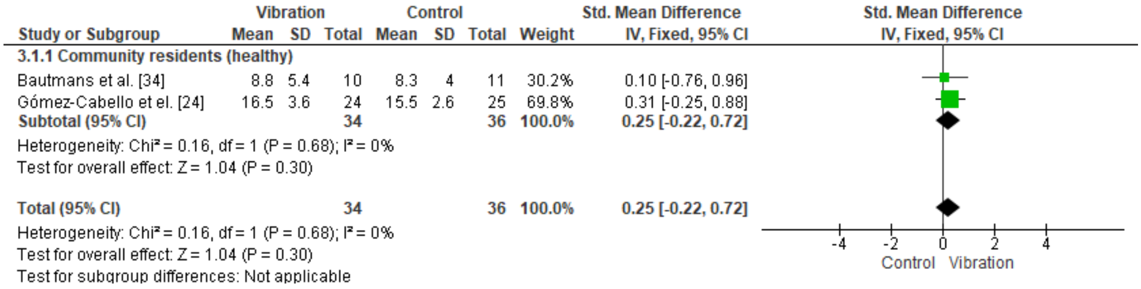

k

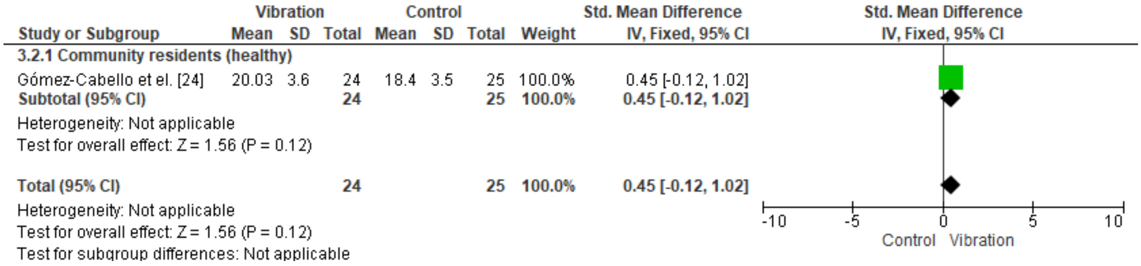

Supplementary Figure S13. Analysis comparing the effectiveness of WBV administered at high magnitude (> 4.4 g) vs. control groups: a) muscle strength of the knee extensors; b) muscle strength of the knee flexors; c) muscular strength of the extensors of the lower limbs; d) muscular strength of the plantar flexors; e) muscular strength of the ankle dorsiflexors; f) muscle strength of the hip flexors; g) handgrip muscle strength; h) Five-times-sit-to-stand; i) countermovement jump; j) 30-s sit-to-stand; k) 30-s arm curl.
